# A Hooker Oxygenase Archetype in Polyketide Biosynthesis Challenging the Baeyer–Villiger Monooxygenase Paradigm

**DOI:** 10.1021/jacs.5c21759

**Published:** 2026-01-26

**Authors:** Heiner G. Weddeling, Sven T. Sowa, Elena Bialas, Sven Reese, Christian Merten, Markus Lill, Andreas Bechthold, Robin Teufel

**Affiliations:** † Pharmaceutical Biology, Department of Pharmaceutical Sciences, 27209 University of Basel, Klingelbergstrasse 50, Basel 4056, Switzerland; ‡ Department of Pharmaceutical Biology and Biotechnology, Institute of Pharmaceutical Sciences, 9174 Albert-Ludwigs-Universität Freiburg, Freiburg 79104, Germany; § 9142 Fakultät für Chemie und Biochemie, Organische Chemie II, Universitätsstraße 150, Bochum 44801, Germany; ∥ Computational Pharmacy, Department of Pharmaceutical Sciences, University of Basel, Klingelbergstrasse 50, Basel 4056, Switzerland

## Abstract

Aromatic polyketides from Actinobacteria are structurally complex bioactive natural products with significant therapeutic potential, whose biosynthesis involves polyketide chain assembly, keto reduction, cyclization, and aromatization. This is followed by pathway-specific enzymatic tailoring steps, occasionally including rare oxidative rearrangements of the carbon skeleton, as exemplified by the rishirilides. In this study, we investigate RslO9, a flavin-dependent tailoring key enzyme of rishirilide biosynthesis, previously hypothesized to facilitate a lactone-forming Baeyer–Villiger oxidation of the rishirilide naphthoquinone core and subsequent intramolecular aldol condensation. Through detailed investigation of RslO9’s mechanism, structural features, and substrate scope, we unexpectedly found that the naphthoquinone moiety of the non-natural substrate lapachol undergoes hydroxylation followed by a benzilic acid rearrangement, producing the Hooker intermediate–a hallmark of the intricate Hooker oxidation. Our data support a similar alkyl migration mechanism for RslO9’s native substrate, upending its prior classification as a Baeyer–Villiger monooxygenase and challenging the proposed role of related enzymes while also providing a novel framework for exploring their catalytic roles.

## Graphical abstract

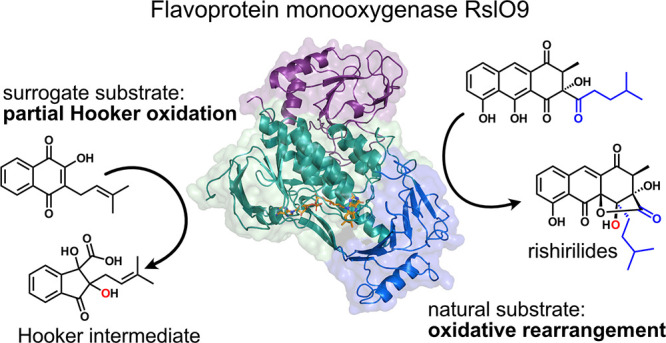

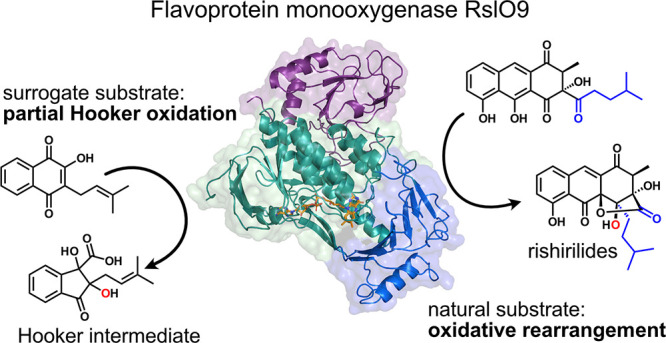

## Introduction

Actinobacteria are prolific producers of a wide variety of natural products,^1^ such as the aromatic polyketides produced by type II polyketide synthases (a heterodimer of ketosynthase (KS)_α_ and KS_β_ in conjunction with an acyl-carrier protein (ACP)), as well as additional core and auxiliary enzymes. These compounds are structurally complex and of high medical relevance, as illustrated by the tetracyclines (antibiotics) and anthracyclines (anticancer drugs).^2^ Their biosynthesis first involves the formation of a reactive poly-β-keto (=polyketide) chain by iterative Claisen condensations that is subsequently cyclized to an aromatic, polycyclic carbon backbone. The following modification of the backbone by pathway-specific tailoring enzymes allows the structural and functional diversification of these compounds and often involves oxidoreductases, e.g., flavoprotein monooxygenases (FPMOs).^2–5^ In particular, members of the so-called group A FPMOs^6^ seem to adopt a central role in this process, typically by acting as aromatic hydroxylases or more rarely as presumed Baeyer–Villiger monooxygenases (BVMOs), whose oxygen-transfer reactions can trigger subsequent skeletal rearrangements.^3,6–11^ A notable example occurs during the biosynthesis of rishirilide A (**1**) in *Streptomyces bottropensis*, where group A FPMO RslO9 catalyzes an oxidative backbone rearrangement and formation of the distinct bridged ring system. Originally discovered in an α2-macroglobulin inhibitor screen, rishirilides feature glutathione-S-transferase inhibitory as well as antimicrobial activity.^12^ Their biosynthesis involves a noncanonical isohexanoyl starter unit (produced from an isobutyryl-CoA precursor and ultimately becoming part of the isopentyl substituent in the final products)^13–15^ that is condensed with a total of eight malonyl–CoA-derived acetate extender units. After elongation, the nascent linear polyketide chain undergoes ketoreduction and formation of the first ring, as also observed for the anthra- and angucyclines. Following that, multiple cyclases form the three-ring backbone before further enzymatic modification takes place.^15^ Final tailoring steps are performed by the multitasking group A FPMO RslO9 together with ketoreductase RslO8 that convert the last shared pathway intermediate RSHO9 (**5**) via intermediate **6** into the pathway products (**1**), rishirilides B (**2**), and D (**3**) as well as lupinacidin A (**4**) (Figure 1A), as verified by previous gene deletion studies and detailed *in vitro* enzyme assays.^10^ Recently, the biosynthetically and structurally related tatiomicin from*Amycolatopsis* sp. was shown to feature an identically configured bridged ring system, likely formed by the RslO9 homologue TatO9; cytological profiling suggested that tatiomicin’s antibiotic activity against Gram-positive strains is caused by membrane depolarization and chromosomal decondensation.^16^

**Figure 1:**
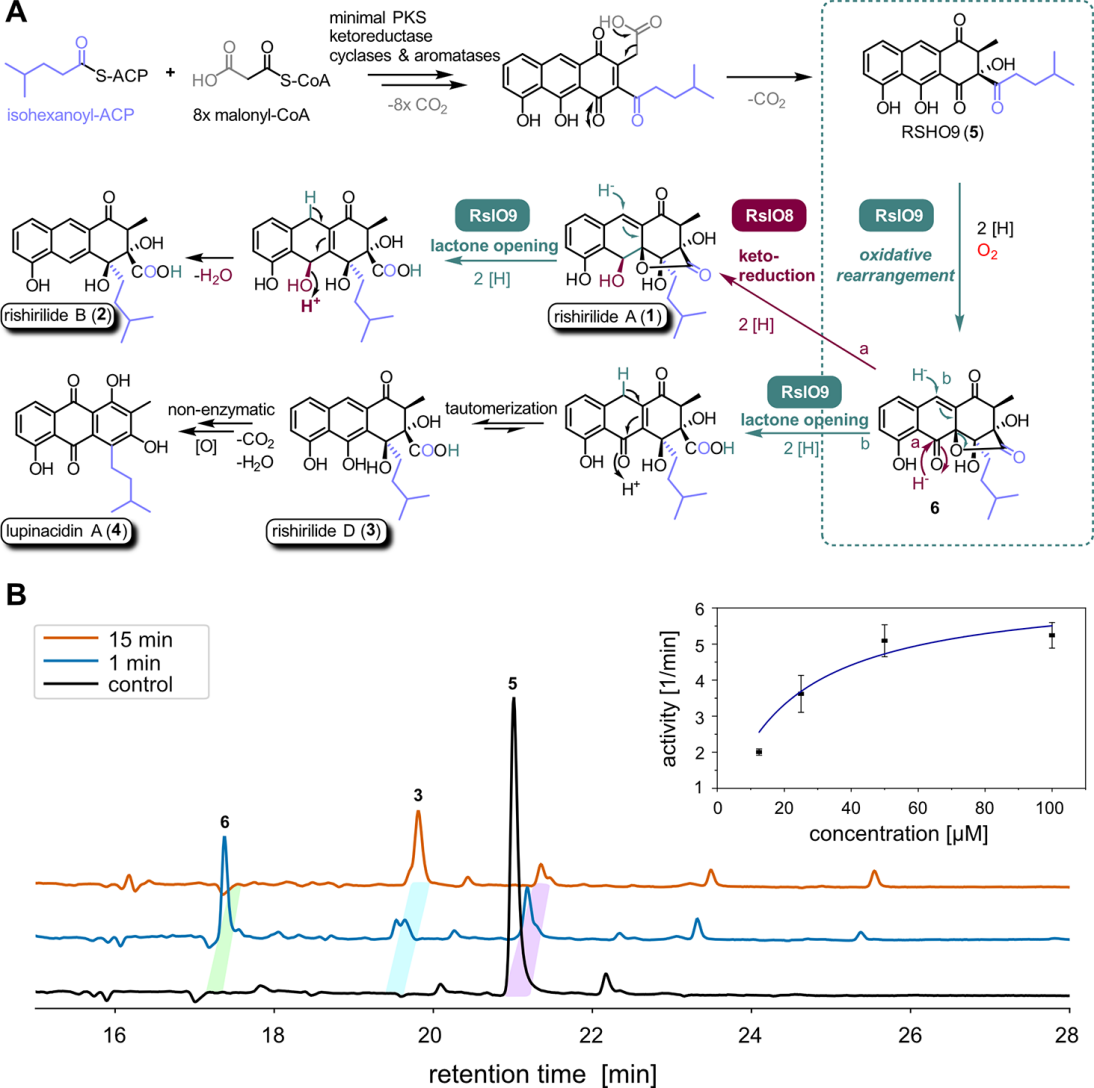
A) Simplified overview of rishirilide biosynthesis and conversion of intermediate **5** into **1**, **2**, **3**, and **4**. FPMO RslO9 acts as the key enzyme for structural diversification by catalyzing an oxidative backbone rearrangement (highlighted in the dashed box and investigated in this work). The resulting intermediate **6** can then undergo an additional ketoreduction mediated by RslO8 to produce **1** that can be further converted into **2** by reductive lactone opening (pathway a), a step that can also take place without prior ketoreduction and then affords **3** and **4** (pathway b). The isopentyl side chain is shown in lilac. B) RslO9 activity assay with natural substrate **5**. Representation of three DAD chromatograms at 254 nm with the control reaction of compound **5** in black, the RslO9 assay quenched after 1 min in blue, and the same reaction quenched after 15 min in orange. The initial reaction intermediate **6** is highlighted with a green bar, rishirilide D (**3**) as the major product (without ketoreductase RslO8) with a cyan bar, and substrate **5** with a purple bar. The inset on the right side shows the saturation kinetic analysis of RslO9 for the conversion of **5** based on triplicate measurements, indicated by black squares with error bars (SD). The fit of the data is shown as a dark blue line, resulting in a *v*_max_ of 6.6 ± 0.8 min^–1^ and a *K*_M_ of 19 ± 7.7 μM (for experimental details, see the Materials and Methods section).

RslO9 and other group A FPMOs utilize a flavin adenine dinucleotide (FAD, here referred to as Fl) cofactor, which can be reduced to Fl_red_ and then react with molecular oxygen (O_2_). Typically, O_2_ forms a covalent adduct with the C4a atom of the flavin, leading to a C4a-(hydro)­peroxide (Fl_C4aOO(H)_) in group A FPMOs, allowing the incorporation of one oxygen atom into the substrate, while the other is reduced to water and released from the cofactor to regenerate oxidized flavin (Fl_ox_).^5,17–19^ Group A FPMOs are single-component enzymes capable of reducing their tightly bound FAD cofactor via NAD­(P)H in the presence of the native substrate, whose binding to the enzyme typically initiates the pivoting of the cofactor at the ribityl C(2) of the isoalloxazine ring from the inside (“IN” conformation) to the outside (“OUT” conformation) of the enzyme. Surface exposition of Fl_ox_-OUT then enables the two-electron reduction by NAD­(P)­H, which transiently interacts with these enzymes.^20^ Following reduction, the flavin cofactor flips back into the active site, where Fl_red_-IN reacts with O_2_ forming the reactive Fl_C4aOO(H)_ species that then oxygenates the nearby substrate. Although the role of RslO9 during rishirilide biosynthesis has been established, its mechanistic and structural features remain unexplored. Previously, it was proposed that RslO9 catalyzes a Baeyer–Villiger oxidation to produce **6**, without explicit experimental support. To provide insights into the underlying FPMO chemistry and late-stage tailoring of aromatic polyketides, we set out to further investigate RslO9 mechanistically and structurally, which revealed surprising enzyme functionalities and a catalytic route distinct from those of canonical BVMOs.

## Results and Discussion

### Rishirilide Stereochemistry

Upon reassessment of the formerly proposed RslO9-mediated conversion of the A-ring of **5** into a seven-membered lactone, followed by an intramolecular aldol condensation to yield **6** (Figure S1A), it became evident that the postulated reaction sequence is incompatible with the stereochemistry reported for compound **2**. Recently, the originally proposed 2*R*,3*R*,4*R* configuration of **2** ^12^ was revised to 2*S*,3*S*,4*S* based on the enantioselective total synthesis of both enantiomers and comparison to an authentic standard (Figure 1).^21^ However, the proposed RslO9-catalyzed aldol condensation step, in which a hypothetical carbanion attacks the isopentyl side chain to form a 2*S*,3*S*,4*S*-configured bridged ring system, is sterically hindered by the seven-membered lactone ring (Figure S1B). In contrast, alternative **2** configurations, in particular 2*S*,3*R*,4*S*, would be theoretically compatible with a Baeyer–Villiger oxidation-based RslO9 mechanism (Figure S1). Therefore, the stereochemical configuration of the rishirilides was first revisited, as the putative diastereomeric 2*S*,3*R*,4*S*-**2** was not completely ruled out based on previous studies. To this end, a bacterial culture was upscaled for the isolation of **2**, which was further subjected to NOESY-NMR and IR/VCD spectroscopy. NOE correlations observed between the isopentyl substituent at C4 and the C2 proton in ring A (Figures S2–S8 and Table S1) confirmed the trans relative orientation of the C2-methyl and C4-isopentyl groups. In addition, IR and VCD measurements were performed in chloroform-d with the addition of 7-azaindole to aid solubility and allow for straightforward spectral calculations (see methods section).^22,23^ Comparison of experimental and computed IR and VCD spectra confirmed the stereochemistry of **2** as 2*S*,3*S*,4*S*. The computed spectra of the alternative configuration of 2*S*,3*R*,4*S* did not match the experimental spectrum (see Figure S9, Table S2, and Supplementary Note 1 for detailed spectral analysis), therefore confirming the configuration proposed by Odagi et al.^21^

### Structural Features of RslO9

The incompatibility of the proposed BVMO functionality of RslO9 with the confirmed stereochemistry of **2** raised the question of how the enzyme instead facilitates the complex formation of the bridged ring system. To further investigate this, the enzyme structure was first elucidated by protein X-ray crystallography following the heterologous production and purification of RslO9 (Figure S10). For initial crystallization trials, an N-terminal deletion mutant RslO9PAP (PAP indicating the first three amino acids of the natural sequence which were retained; see Figure S11 for used protein sequences) was used that lacked a predicted flexible extension likely to impede crystallization. After prolonged attempts, the structures of RslO9PAP (PDB ID 9QM2) and subsequently also RslO9 wild type (PDB ID 9QM4) were obtained at resolutions of 2.4 and 2.7 Å, respectively (for details, see the Materials and Methods section and Table S3). The electron density of both structures was well-defined and allowed for unambiguous placement of the FAD (see Figure S12 and Supplementary Note 2 for an overview of FAD binding). Most of the holo-protein could be reliably modeled, with the exception of the N-terminal His-Tag and linker region of RslO9PAP, the natural N-terminal extension of RslO9WT, as well as a few highly flexible surface residues. RslO9 adopts a fold typical for group A FPMOs, also observed for the closely related and structurally characterized homologues RdmE (PDB ID: 3IHG) and GrhO5 (PDB ID: 7OUC).^7,24^ These enzymes comprise three distinct domains, that is, an FAD-binding domain (residues 1–96, 121–219, and 311–416 for RslO9), a central domain (residues 97–120 and 221–310; involved in substrate binding), as well as a C-terminal thioredoxin-like domain (residues 417–565). The thioredoxin-like domain is absent in some group A FPMO members and does not seem to directly contribute to catalysis (RslO9 lacks the two catalytic cysteine residues necessary for thioredoxin activity). For MHBH,^25^ this domain is involved in dimer formation but appears redundant in other cases, e.g., for the homologues MtmOIV, CabE, and GrhO5.^7,26,27^ In the crystal lattice of RslO9, no interactions point toward a dimer, and size-exclusion chromatography coupled with multiangle light scattering (SEC–MALS) analysis indicated a mass of 56.2 and 58.3 kDa for RslO9PAP and RslO9WT, confirming that RslO9 is also monomeric in solution (see Figures S13/S14). To gain insights into substrate binding and putative catalytic residues of RslO9, cocrystallization and soaking experiments were conducted. The labile native substrate **5** was obtained from culture supernatants of the *rslO9* knockout strain.^10^ Compound **5**, along with analogues **6** and **7** (which bound to and were converted by RslO9, *vide infra*), were employed for cocrystallization without NADPH to prevent turnover; additionally, **5** was used for soaking. Although these attempts were unsuccessful in obtaining a structure with high resolution and defined electron density in the active site, a **5**-soaked structure of RslO9 at a resolution of 3.1 Å (PDB ID 9QM3) showed a distinct shifting of a loop occluding the active site in the unsoaked protein (see Figure S15). This loop, defined by the amino acid sequence “PPTGG”, is conserved in the close homologues GrhO5 and RdmE and shifts upward in their crystal structures when complexed with their respective substrates (PDB IDs: 7OUD and 3IHG). Hence, RslO9 appears to employ a similar mechanism, and the new structure therefore likely presents the catalytic state in which the native substrate can be bound and processed.^7^ For further specifics and discussion about the RslO9 structural features and comparison to characterized homologues, see Figures S16/S17 and Supplementary Note 3.

### Docking and Molecular Simulations of RslO9 with the Natural Substrate **5**

The RslO9 structure with the open active site and presumed catalytic state now allowed for molecular docking and molecular-dynamics-supported Monte-Carlo simulations. Gratifyingly, a plausible docking position within the active site of RslO9 for native substrate **5** could be identified, in which rings A and B were positioned perpendicular to the FAD cofactor and close to its reactive N5–C4a locus (see Figure 2B). Notably, based on this docking, the most plausible site of oxidative attack is the C4 of compound **5**, with a distance of 5.0 Å to the flavin-C4a and angles that align with prior observations for FPMOs.^4^ Key interactions stabilize the binding of **5**: the C1-carbonyl oxygen of **5** forms a hydrogen bond with the amide backbone nitrogen of G339 in the “PPTGG” signature loop above the FAD, while the C5 and C10 hydroxyl groups are within the hydrogen-bonding distance of the T74 carbonyl oxygen in the loop below the FAD. On the opposite side of the FAD cofactor, the aliphatic chain of **5** is close to F269, and its three-ring core structure is surrounded by F104, F247, F258, V256, H251, and M106. The terminal ring C resides near the catalytic pocket’s boundary, adjacent to W136 and Y77, and the overall positioning of **5** relative to the FAD closely resembles the cocrystal structures of homologous enzymes RdmE and GrhO5 (PDB IDs: 3IHG, 7OUD/C). However, a notable distinction is that in 11-aklavinone hydroxylase RdmE, the substrate—a planar three-ring system with an additional ring and aliphatic substituent—is rotated 180° relative to **5**. Despite this flip, both enzymes position the substrates’ proposed sites of oxygenation directly in front of the FAD in the “IN” conformation, that is, the phenolic B-ring of aklavinone for RdmE and the C4 of compound **5** for RslO9. These distinct substrate orientations thus determine the different regioselectivities for the oxygenation reactions catalyzed by RslO9 and RdmE. It is noteworthy that the RslO9 structure and docking suggested oxygenation of **5** at C4, the same site previously proposed for the BVMO reaction. However, in contrast to type I and type II BVMOs that typically have substrates with isolated ketone functional groups,^28^ the C4a of **5** is part of a β-keto–enol­(ate) motif and thus significantly more electron-rich and less prone to be attacked by a nucleophilic anionic Fl_C4aOO_ species; furthermore, clear candidates for a catalytic base that could facilitate deprotonation of the initially formed Fl_C4aOOH_ species or an Arg residue for the stabilization of the Criegee intermediate are missing, as observed for some BVMOs.^17,28^ Overall, these stereochemical, structural, and biochemical considerations support an initial hydroxylation rather than a lactone-forming Baeyer–Villiger oxidation.

**Figure 2:**
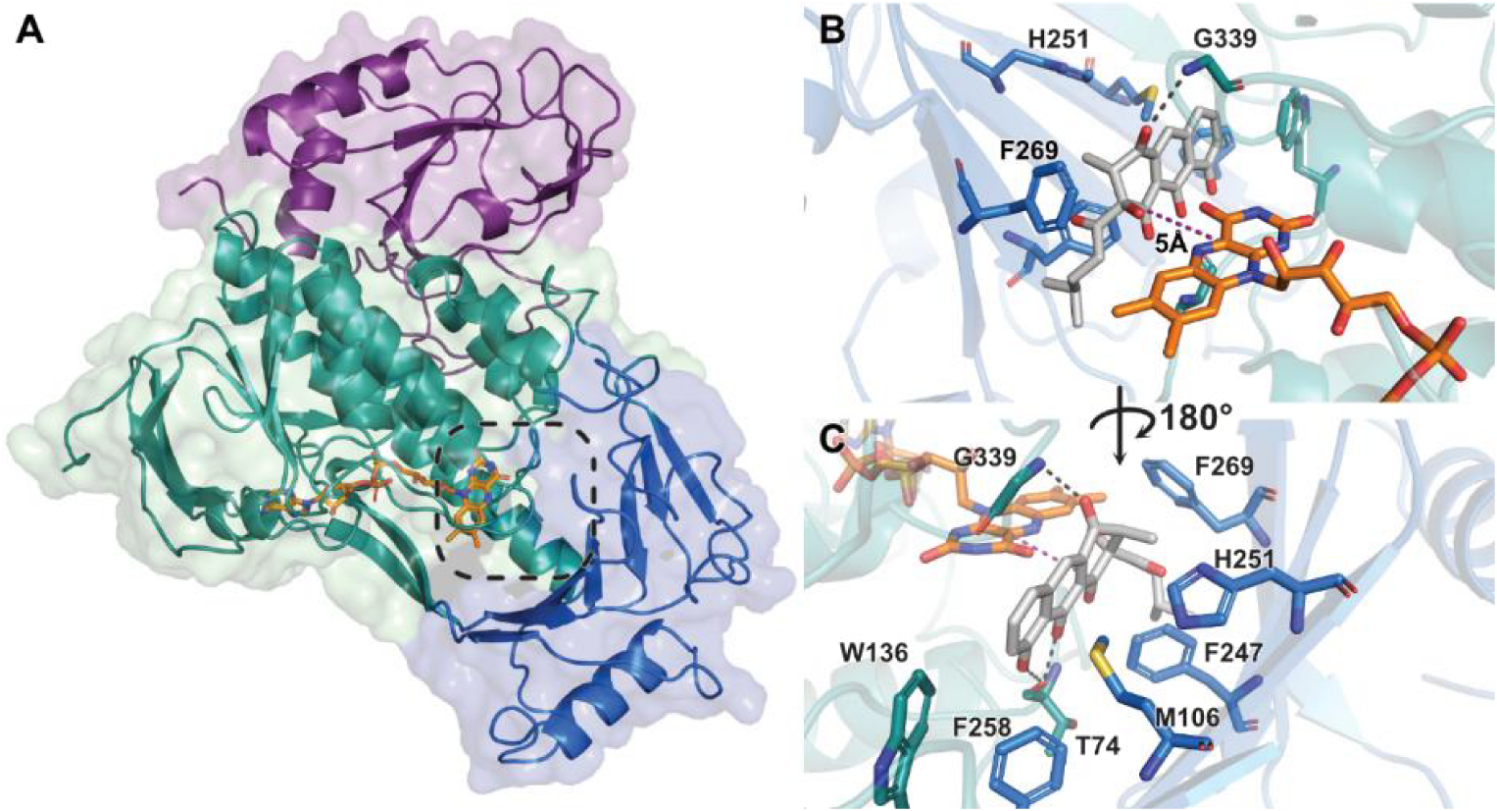
A) Domain structure representation of RslO9, with the FAD-binding domain represented in green, the middle domain shown in blue, and the C-terminal thioredoxin-like domain shown in purple. The bound FAD cofactor is shown as orange sticks. B/C) Overview of native substrate **5** modeled into the RslO9 active site. The distance between the potential oxygenation site of **5** and C4a (where the oxygenating species is formed) of the FAD cofactor is 5.0 Å and indicated by magenta dashes. Surrounding amino acids are labeled with their one-letter code, and polar interactions are shown with gray dashed lines.

### Thermal Stabilization and Substrate Promiscuity of RslO9

To further elucidate the catalytic mechanism of RslO9, thermal shift assays were first conducted to quantify the thermal stabilization of the protein in the presence of different substrate analogues. The tested compounds resembled the native substrate **5** and featured either naphthoquinone (**7**–**12**) or anthraquinone (**13**–**15**) backbones, with an increased melting temperature (*T*_m_) of RslO9 indicating ligand binding (for an overview of assays and substrates, see Table S4 and Figures S18–S29). In addition, LC–MS analyses of *in vitro* assays were performed to investigate RslO9’s substrate promiscuity and catalytic turnover of these compounds (Figure 3). The highest stabilization of RslO9 (by ∼7.5 °C) was expectedly achieved with the native substrate **5**, also showing the feasibility of this approach to study RslO9–ligand interactions. Among the substrate analogues that were tested, natural products like shikonin (**7**) or lapachol (**8**), which contained an additional alkyl chain (R_2_ in Figure 2) connected to the naphthoquinone core similar to **5**, showed higher stabilization (∼2–4 °C) compared to compounds lacking these substituents (∼0–2 °C). The docking and molecular dynamics simulations of RslO9 and **5** suggested that the naphthoquinone substituents on the eastern molecule side (substituents R1 and R2 in Figure 3) are surrounded by apolar amino acid side chains of F269 and V256, facilitating stabilizing hydrophobic interactions. In contrast, the polar R_2_ hydroxyl group of **12** apparently resulted in a small thermal destabilization of RslO9. Interestingly, plumbagin (**9**), featuring a hydroxyl group in position R_3_ and a methyl group as substituent R_1_ but lacking an alkyl chain at R_2_, stabilized RslO9 to a degree comparable to **8**. Likely, substrate binding therefore also depends on the hydrogen bond between the T74 backbone carbonyl and the phenolic hydroxyl group of the substrate. Consequently, the absence of an aliphatic substituent at position R_2_ for **9** appears to be compensated by the hydroxyl group. This is supported by the substantially lower thermal stabilization of RslO9 by **10**, which lacks this hydroxyl group but is otherwise identical to **9**. Interestingly, anthraquinone scaffolds, which are often observed in biosynthetic pathways of other aromatic polyketides, show no to moderate stabilization of the enzyme (∼0–1.5 °C). Moreover, these compounds were not converted in the *in vitro* assays in contrast to most naphthoquinones (Figure 3), suggesting specificity toward compounds with a *para*-quinone substructure flanked by only one aromatic ring. Moreover, naphthoquinones with an additional alkyl group, e.g., the natural product lapachol (**8**), were converted almost completely under standard reaction conditions, in contrast to their nonsubstituted counterparts. Overall, this underscores the facilitating effect of the alkyl substituent, consistent with the observed higher thermal stabilization of RslO9 by these compounds.

**Figure 3:**
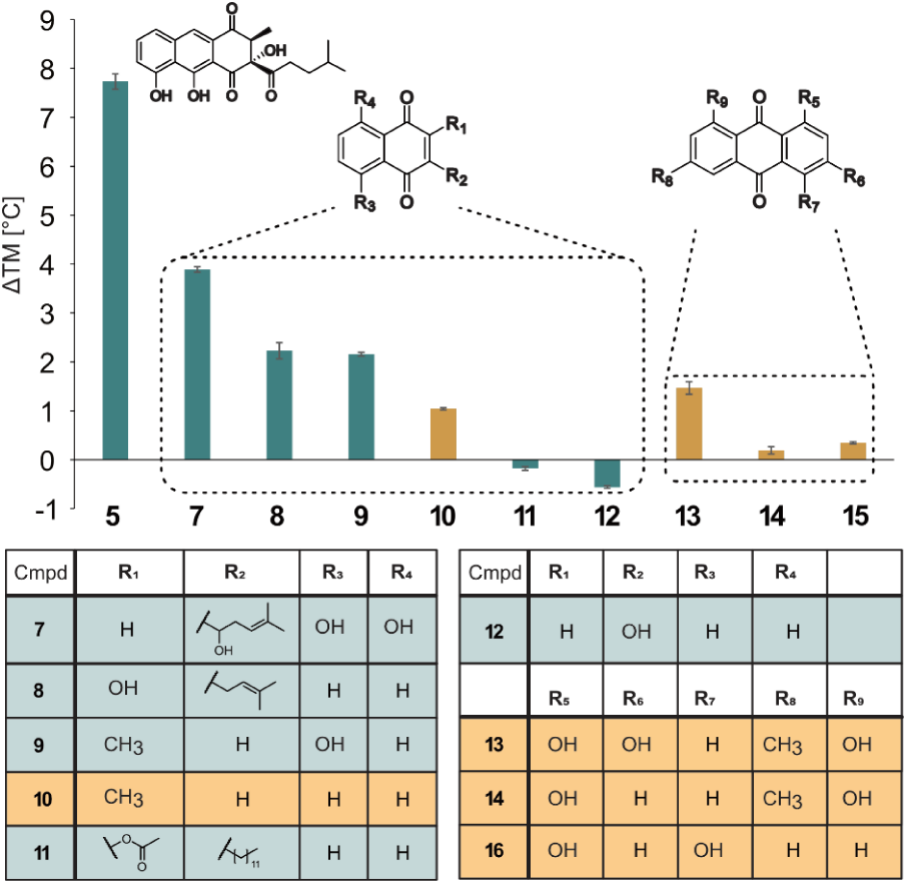
Representation of the relative stabilization of RslO9 in °C (*y*-axis) by different ligands (*x*-axis) compared to the solvent control. Green bars indicate conversion of the substrates by RslO9, while compounds with orange bars were not accepted. Residues (R) of the naphthoquinone and anthraquinone scaffolds for the presented ligands are shown in the table below (see Supplementary Table S4 for a comprehensive overview of the compounds).

### RslO9 Catalyzes Formation of the Distinct “Hooker Intermediate”

To gain insight into the catalytic mechanism of RslO9, the efficient conversion of **8** into a single product (**16**) was selected for further investigation. Accordingly, an upscaled and optimized enzyme assay allowed the isolation and structural elucidation of **16** by comprehensive NMR and HRMS/MS analysis. To our surprise, **16** was identified as the characteristic “Hooker intermediate” (compare Figures S30–S37 and Table S5) from the one-pot Hooker oxidation first described in 1936.^29,30^ This reaction under mild conditions comprises the conversion of **7** via **16** into norlapachol (**17**) and involves the degradation of the prenyl side chain by one carbon unit, accompanied by a reversal in the regiochemistry of the 2-alkyl group and the 3-hydroxy substituent of **8**. More recently, the formation of key intermediate **16** from **8** in the first half of the Hooker oxidation was shown to involve an epoxidation, epoxide opening, and subsequent benzilic acid rearrangement.^31^ To date, however, no enzyme has been reported to facilitate a similar chemistry. Assuming that RslO9 catalysis proceeds by an analogous mechanism, initial flavin-mediated epoxidation or hydroxylation should take place. To further investigate this, we attempted to trap the expected transient reactive intermediate (**18**) arising from the epoxide ring-opening reaction in the initial oxidation step. To this end, *o*-phenylenediamine (OPD) was added to the enzymatic assays, which indeed allowed the capture of **18** and formation of the corresponding phenazine derivative **19** (previously reported for the Hooker oxidation^31^), as verified by HRMS analysis (Figure 4A, Figures S38–S46). Importantly, the formation of intermediate **18** thus underscores that the RslO9 reaction does not involve Baeyer–Villiger oxidation. To further validate the “Hooker-like” oxygenation mechanism for RslO9, ^18^O-labeling experiments were performed next. Enzyme reactions conducted in an ^18^O_2_ atmosphere or with H_2_^18^O supplementation each showed the incorporation of one ^18^O into **16**, as confirmed by LCMS and MS/MS analysis (Figures S39–S41), corroborating both the monooxygenase activity of RslO9 and the water addition during the benzilic acid rearrangement. Notably, in enzyme assays conducted under ^18^O_2_ atmosphere and in the presence of OPD, the ^18^O label was retained in derivative **19** but not observed for the H_2_^18^O experiment, in line with the proposed mechanism and oxygenation site (Figures S42–S43). To explore the oxygenation mechanism further, compound **7** was O-methylated with iodomethane to yield **20** (see Figures S47–S52). However, while derivative **20** could be oxygenated under Hooker oxidation conditions (H_2_O_2_, Na_2_CO_3_, room temperature) to a stable epoxy-quinone,^31^ it was not enzymatically converted by RslO9. Based on these findings, the conversion of **8** into **16** by RslO9 is proposed as shown in Figure 4A.

**Figure 4:**
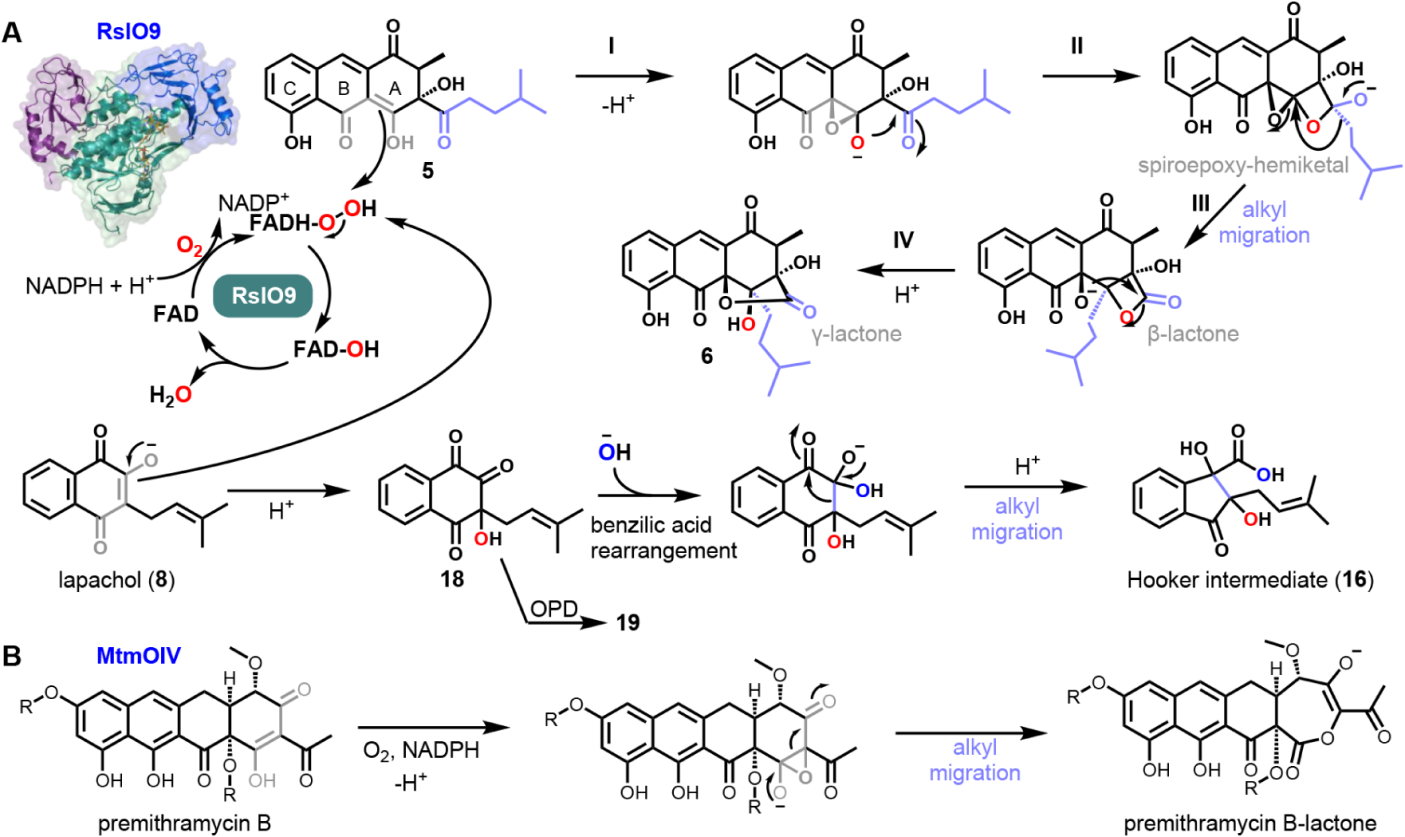
A) Proposed catalytic mechanism of RslO9 for the oxidative rearrangement of the native substrate **5** (top; tautomer is shown) and substrate analogue **8** (bottom) utilizing a flavin hydroperoxide as the oxygenating species. Oxygen atoms derived from O_2_ and H_2_O based on isotope-labeling experiments are labeled in red and blue, respectively. The side chain is shown in lilac. See text for a description of individual steps. B) Possible MtmOIV mechanism analogous to RslO9, leading to the formation of premithramycin B-lactone. R = sugar residues. All shown substrates (**5**, **8**, and premithramycin B) feature a β-keto–enol­(ate) motif (in gray) undergoing oxygenation.

### RslO9 Facilitates a Distinct Alkyl Migration during Rishirilide Biosynthesis

The obtained results from studying the conversion of **8** into **16** provide important insights into the catalytic features of RslO9. These observed enzyme functionalities, the chemical properties of native substrate **5**, and stereochemical considerations now allow us to redefine the mechanism for **6** biosynthesis and strongly suggest that the initial oxygenation involves an electrophilic Fl_C4aOOH_ species (rather than a deprotonated, nucleophilic flavin peroxide). Moreover, the observed docking pose implies that attack by the Fl_C4aOOH_ is most likely to occur at C4 (rather than C4a) of the native substrate **5** (Figure 2B, Figure 5 and Figure S53). Hence, we propose that RslO9 oxygenates natural substrate **5** via a gem-diol-forming electrophilic addition to the enolate double bond, followed by epoxide formation under quenching of the transient carbocation (Figure 5). This mechanism requires the β-keto–enol­(ate) tautomer of native substrate **5**, which is likely favored under physiological conditions due to stabilization by intramolecular H-bonding of the alternate ring hydroxyl and keto groups. Notably, the epoxide is proposed to form via nucleophilic attack by the oxygen of the original C4-hydroxyl group (rather than the newly introduced one) on the adjacent carbocation, accounting for both the observed docking pose and the stereochemistry of product **6** (step I, Figure 4A and Figure 5). The proposed mechanism involving the tertiary carbocation intermediate seems likely, given the substantial stabilization of this intermediate by delocalization of the positive charge through rings B/C and the adjacent ketone.^32^ This is comparable to the flavin-dependent indole monooxygenase, where substrate epoxidation is proposed to proceed via a similar stepwise mechanism and a carbocation intermediate based on quantum-chemical modeling.^33^ It is noteworthy that gem-diol formation apparently does not occur during the initial hydroxylation of the non-native substrate **8** by RslO9 based on the ^18^O-labeling experiments, presumably due to the increased acidity of the hydroxyl group (caused by the additional adjacent electron-withdrawing ketone), making hydroxylation of the tertiary carbon more likely that affords **18** (Figure 4A).

**Figure 5:**
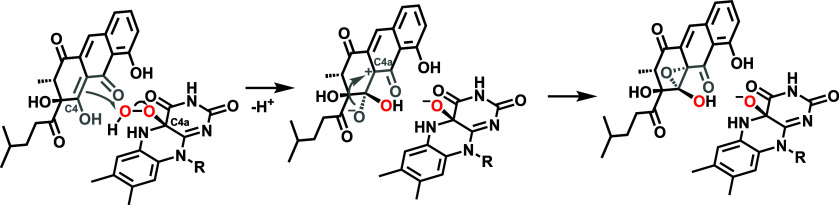
Proposed RslO9-catalyzed Fl_C4aOOH_-dependent hydroxylation at C4 of **5** is based on the docking position (Figure 2B). Note that the docking analysis revealed that the Fl_C4aOOH_ species is positioned with significantly shorter distances and more favorable angles relative to the target oxygenation site C4 of compound **5** compared to C4a (see Figure S53).

Epoxide formation could then be followed by an attack of the introduced hydroxyl group (that is possibly activated by flavin-C4a-alkoxide-mediated deprotonation) on the nearby ketone of the side chain to form a transient spiroepoxy–hemiketal intermediate (step II). This would enable the subsequent alkyl migration (similar to the benzilic acid rearrangement observed for **18**) of the side chain under epoxide opening to afford transient alkoxide and β-lactone species (step III). The β-lactone is subsequently opened by the attack of the alkoxide under concomitant formation of the final γ-lactone ring of product 2*S*3*S*4*S*-**6** (step IV). As a common denominator for catalysis by RslO9, the formation of compounds **16** and **6** via alkyl migration likely involves stabilization of the negative charges developing in the transition states. It is noteworthy that both prior ^13^C-labeling^34^ as well as ^18^O-labeling studies^10^ (showing only ^18^O incorporation from ^18^O_2_ but not H_2_^18^O for the conversion of **5** into product **6** by RslO9) are fully consistent with the revised mechanism. Moreover, a phylogenetic analysis aligns with the proposal that RslO9 functions as a hydroxylase/epoxidase, as the vast majority and also the most closely related characterized group A FPMOs are proposed to similarly employ electrophilic Fl_C4aOOH_ species, e.g., GrhO5,^7^ RdmE,^24^ or MbnF^35^ (Figures S16–S17).

### Investigation of His251 as a Putative Catalytic Residue of RslO9

Among the multitude of hydrophobic residues (F104, F247, F258, and F269) lining the binding pocket in the RslO9 active site, H251 (conserved in several predicted RslO9 functional homologues, see Figure S54) stands out as a polar residue potentially involved in catalysis. Structural alignment with RdmE shows that H251 is positioned similarly to Y224 of RdmE, which was proposed as a key catalytic residue.^24^ Accordingly, site-directed mutagenesis studies of RslO9 were performed by exchanging histidine with asparagine (H251N) and alanine (H251A), respectively. Compared to the wild-type enzyme, the variants showed a slight reduction in activity to around 70–75% at pH 7.5 with the model substrate **8**, and no alternative products were observed, ruling out H251 as a *bona fide* catalytic residue. However, the histidine side chain may play a more critical role at lower pH values, potentially accelerating the alkyl migration (or benzilic acid rearrangement in the case of **8**) by stabilizing the developing negative charge on the migrating alkyl group in the transition state and/or promoting the final protonation step (step IV, Figure 4). Indeed, when the assay pH was reduced from 7.5 to 6.5, the relative activity of the enzyme mutants was further reduced to 25–30%, in contrast to that of the wild-type enzyme, which only showed a small decrease in activity (Figure S55). Notably, the docking results with the native substrate **5** suggest that H251 is positioned approximately 8–9 Å from ring A, indicating that it is unlikely to directly participate in substrate (de)­protonation, assuming that no significant shifts in substrate binding occur during catalysis. The molecular dynamics simulation showed multiple water molecules above the substrate, and it is thus conceivable that H251 acts indirectly through those water molecules by stabilizing the anionic intermediates during catalysis. Further studies are required to elucidate the exact role of H251 and other active site residues in RslO9.

### RslO9-Catalyzed Oxygenation and Alkyl Migration May Serve as a Blueprint for Related FPMO Mechanisms

These findings for RslO9 catalysis prompted us to revisit related group A FPMOs previously proposed to act as BVMOs, in particular the well-investigated MtmOIV from the biosynthesis of the aromatic polyketide mithramycin, a formerly clinically used anticancer agent.^26,36^ This enzyme was classified as a prototype of the heterogeneous atypical BVMOs, belonging to group A FPMOs with Rossmann (GR-2) fold as opposed to canonical type I (group B FPMOs with Rossmann (FMO) fold) and type II BVMOs (group C two-component FPMOs with Tim-barrel (luciferase) fold).^5,17,18,28^ Notably, neither a specific catalytic base for generating the anionic flavin peroxide nor the proposed Criegee intermediate of the Baeyer–Villiger oxidation has yet been experimentally confirmed for atypical BVMOs such as MtmOIV to our knowledge. Moreover, not only are the protein structures of RslO9 and MtmOIV similar, but also their substrates and structural motifs (i.e., β-keto–enol (ate)­s) that undergo oxidation. We thus speculate that both enzymes employ similar oxygenation chemistry to facilitate C–C bond cleavage and alkyl migration, as shown in Figure 4B. Accordingly, MtmOIV could hydroxylate the enolate double bond of premithramycin B, followed by epoxide formation, which could rearrange to premithramycin B-lactone. Analogous mechanisms can be envisaged for additional putative atypical BVMOs where similar electron-rich oxygenation sites would be unfavorable for a Baeyer–Villiger oxidation, e.g., PxaB involved in the biosynthesis of bacterial pyrrolizidine alkaloids.^37^

## Conclusions

In this work, we characterized the key enzyme RslO9 catalyzing a complex backbone rearrangement that is central to rishirilide biosynthesis. This reaction adds to other FPMO-catalyzed rearrangements and tailoring reactions reported from aromatic polyketide biosynthesis, e.g., oxidative ring cleavage and xanthone formation by XanO4 ^38^ or FlsO1,^39^ MtmOIV-mediated lactone formation in mithramycin biosynthesis,^9,26,36^ Favorskii rearrangement in enterocin biosynthesis,^40–42^ or GrhO5- and GrhO6-catalyzed aromatic hydroxylations, decarboxylations, and spiroketal formation in griseorhodin A biosynthesis.^7,8^ Extensive probing of RslO9’s substrate scope revealed a preference for compounds harboring a terminal *para*-naphthoquinone moiety with a facilitating effect of alkyl substituents. Further structural and mechanistic investigations with substrate analogue **8** provided evidence for a surprising reaction resembling the first half of the Hooker oxidation, which involves an initial monooxygenation followed by a benzilic acid rearrangement. For native substrate **5**, a similar alkyl migration is proposed, fully consistent with the stereochemistry of the rishirilides—an enzyme functionality that overturns the previously proposed BVMO chemistry and suggests that mechanisms of other apparent (atypical) BVMOs such as MtmOIV require further scrutiny in the future.

## Materials and Methods

### Materials

All chemicals and reagents used during this study were obtained from Sigma–Aldrich (St. Louis, MO, USA), Alfa Aesar (Haverhill, MA, USA), Biomol (Hamburg, Germany), Carl Roth (Karlsruhe, Germany), and Fisher Scientific (Hampton, NH, USA). Materials used for molecular cloning were purchased from New England Biolabs (NEB), Thermo Fisher Scientific, or Sigma–Aldrich. Plasmid DNA sequencing and synthesis of primers were carried out by Microsynth (Balgach, Switzerland). Protein purification equipment (Ni–NTA columns, maltose-binding protein (MBP)-trap columns, gel filtration columns) was purchased from Cytiva (Marlborough, MA, USA). Proteins were concentrated in centrifugal devices from Thermo Fisher Scientific (New York, USA). Agarose gel electrophoresis and SDS-PAGE were carried out using respective equipment from Cleaver Scientific (Rugby, UK).

### Multiple Sequence Alignment and Phylogenetic Tree Construction

Multiple sequence alignment of RslO9 and the closest described homologues was performed with the MUSCLE algorithm within the MEGA11 software. Multiple sequence alignment was manually adjusted based on the structural alignment of the crystal structures of the respective enzymes. The phylogenetic tree was constructed with the maximum likelihood method and validated by 1000 bootstrap iterations. Visualizations of the tree were generated with the iTOL tool and manually adjusted.

### Cloning

The *RslO9* gene was cloned from the genome of *Streptomyces bottropensis* as described previously.^10^ Additionally, the N-terminal deletion variant RslO9PAP and the full length RslO9 (RslO9WT) were produced and cloned via sequence- and ligation-independent cloning into the pNIC-Bas4 and pNIC-MBP vectors, respectively (see Figure S11 for all protein sequences used in this study).

### Protein Production and Purification

RslO9PAP with an N-terminal histidine tag and RslO9WT with an N-terminal histidine and MBP tag were produced in *E. coli* BL21 (DE3). Cells were grown in TB medium supplemented with 0.5 g/L of glucose and 2 mM MgSO_4_ to an OD600 of 0.5–0.7 at 37 °C. The cultures were cooled to 18 °C before induction with 0.1 mM IPTG, and cultivation was continued at 18 °C. Cells were harvested after around 18 h and stored at −20 °C until further use. For enzyme purification, the cells were resuspended in lysis buffer (50 mM HEPES, 400 mM NaCl, 10 mM imidazole, 10% (v/v) glycerol, pH 7.5) and lysed by sonication. The clarified lysate was loaded onto a 5 mL Ni-NTA column and washed before the target enzyme was eluted with elution buffer (25 mM HEPES, 200 mM NaCl, 500 mM imidazole, pH 7.5). For MBP-tag-containing constructs, immobilized metal chelate affinity chromatography (IMAC) elution fractions were directly loaded onto a 5 mL MBP-trap column equilibrated with binding buffer (25 mM HEPES, 200 mM NaCl, pH 7.5), while His-tag constructs were desalted into 20 mM Tris, 100 mM NaCl, pH 7.4 buffer with 4 HiTrap desalting columns. MBP-tagged RslO9 was eluted from the column with binding buffer supplemented with 10 mM maltose. For enzymes used for crystallization, the pNIC constructs were cleaved with TEV protease overnight at 30 °C. The cleaved proteins were separated from TEV and impurities by loading onto a Ni-NTA column and collecting the flow-through with subsequent purification by size exclusion chromatography on a 16/600 200 pg column (20 mM Tris, 100 mM NaCl, pH 7.5).

### Site-Directed Mutagenesis

The chosen active site variants of RslO9 were produced by the QuickChange procedure for the pNIC-MBP-RslO9WT construct. Partially overlapping mutagenic primers (see Table S6 for oligonucleotide sequences) were used for amplification with the Q5 DNA polymerase before DpnI digestion and transformation into *E. coli* XL1-Blue. Correct introduction of the mutations was validated by sequencing. All RslO9 variants were produced as described above for the WT enzyme. The proper folding of the enzyme variants was assessed by SEC before the activity assays (see Figure S56).

### Production and Purification of the Natural Substrate RSHO9 (**5**)

The natural substrate RSHO9 was produced from the *Streptomyces bottropensis* RslO9 knockout strain as described previously.^10^ In short, the respective strain was grown in MYMv medium for 5 days at 28 °C. After harvesting and pH adjustment to 6, the supernatant was extracted with ethyl acetate. Dried extracts were dissolved in MeOH and applied onto a solid-phase extraction column [Oasis HLB 35 cm^3^ (6 g) LP Extraction Cartridge, Waters GmbH, Eschborn, Germany] and eluted with increasing methanol concentration. Fractions containing RSHO9 were further purified by semipreparative HPLC on a Waters SunFire C18 OPD Prep Column 100 Å, 5 μm (with a precolumn of the same material) with water and acetonitrile (MeCN) as the mobile phase and 0.1% formic acid as the modifier.

### Production and Purification of Rishirilide B (**2**)

Rishirilide B (**2**) as the main product of the rishirilide biosynthesis pathway was produced and purified from a heterologous producer strain, similar to a previously described protocol.^27^ The heterologous producer *S. albus*::cos4 was cultivated in 50 mL of TSB medium (containing apramycin) in a 250 mL baffled flask for 24 h. This preculture was used for 2% (v/v) inoculation of 500 mL MYMv medium (containing apramycin) in 2 L baffled flasks and further incubated for 5 days at 28 °C. After harvesting the cultures, the supernatant was adjusted to pH 6.0 and extracted three times with an equal volume of EtOAc. The combined organic extract was evaporated to dryness, and **2** was purified by semipreparative HPLC on a Waters SunFire C18 OPD Prep Column 100 Å, 5 μm (with a precolumn of the same material) with MeCN and water (both containing 0.1% formic acid as a modifier) as the mobile phase under 38% MeCN isocratic conditions.

### Thermal Shift Assays via nanoDSF

For thermal shift assays with the natural substrate or alternative ligands, RslO9WT without any tags was diluted to a 2 mg/mL concentration. Thermal denaturation was tested on the Nanotemper Prometheus NT.48 (Nanotemper Technology), starting from 20 °C with a temperature gradient of 1 °C/min. The ligands were added to the nanoDSF buffer (20 mM HEPES, 100 mM NaCl, pH 7.5) to a final concentration of 400 μM. Assays with alternative ligands contained 2% (v/v) DMSO and 0.4% (w/v) hydroxypropyl-β-cyclodextrin for solubility. All measurements were performed in triplicate, and controls were performed with the same buffer and additives as the compound measurements.

### SEC–MALS Measurements

The RslO9PAP variant and RslO9WT without any tags were applied at 2 mg/mL for SEC–MALS analysis on an Agilent 1260 HPLC with a Superdex 200 column and Wyatt Heleos II 8+ (8-angle) MALS detector, as well as a Wyatt Optilab rEX differential refractive index detector, equilibrated in 20 mM HEPES, 100 mM NaCl, pH 7.5.

### Crystallization of RslO9

RslO9PAP (10 mg/mL in 20 mM Tris, 50 mM NaCl, pH 7.4) from the pET28a construct was crystallized in a sitting-drop vapor diffusion setup by mixing 200–400 nL of enzyme solution with 400–200 nL of screening solution. Plate-shaped crystals formed after multiple days in 300 mM ammonium formate, 100 mM HEPES, and 20% (w/v) Sokalan CP5 at pH 7.0. The RslO9WT without tags from the pNIC-MBP vector (18.6 mg/mL in 20 mM Tris, 100 mM NaCl, pH 7.4) was crystallized in the same sitting-drop vapor diffusion setup but with 100 mM HEPES ranging from pH 6.25 to 7.25 with 39.4–48.8% (w/v) PAA 2100 as the precipitant. Crystals grew in a similar shape as before over the course of 5–6 days at room temperature. Crystals were cryoprotected by the addition of 15% (v/v) final concentration of glycerol and frozen in liquid nitrogen until measurement.

### Protein Crystal Data Collection, Processing, and Visualization

Diffraction data were collected at Diamond Light Source (Didcot, UK) beamline i03 and Swiss Light Source (Villigen, Switzerland) beamline X06SA. Diffraction data were processed using XDS^43^ and DIALS.^44^ The structure was solved by molecular replacement in Phaser^45^ using a model of RslO9PAP generated by AlphaFold 3.^46^ Successive rounds of model building and refinement were performed using Coot^47^ and phenix.refine or Refmac5.^48^ Images of the structure models were generated in PyMol (version 2.5.0).

### *In Vitro* Activity Assays

For the conversion tests, 20 μM wild-type RslO9 was incubated with 250 μM of different substrate analogues, 4 mM NADPH, 0.8% (w/v) hydroxypropyl-β-cyclodextrin, and 250 U/mL catalase at 30 °C and 900 rpm. After 2 h, the reactions were quenched by extraction with 300 μL of ethyl acetate with 1% (v/v) formic acid. For the conversion of 100 μM natural substrate **5**, up to 15 min reaction times were used. Extracts were evaporated and resuspended in DMSO for analysis by LC/MS (as described below). Samples were separated on a Waters Sunfire C18 3.5 μM column with water and MeCN with 0.1% formic acid as the mobile phase. The applied gradient was 5–100% MeCN with a higher starting concentration for more hydrophobic molecules. The activity assays of WT in comparison to RslO9 variants were performed under the same conditions but with only 5 μM enzyme over a time frame of 4 h and bis-tris-propane as a buffer system.

### Steady-State Kinetics

For the saturation kinetic analysis of RslO9 with natural substrate **5**, a photometric assay based on the decrease of the absorption maximum of **5** at 420 nm was used. Reactions were performed with varying amounts of substrate (up to 100 μM) in 100 mM Tris-HCl, pH 7.5, and final concentrations of 1% (v/v) DMSO, 2 mM NADPH, and 0.25–1 μM RslO9 at 30 °C in a microplate spectrophotometer (Thermo Scientific, Multiskan Go) in a total volume of 100 μL. A standard curve of **5** ranging from 12.5 to 200 μM was used to quantify the substrate decrease over time. The linear part of the decrease in absorption over 160 s was used to determine the initial reaction rates, which were measured in triplicates and then fitted with the classical Michaelis–Menten equation in OriginPro.

### Lapachol (**8**) Scale-Up Reaction

For the scale-up of lapachol (**8**), 5 mg was dissolved in 84 mL of 50 mM HEPES buffer, pH 7.5, containing 3% (v/v) DMSO, 1 mM NADP^+^, 0.5 U/mL GDH, 20 mM glucose-6-phosphate, 250 U/mL catalase, and 5 μM RslO9WT. The reaction was incubated in 4 equal portions in 250 mL Erlenmeyer flasks at 30 °C for 4 h under moderate shaking. The reaction mixture was extracted twice with EtOAc + 0.5% (v/v) trifluoroacetic acid. The organic phase was washed with water twice and dried with anhydrous magnesium sulfate before filtration and evaporation. The obtained product was lyophilized and dissolved in deuterated DMSO for NMR analysis.

### LC–MS Analysis

For standard measurements of assays or extracts during the purification of RSHO9, LC–MS measurements were performed on a Shimadzu LCMS-8030 Triple Quad Mass Spectrometer. Samples were analyzed on a Waters SunFire C18 column (150 mm × 3 mm ID, 3.5 μm, Waters) equipped with a guard column (10 mm × 3 mm ID). Elution proceeded with 5% MeCN in water with 0.1% (v/v) formic acid for the first 2 min and a following gradient up to 100% MeCN with 0.1% (v/v) formic acid over 20 min. After that, the column was washed at 100% MeCN and equilibrated to the starting condition for 5 min each. During the measurement, UV/vis absorption was monitored from 190 to 800 nm, and the samples were analyzed by MS in positive and negative mode with a capillary voltage of 3 kV, 250 °C DL temperature, 400 °C heat block temperature, and 3 L/min nebulizing gas flow.

### UPLC–HRMS Measurements

High-resolution mass spectrometry was performed on an Agilent UPLC system (Agilent Technologies) with an Agilent 1290 Binary Pump G4220A, Agilent 1290 Infinity Autosampler G4226A, Agilent 1290 Infinity Thermostat G1130B, Agilent 1290 Thermostatted Column Compartment G1316C, and Agilent 1290 Infinity Diode Array Detector G4212A. The connected mass detector was a Q-Exactive HF mass spectrometer (Thermo Scientific). The UPLC was equipped with an ACQUITY UPLC BEH C18 column, 130Å, 1.7 μm, 2.1 mm × 150 mm (Waters). The applied method was a gradient elution with water and MeCN, each supplemented with 0.1% formic acid, starting with 5% MeCN for the first 0.5 min, followed by a gradient up to 100% MeCN at 15.5 min, a wash at 100% MeCN up to 17.30 min, and re-equilibration to 5% MeCN for 3 min.

### NMR Measurements

The lapachol reaction product (**16**) was dissolved in 375 μL of deuterated DMSO and transferred to a 5 mm NMR tube. Rishirilide B (**2**) was dissolved in 125 μL of deuterated DMSO for measurement in a 3 mm NMR tube. The spectra were recorded on a Bruker Avance III NMR spectrometer operating at 500.13 and 125.77 MHz for ^1^H and ^13^C nuclei, respectively. ^1^H, ^13^C, HSQC, HMBC, and COSY spectra were recorded at 23 °C on a BBO probe. For comparison, the lapachol (**8**) educt was measured on the same instrument under the same conditions.

### Absolute Configuration Determination of **2** by VCD Spectroscopy

#### Experimental Details

The IR and VCD spectra were recorded on a Bruker Vertex 70/PMA 50 VCD spectrometer at 4 cm^–1^ spectral resolution by accumulating 32 scans for the IR and ∼60000 scans (14 h accumulation time) for VCD. As the solubility of **2** in MeCN and chloroform was poor, it was mixed in a 1:1 ratio with 7-azaindole, which acts as a solubilizer for carboxylic acids and simplifies the later computational analysis.^22^ Hence, 2.2 mg (6 μmol) of the sample was mixed with 0.7 mg (6 μmol) of 7-azaindole in 50 μL of chloroform-d, and the IR and VCD spectra of the mixture were recorded at a final concentration of 120 mM using a BaF_2_ IR cell with a 100 μm optical path length. Baseline correction of the VCD spectra was performed by subtraction of the spectra of the solvent recorded under identical conditions.

#### Computational Details

Deriving the absolute configuration from the experimental spectra requires the computation of the IR and VCD spectra. Therefore, a conformational sampling was carried out based on a systematic search algorithm at the force-field level (MMFF).^49^ All so-obtained conformers were subjected to further geometry optimizations at the B3LYP/6–311++G­(2d,2p)/IEFPCM­(chloroform) level of theory using Gaussian 16.^50^ To the COOH moiety of the 20 lowest-energy structures was then manually added 7-azaindole and the 1:1 complexes were again geometry optimized. For the final comparison with the experiment, the IR and VCD spectra were simulated from the single-conformer spectra using the Δ*E*_ZPC_-based Boltzmann weights and by assigning a Lorentzian band shape with a half-width at half-height of 6 cm^–1^ to the computed dipole and rotational strength. To account for effects not captured in the harmonic approximation accounted for in the spectra calculations, the frequency axis was uniformly scaled by 0.98.

### Molecular Modeling

The most likely protein–ligand binding configuration was determined using a novel protocol that combines flexible docking with molecular dynamics (MD)-supported Monte Carlo (MC) simulations.

First, flexible docking of substrate **5** into the RslO9 crystal structure was performed using Smina.^51^ The side chains for binding site residues T95, F125, M127, W157, V243, H272, F279, F290, and T359 were kept flexible throughout the docking run. The top 25 docking poses were retained as input for a novel procedure designed to identify the most probable binding pose using molecular mechanics-based evaluations, which are more accurate than classical scoring functions.

For each initial pose, ten short MD simulations of 10 ps each were performed. The first 8 ps were used for unrestrained local sampling, while during the final 2 ps, both the protein and ligand were restrained. The restrained segment allowed computation of the interaction energy with averaging over local solvent configurations, incorporating explicit solvation effects while reducing variance in the energetic contribution for pose evaluation. All ten configurations from each 10 ps block, along with their averaged energies, were stored in a replay buffer.

Subsequently, 1000 MC steps were executed, each consisting of the following sequence: 1. An 8 ps MD simulation initiated from a structure randomly selected from the replay buffer. 2. A 2 ps restrained MD simulation to compute the averaged energy of the final state over alternative solvent configurations. The resulting structure and average energy were then added to the replay buffer.

After completion of the MC simulations, all states stored in the replay buffer were clustered using self-tuning spectral clustering.^52^ The RMSD matrix between all states was first converted into an affinity matrix using local scaling based on the ten nearest neighbors. Eigen decomposition of this affinity matrix was then performed, and the optimal number of clusters (up to 25) was determined by the largest eigenvalue gap. Spectral clustering was repeated 25 times by using these candidate cluster numbers, and the optimal clustering was selected according to the maximum silhouette score.

Finally, the probability of each pose (cluster) was estimated from its relative occupancy, yielding a statistically grounded assessment of the most likely binding configuration.

MD simulation was performed using OpenMM^53^ in an NPT ensemble using AMBER99SB-ILDN^54^ and Espaloma-0.3.2 ^55^ force fields for the protein and ligand/cofactor, respectively. Periodic boundary conditions were applied to the protein–ligand system in a cubic water box with an 8 Å water buffer around the protein. Electrostatic forces were treated using particle-mesh Ewald summation with a short-range cutoff of 10 Å.

## Supplementary Material



## Notes

NMR and HRMS/MS data of analyzed compounds, IR and VCD spectra for the analysis of rishirilide B (**2**), data collection and statistics of protein crystallography, additional information and figures of bioinformatic analyses, assay chromatograms and ^18^O-labeling experiment results, information of cloning and analysis of RslO9 variants (PDF)
