# Supplementary material for: A Hooker Oxygenase Archetype in Polyketide Biosynthesis Challenging the Baeyer–Villiger Monooxygenase Paradigm
Source: J Am Chem Soc. 2026 Jan 26;148(5):5722–33. doi: 10.1021/jacs.5c21759 (PMC12903851; doi:10.1021/jacs.5c21759)
Supplement: Supplementary file 1 [file ja5c21759_si_001.pdf]

Supplementary Information

A Hooker Oxygenase Archetype in Polyketide Biosynthesis Challenging the Baeyer–Villiger Monooxygenase Paradigm

Heiner G. Weddeling[1], Sven T. Sowa[1], Elena Bialas[2], Sven Reese[3], Christian Merten[3], Markus Lill[4], Andreas Bechthold[2], Robin Teufel[1*]

[1] Pharmaceutical Biology, Department of Pharmaceutical Sciences, University of Basel, Klingelbergstrasse 50, 4056 Basel (Switzerland).
[2] Department of Pharmaceutical Biology and Biotechnology, Institute of Pharmaceutical Sciences, Albert-Ludwigs-Universität Freiburg, 79104 Freiburg (Germany).
[3] Fakultät für Chemie und Biochemie, Organische Chemie II, Universitätsstraße 150, 44801 Bochum (Germany).
[4] Computational Pharmacy, Department of Pharmaceutical Sciences, University of Basel, Klingelbergstrasse 50, 4056 Basel (Switzerland).
*E-mail: robin.teufel@unibas.ch

SUPPLEMENTARY INFORMATION 1
FIGURE S1: PREVIOUSLY REPORTED RSLO9 MECHANISM 4
FIGURE S2: HMBC, COSY AND KEY NOESY REPRESENTATION OF 2 5
TABLE S1: NMR SPECTROSCOPIC DATA FOR RISHIRILIDE B (2) ISOLATED IN THIS STUDY COMPARED TO LITERATURE VALUES.......5
FIGURE S3: ^{1}H NMR SPECTRUM OF RISHIRILIDE B (2) (DMSO-D$_6$, 500 MHZ). 6
FIGURE S4: ^{13}C NMR SPECTRUM OF RISHIRILIDE B (2) (DMSO-D$_6$, 125 MHZ). 7
FIGURE S5: COSY NMR SPECTRUM OF RISHIRILIDE B (2) (DMSO-D$_6$, 500 MHZ). 8
FIGURE S6: HSQC NMR SPECTRUM RISHIRILIDE B (2) (DMSO-D$_6$, 500 MHZ). 9
FIGURE S7: HMBC NMR SPECTRUM OF RISHIRILIDE B (2) (DMSO-D6, 500 MHZ). 10
FIGURE S8: NOESY NMR SPECTRUM OF RISHIRILIDE B (2) (DMSO-D$_6$, 500 MHZ). 11
FIGURE S9: IR AND VCD EXPERIMENTAL VS CALCULATED SPECTRA. 12
SUPPLEMENTARY NOTE 1: ANALYSIS OF IR AND VCD SPECTRA 12
TABLE S2: CARTESIAN COORDINATES OF 7AI COMPLEXES USED FOR VCD ANALYSIS. 13
FIGURE S11: AMINO ACID SEQUENCES OF RSLO9 USED IN THIS STUDY. 17
TABLE S3: DATA COLLECTION AND REFINEMENT STATISTICS FROM THE CRYSTAL STRUCTURES OF RSLO9. 18
FIGURE S12: FAD COFACTOR DENSITY MAP IN RSLO9 CRYSTAL STRUCTURE. 19
SUPPLEMENTARY NOTE 2: DESCRIPTION OF THE FAD BINDING DOMAIN OF RSLO9 19
FIGURE S13: RSLO9WT SEC MALS ANALYSIS. 20
FIGURE S14: RSLO9PAP SEC MALS ANALYSIS. 21
FIGURE S16: MULTIPLE SEQUENCE ALIGNMENT OF RSLO9 AND CHARACTERIZED HOMOLOGS 27
SUPPLEMENTARY NOTE 3: PHYLOGENETIC RELATION OF RSLO9 TO OTHER INVESTIGATED FPMOS 29
FIGURE S18: REPRESENTATIVE MELTING CURVES OF RSLO9. 30
TABLE S1: COMOUND OVERVIEW. 31
FIGURE S19: UV TRACE OF RSLO9 REACTION WITH 7. 33
FIGURE S20: UV TRACE OF RSLO9 REACTION WITH 8. 34
FIGURE S21: UV TRACE OF RSLO9 REACTION WITH 9. 35
FIGURE S22: UV TRACE OF RSLO9 REACTION WITH 10 36
FIGURE S23: UV TRACE OF RSLO9 REACTION WITH 11. 37
FIGURE S24: UV TRACE OF RSLO9 REACTION WITH 12. 38
FIGURE S25: UV TRACE OF RSLO9 REACTION WITH 13. 39
FIGURE S26: UV TRACE OF RSLO9 REACTION WITH 14. 40
FIGURE S27: UV TRACE OF RSLO9 REACTION WITH 20. 41
FIGURE S28: UV TRACE OF RSLO9 REACTION WITH 21. 42
FIGURE S29: UV TRACE OF RSLO9 REACTION WITH 22. 43
FIGURE S30: NUMBERING OF 8 AND 16. 44
FIGURE S31: COSY AND HMBC REPRESENTATION OF 16. 45
TABLE S5: NMR DATA FOR COMPOUNDS 8 AND 16 MEASURED IN DMSO-D$_6$ 46
FIGURE S32: REPRESENTATION OF LONG RANGE HMBC CORRELATIONS OF 8 AND 16. 47
FIGURE S33: ^{1}H NMR SPECTRUM OF HOOKER INTERMEDIATE (16) (DMSO-D$_6$, 500 MHZ). 48
FIGURE S35: COSY NMR SPECTRUM OF HOOKER INTERMEDIATE (16) (DMSO-D$_6$, 500 MHZ). 50
FIGURE S36: HSQC NMR SPECTRUM OF HOOKER INTERMEDIATE (16) (DMSO-D$_6$, 500 MHZ). 51
FIGURE S37: HMBC NMR SPECTRUM OF HOOKER INTERMEDIATE (16) (DMSO-D$_6$, 500 MHZ). 52
FIGURE S38: SCHEMATIC OVERVIEW OF RSLO9 REACTION WITH 8. 53
FIGURE S39: EIC SPECTRA FOR RSLO9 REACTIONS FOR THE FORMATION OF ^{18}O-LABELED 19. 54
FIGURE S40: HRMS MS1 OF 16 FROM $H_2{}^{18}O$ REACTION. 55
FIGURE S41: HRMS MS1 OF 16 FROM $^{18}O_2$ REACTION. 56
FIGURE S42: HRMS MS1 OF 19 FROM $^{18}O_2$ REACTION. 57
FIGURE S43: HMRS MS1 OF 19 FROM $H_2{}^{18}O$ REACTION. 58
FIGURE S44: HRMS MS2 OF 16. 59
FIGURE S45: HRMS MS2 OF 19 FROM THE $^{18}O_2$ REACTION. 60
FIGURE S 46: HRMS MS2 OF 19 FROM THE $H_2{}^{18}O$ REACTION. 61
FIGURE S47: REACTION OVERVIEW OF 8 METHYLATION. 62
FIGURE S48: ^{1}H NMR SPECTRUM OF METHYL-LAPACHOL (20) (DMSO-D$_6$, 500 MHZ). 63

Figure S49: ^{13}C NMR spectrum of methyl-lapachol (20) (DMSO-d_6, 125 MHz). 64
Figure S50: COSY NMR spectrum of methyl-lapachol (20) (DMSO-d_6, 500 MHz). 65
Figure S51: HSQC NMR spectrum of methyl-lapachol (20) (DMSO-d_6, 500 MHz). 66
Figure S52: HMBC NMR spectrum of methyl-lapachol (20) (DMSO-d_6, 500 MHz). 67
Figure S 53:Distance and angle representation of docked 5 relative to the FAD cofactor of RslO9. 68
Figure S54: representation of active site MSA residues of RslO9 and homologs. 69
Figure S55: RslO9WT vs H251N and H251A activity comparison. 70
Table S6: RslO9 QuickChange primer. 71
Figure S56: Size exclusion chromatograms of RslO9 H251 variatns vs RslO9 WT. 72

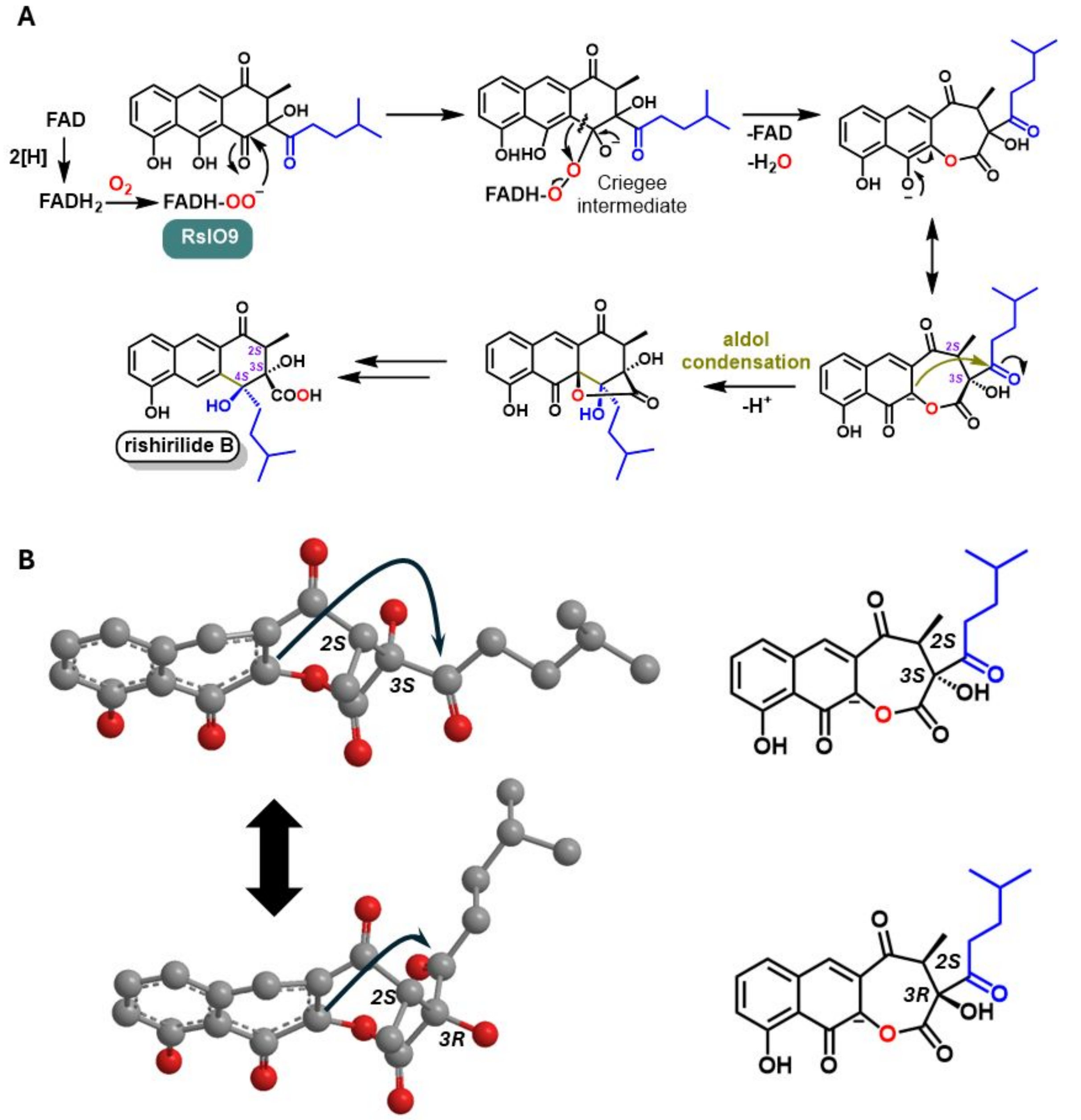

Figure S1: Previously reported RslO9 mechanism A) Previously proposed Baeyer-Villiger monooxygenase functionality of RslO9 for conversion of **5** into rishirilide B (**2**). **B**) 3D (left) and corresponding Chemdraw models (right) of carbanion lactone intermediates with two different configurations arising from a theoretical RslO9-catalyzed BV oxidation. In contrast to the 2*S*,3*S* configuration (top), the 2*S*,3*R* configuration (bottom) appears in principle compatible with a subsequent aldol condensation (arrows). However, the recently proposed 2*S*,3*S*,4*S* configuration for **2** was experimentally confirmed in this work, making a BV oxidation-type RslO9 mechanism unlikely. Note that the carbon numbering shown here is according to compound **2**.

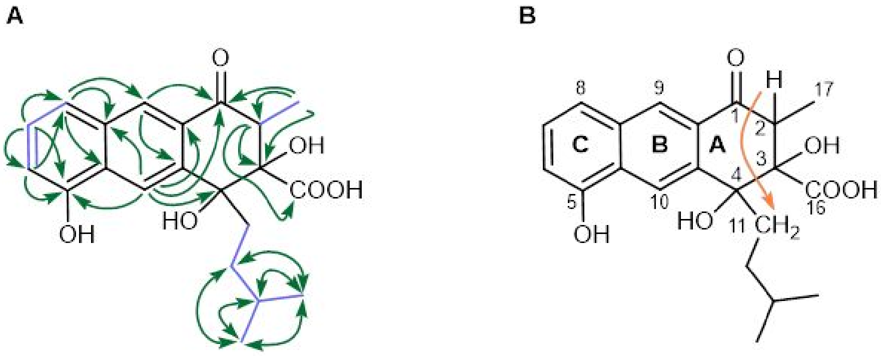

Figure S2: HMBC, COSY and key NOESY representation of 2 A) Representation of the HMBC (green arrows) and COSY (blue bonds) correlations of rishirilide B (**2**). **B)** Highlighted NOE correlation (orange) of substituents at position 2 and 4 of ring A of **2**.

Table S1: NMR spectroscopic data for rishirilide B (2) isolated in this study compared to literature values.

	This study		Tsypik et al. 2020	
position	δ_C (a)	δ_H (b)	δ_C (c)	δ_H (c)
1	197.3		197.1	
2	48.0	2.97	47.9	2.99
3	83.5		83.6	
4	76.9		76.9	
4a	140.3		140.0	
5	153.1		153.0	
6	109.8	6.91	109.9	6.93
7	126.4	7.29	126.3	7.28
8	119.8	7.46	119.7	7.46
8a	132.4		132.3	
9	125.7	8.27	125.7	8.29
9a	130.1		129.9	
10	119.6	8.26	119.6	8.28
10a	126.1		126.1	
11	35.0	2.20	35.0	2.23
		1.59		1.61
12	31.2	1.38	31.1	1.38
		0.77		0.78
13	28.0	1.30	27.8	1.30
14	22.4	0.66	22.4	0.66
15	22.6	0.76	22.6	0.77
16	174.2		174.0	
17	10.2	1.17	10.1	1.19
OH		10.27		10.20

(a) Recorded at 125 MHz, (b) Recorded at 500 MHz, the respective solvent signal of DMSO-d_6 was used to reference the NMR spectra, (c) reference shifts from Tsypik *et al.*[1]

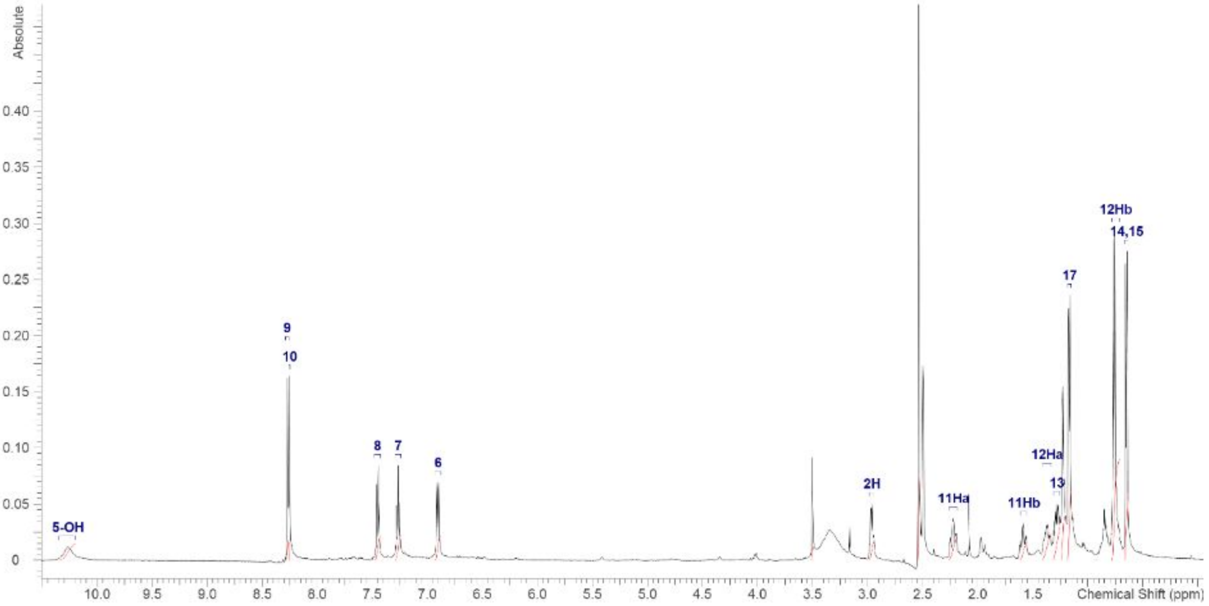

Figure S3: ^{1}H NMR spectrum of rishirilide B (2) (DMSO-d_6, 500 MHz).

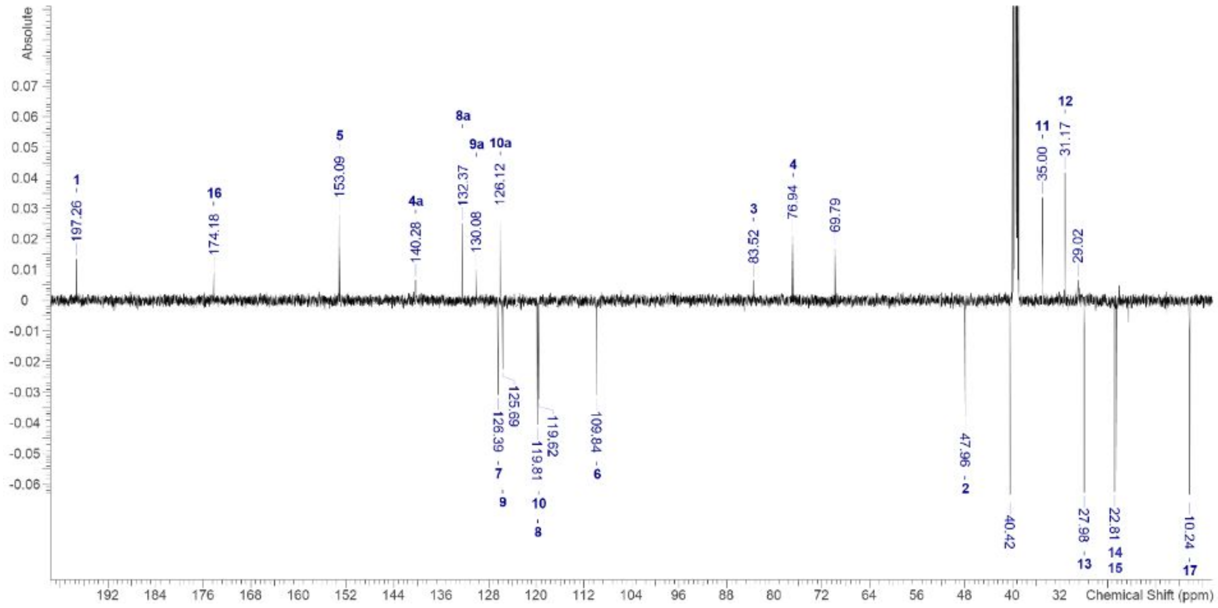

Figure S4: ^{13}C NMR spectrum of rishirilide B (2) (DMSO-d_6, 125 MHz).

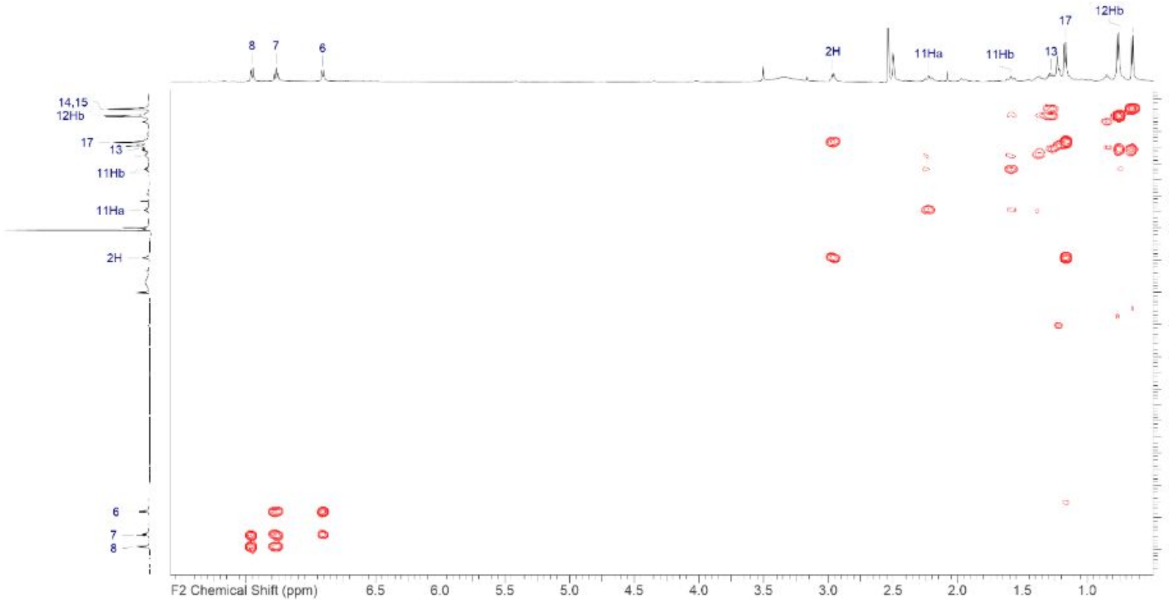

Figure S5: COSY NMR spectrum of rishirilide B (2) (DMSO-d_6, 500 MHz).

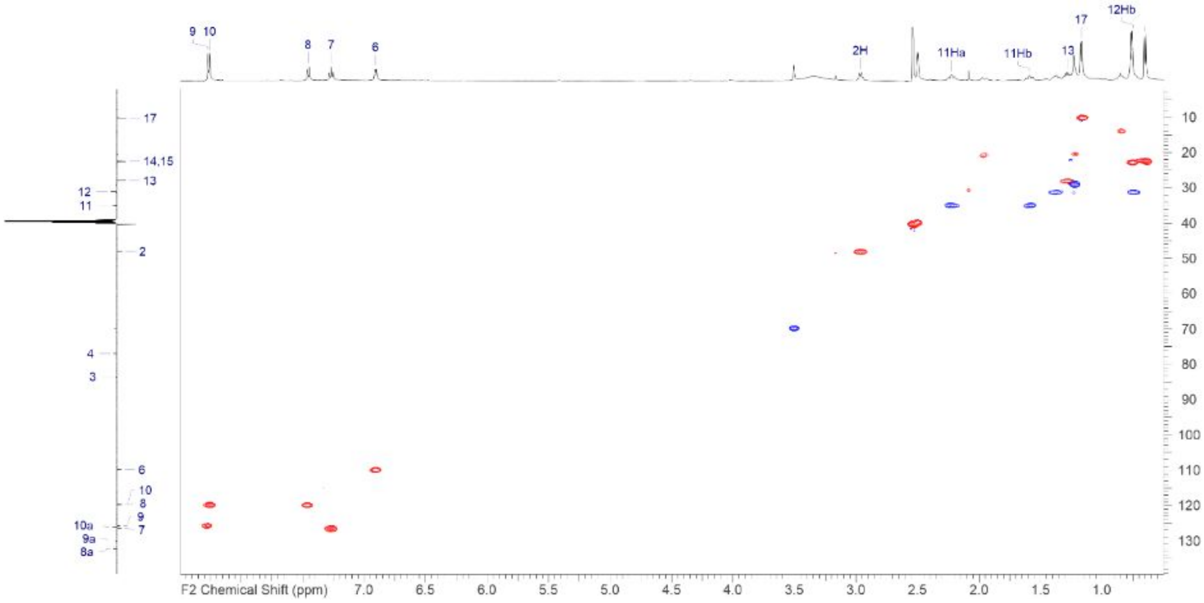

Figure S6: HSQC NMR spectrum rishirilide B (2) (DMSO-d$_6$, 500 MHz).

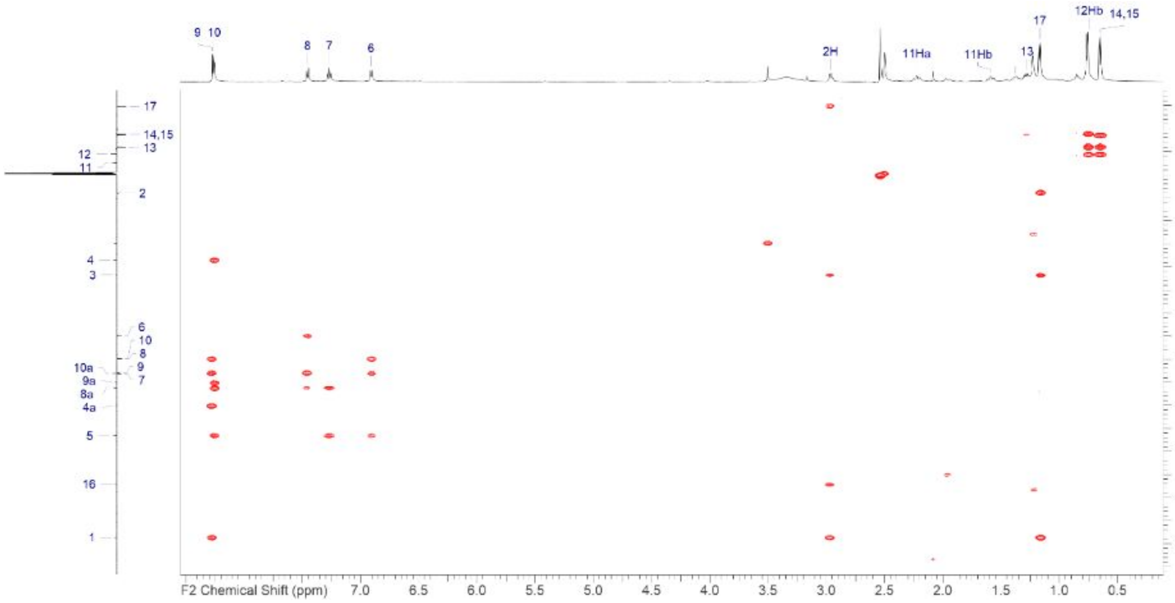

Figure S7: HMBC NMR spectrum of rishirilide B (2) (DMSO-d6, 500 MHz).

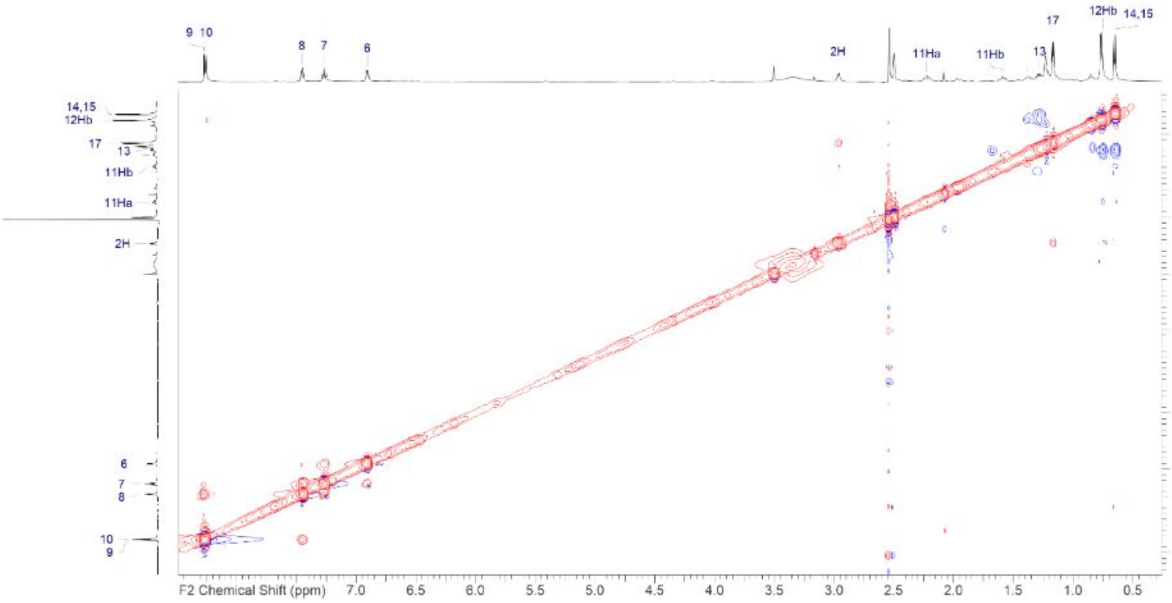

Figure S8: NOESY NMR spectrum of rishirilide B (2) (DMSO-d$_6$, 500 MHz).

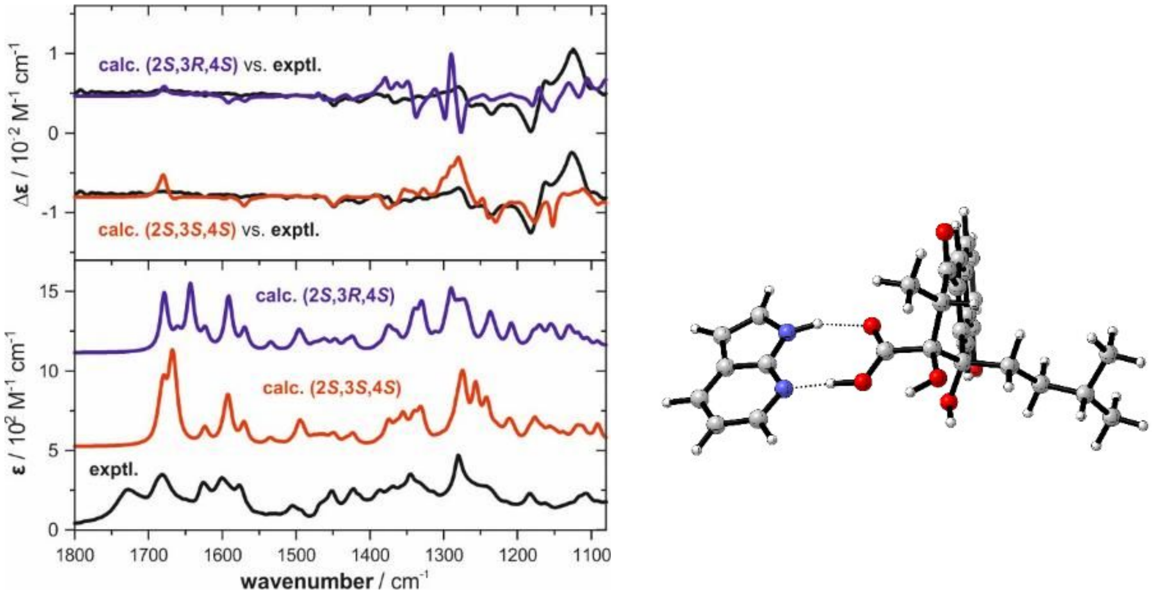

Figure S9: IR and VCD experimental vs calculated spectra. Comparison of the experimental (0.12 M, $CDCl_3$, 100 µm) and computed IR and VCD spectra of **2** (left) and lowest energy conformation of (2*S*,3*S*,4*S*)-**2**·7AI (population of 59.8%) and the (2*S*,3*R*,4*S*)-**2**·7AI diastereomer (right). VCD spectra compared to the calculated spectrum are shown on the top left. The IR experimental spectrum is compared to the calculated spectra on the bottom left.

Supplementary Note 1: Analysis of IR and VCD spectra

As the relative configurations (2*R*,4*R*) or (2*S*,4*S*) could be determined by NOESY, the configuration at carbon 3 and the absolute configuration had to be determined by means of VCD spectroscopy. Hence, calculations were carried out for the (2*S*,3*S*,4*S*)- and the (2*S*,3*R*,4*S*)-diastereomer (both as 7AI complexes). The ΔE_{ZPC}-weighted spectrum of the (2*S*,3*S*,4*S*) is basically comprised of three populated conformers, whereas (2*S*,3*R*,4*S*) is based on two conformers. Comparison of the experimental and calculated IR spectra gave a good match for both (2*S*,3*S*,4*S*) and (2*S*,3*R*,4*S*) although the simulated spectra differ above 1575 cm^{-1}. It should be noted that the shift of the C=O stretching vibration in the calculated spectra compared to the experiments might arise from the formation of the 7AI - carboxylic acid complex. In the VCD spectra, (2*S*,3*R*,4*S*) and its enantiomer clearly showed no match with the experiment. Especially the -/+/- signature between 1299 cm^{-1} and 1276 cm^{-1} does not have an experimental counterpart. In contrast, the calculated spectrum of (2*S*,3*S*,4*S*) matched the experiment of a large spectral range. The +/- signature at 1270 cm^{-1} is nicely reproduced although the intensities are slightly overpredicted in the calculations.

Table S2: Cartesian coordinates of 7AI complexes used for VCD analysis.

(2S,3S,4S)-C1··7AI

ΔE_{ZPC} = 0.0 kcal/mol, population = 59.8 %

C	-2.80373800	5.00383800	-0.52894800
C	-2.78943800	4.71275600	0.85217200
C	-2.45846000	3.45374800	1.29572200
C	-2.12689900	2.42244400	0.37225900
C	-2.14513600	2.73327000	-1.02036400
C	-2.48882800	4.04086300	-1.45141400
C	-1.79314000	1.11296800	0.79153000
C	-1.47912600	0.13120200	-0.11497300
C	-1.45966900	0.44576800	-1.50310400
C	-1.79529300	1.71319100	-1.92819900
C	-1.22711000	-1.29447900	0.33698300
C	-0.13189700	-1.97597000	-0.55901500
C	-0.51420100	-1.91597600	-2.05572100
C	-0.98627700	-0.53596600	-2.51928400
O	-0.98703700	-0.26649600	-3.70864200
H	-3.06708600	6.00406600	-0.85087300
H	-3.04006600	5.48691700	1.56969600
H	-2.49657500	4.26269900	-2.51166900
H	-1.78862600	0.88675600	1.84686300
H	-1.76085900	1.92994800	-2.98873600
O	-0.73250300	-1.25811000	1.67713800
H	-0.60076900	-2.17448500	1.95565300
C	-2.54760300	-2.11640400	0.28123500
H	-2.92861600	-2.10013600	-0.74114900
H	-2.29448500	-3.15577500	0.50518700
C	-3.64325200	-1.64388800	1.24204200
H	-3.99735200	-0.64963200	0.95128700
H	-3.21802300	-1.53913800	2.24356100
C	-4.85220200	-2.59456200	1.32175800
H	-4.47538700	-3.59235700	1.58162600
C	-5.60594400	-2.70799800	-0.00921400
H	-5.97607900	-1.72840700	-0.32908300
H	-6.46829900	-3.37264200	0.08832800
H	-4.97648800	-3.10437500	-0.80860400
C	-5.80280700	-2.14935800	2.43945300
H	-6.20838400	-1.15393300	2.23215800
H	-5.29023100	-2.10540000	3.40397500
H	-6.64659800	-2.83734600	2.53792400
C	1.22463500	-1.27109400	-0.33191100
O	2.10266100	-2.02052800	0.29741600
H	3.04071400	-1.55459900	0.43541200
O	1.43664900	-0.14172700	-0.73779500
O	-0.03928000	-3.34034800	-0.15658300
H	0.86806300	-3.49480400	0.14311800
O	-2.43061500	3.12151600	2.62098800
H	-2.67458400	3.88901900	3.15321900
C	0.55964300	-2.49470600	-2.98193800
H	0.18706300	-2.54263600	-4.00347900
H	0.82557700	-3.50221500	-2.66204400
H	1.45936000	-1.87612700	-2.98512100
H	-1.40027700	-2.55477700	-2.14636000
C	4.78724700	0.26241800	0.35015700
C	6.05586600	0.86961900	0.56546600
C	7.02923900	0.09233000	1.19168700
C	6.69312900	-1.20641500	1.55650000
C	5.41314600	-1.70137600	1.29626400
N	4.45551600	-0.98275800	0.69749600
H	8.02000200	0.48367900	1.39070100
H	7.41451100	-1.84791900	2.04479300
H	5.14403200	-2.71236400	1.57848300
C	5.95874000	2.19776800	0.03885000
H	6.72736000	2.95330000	0.02592200
C	4.68855400	2.33397400	-0.45316400
H	4.22315700	3.18152200	-0.92962800
N	3.97726400	1.16741700	-0.26701800
H	3.01542500	0.96628700	-0.53365300

(2S,3S,4S)-C2··7AI

ΔE_{ZPC} = 0.7 kcal/mol, population = 17.5 %

C	-2.81940100	5.00098100	-0.52938600
C	-2.78017100	4.72479400	0.85109900
C	-2.43399900	3.47329300	1.30548000
C	-2.11757200	2.42979700	0.38790900
C	-2.15417100	2.73102600	-1.00950200
C	-2.50987400	4.03077800	-1.44779300
C	-1.78190800	1.11537400	0.79587100
C	-1.47818800	0.13098700	-0.10971500
C	-1.46786000	0.44316100	-1.49776300
C	-1.81045400	1.70913000	-1.91844000
C	-1.22973600	-1.29446700	0.34250700
C	-0.13321900	-1.97478300	-0.55173100
C	-0.51537500	-1.91601900	-2.04910300
C	-0.99600800	-0.53904800	-2.51390000
O	-1.00343200	-0.27133200	-3.70331700
H	-3.09369000	5.99608600	-0.85800100
H	-3.01800600	5.49485300	1.57442000
H	-2.53177100	4.24147000	-2.51004300
H	-1.76189500	0.84782000	1.84436300
H	-1.78708500	1.92606100	-2.97930500
O	-0.73969200	-1.25774600	1.68472200
H	-0.60716900	-2.17389200	1.96383000
C	-2.55134600	-2.11431700	0.28267800
H	-2.92929000	-2.09693500	-0.74067600
H	-2.29991100	-3.15400400	0.50667500
C	-3.64945000	-1.64090800	1.24024300
H	-3.99951800	-0.64477100	0.95056500
H	-3.22833500	-1.54185100	2.24425900
C	-4.86220200	-2.58749900	1.31212700
H	-4.48979700	-3.58740900	1.56989200
C	-5.61118400	-2.69326400	-0.02210100
H	-5.97685400	-1.71132400	-0.33975800
H	-6.47609400	-3.35522400	0.07005600
H	-4.98028700	-3.08915100	-0.82056600
C	-5.81518000	-2.14252500	2.42785200
H	-6.21639300	-1.14489600	2.22269900
H	-5.30638900	-2.10457300	3.39468100
H	-6.66193500	-2.82752700	2.52050400
C	1.22248900	-1.26782500	-0.32419900
O	2.10419100	-2.01873800	0.29648500
H	3.04533300	-1.55192800	0.43305500
O	1.42853600	-0.13454300	-0.72280900
O	-0.04042300	-3.33798800	-0.14719800
H	0.86921500	-3.49377200	0.14512100
O	-2.41804400	3.28196000	2.65872200
H	-2.09940800	2.39817000	2.87524400
C	0.56268200	-2.48789800	-2.97475700
H	0.19088600	-2.53737500	-3.99648600
H	0.83410200	-3.49413600	-2.65545900
H	1.45882300	-1.86418700	-2.97694700
H	-1.39711800	-2.56061400	-2.14047400
C	4.78720300	0.26047200	0.34193100
C	6.05848000	0.86368500	0.55240400
C	7.03222600	0.08316600	1.17391400
C	6.69412000	-1.21499100	1.53915100
C	5.41186000	-1.70623200	1.28398500
N	4.45373700	-0.98415400	0.68988400
H	8.02491800	0.47156800	1.36893900
H	7.41585300	-1.85888400	2.02371700
H	5.14098200	-2.71674300	1.56611000
C	5.96319800	2.19246900	0.02703600
H	6.73409600	2.94560200	0.01140800
C	4.69156500	2.33289900	-0.45970400
H	4.22684200	3.18210900	-0.93384900

N	3.97736500	1.16825700	-0.27121000
H	3.01394600	0.97040900	-0.53408600

(2S,3S,4S)-C3··7AI

ΔE_{ZPC} = 0.8 kcal/mol, population = 15.4 %

C	-1.55503800	5.21209000	-0.72916500
C	-1.60785200	4.98229200	0.66204100
C	-1.57769100	3.70039100	1.15900200
C	-1.49437100	2.58413600	0.28007000
C	-1.44103300	2.83313000	-1.12432500
C	-1.47219300	4.16567000	-1.60999400
C	-1.47089200	1.25037700	0.75243600
C	-1.39135500	0.18726100	-0.11248000
C	-1.30916000	0.43143200	-1.51542100
C	-1.34326700	1.72425600	-1.99029100
C	-1.43918300	-1.24856600	0.39321700
C	-0.46880500	-2.12092700	-0.49650100
C	-0.92616700	-2.10203300	-1.96464000
C	-1.11046700	-0.67928900	-2.49654400
O	-1.12419600	-0.47071800	-3.69675100
H	-1.57967500	6.23212300	-1.09284900
H	-1.67135500	5.82148100	1.34599400
H	-1.42926700	4.33992900	-2.67817100
H	-1.52478300	1.06961500	1.81618100
H	-1.27075700	1.88619000	-3.05873700
O	-1.04683800	-1.36764500	1.76099500
H	-0.10709400	-1.15156900	1.84732200
C	-2.88621900	-1.80297300	0.33906200
H	-3.27881900	-1.65495500	-0.66658700
H	-2.83300700	-2.88105400	0.50926900
C	-3.85014800	-1.17632900	1.35208600
H	-3.95629900	-0.10366100	1.15719400
H	-3.42441600	-1.27063200	2.35290500
C	-5.25022700	-1.81833600	1.35152400
H	-5.12121800	-2.89915000	1.49342200
C	-6.00375900	-1.60246700	0.03310100
H	-6.13375800	-0.53401900	-0.16802900
H	-6.99783600	-2.05510900	0.07572700
H	-5.48214100	-2.04159800	-0.81979800
C	-6.07438300	-1.28876400	2.53116800
H	-6.22905600	-0.20849600	2.44411400
H	-5.57222900	-1.47750500	3.48355700
H	-7.05873100	-1.76264700	2.56998600
C	0.97127100	-1.55332900	-0.37107000
O	1.54105100	-1.85022100	0.78223000
H	2.51348500	-1.47010000	0.87093500
O	1.49695100	-0.88487700	-1.24373000
O	-0.47873100	-3.46888800	-0.05610300
H	-0.32821200	-3.46388700	0.89986900
O	-1.62556700	3.42579000	2.49671800
H	-1.69140900	4.24984800	2.99581300
C	-0.09107300	-2.97641300	-2.90600100
H	-0.54210900	-2.97975200	-3.89717400
H	-0.06027300	-4.00015700	-2.53442100
H	0.92801900	-2.60287900	-2.99877500
H	-1.93632600	-2.52476500	-1.95758900
C	4.63655300	-0.19034500	0.27209500
C	5.95265200	0.32125900	0.44618900
C	6.60710400	-0.01193700	1.63147800
C	5.93275800	-0.81174700	2.54678800
C	4.63800200	-1.25852000	2.27101400
N	3.98028200	-0.95738400	1.14473300
H	7.61145700	0.33885900	1.83786500
H	6.39892200	-1.09785000	3.48021500
H	4.10727200	-1.88251800	2.98044200
C	6.24572300	1.08379400	-0.73011100
H	7.15428400	1.61969000	-0.95182000
C	5.14144600	1.00550000	-1.53522000
H	4.96576400	1.44132400	-2.50534900
N	4.16667600	0.23762800	-0.93368500
H	3.24614200	-0.00365800	-1.29275900

(2S,3R,4S)-C1··7AI

ΔE_{ZPC} = 0.0 kcal/mol, population = 82.1 %

C	7.22911900	-0.91996300	0.41304600
C	6.83961200	-0.20777500	1.56814500
C	5.51842700	0.11046500	1.77837000
C	4.52169100	-0.27055800	0.83603000
C	4.93192300	-0.99071500	-0.32581800
C	6.30186700	-1.30748600	-0.51792700
C	3.15435100	0.04391600	1.01644600
C	2.20448400	-0.33215000	0.09929600
C	2.60692700	-1.06877000	-1.05041200
C	3.93783600	-1.37527400	-1.24708600
C	0.73970900	0.07036600	0.28058600
C	-0.12874500	-1.14638600	-0.21316400
C	0.12300800	-1.46963600	-1.70253300
C	1.61442500	-1.58234700	-2.03125700
O	1.96425100	-2.08086100	-3.08801600
C	-1.63627100	-0.92627600	0.03414300
O	-2.20974600	-0.04760000	-0.73907900
O	-2.21198900	-1.59368400	0.88716500
O	0.26057800	-2.25084600	0.60107300
H	8.27655500	-1.15808700	0.27333300
H	7.58755000	0.08866400	2.29593300
H	6.59831700	-1.85615500	-1.40353800
H	2.85745000	0.59551500	1.89607300
H	4.21440400	-1.94468100	-2.12587000
H	-3.23603700	0.06197200	-0.55261200
H	-0.54922600	-2.55256600	1.04809800
O	0.43371700	0.28890900	1.65525700
H	0.56456500	-0.55631400	2.10904700
C	0.44282000	1.40051500	-0.44739600
H	0.61151100	1.25776500	-1.51582400
H	-0.61768300	1.61959900	-0.32210500
C	1.25748600	2.60181500	0.04677200
H	2.31790500	2.46530300	-0.18811200
H	1.18335500	2.65525800	1.13568300
C	0.79229300	3.94714600	-0.54021200
H	-0.27816000	4.05344100	-0.32175300
C	0.97292700	4.02437100	-2.06140600
H	2.02517700	3.89143600	-2.33366200
H	0.65270300	4.99793500	-2.44174500
H	0.39357100	3.26184700	-2.58613800
C	1.52525500	5.10603500	0.14583500
H	1.37007400	5.08963500	1.22773600
H	1.17672400	6.07180900	-0.22953100
H	2.60302900	5.04802300	-0.03720600
O	5.08821400	0.79696100	2.87868900
H	5.84058500	1.01140200	3.44414000
C	-0.63925200	-2.71022200	-2.18419300
H	-0.35743000	-3.58913100	-1.60338800
H	-0.41063500	-2.90153600	-3.23078400
H	-1.71682100	-2.56397100	-2.09218800
H	-0.23094300	-0.61196600	-2.28342300
C	-5.51164900	-0.29642700	0.53522900
C	-6.90366600	-0.10286500	0.75463300
C	-7.54877400	0.82197300	-0.06564200
C	-6.79204400	1.48027100	-1.02797000
C	-5.42702200	1.21097800	-1.15519300
N	-4.77655200	0.33017200	-0.38522200
H	-8.60795300	1.02533700	0.03968600
H	-7.24735500	2.20651600	-1.68800500
H	-4.83138700	1.72098200	-1.90308800
C	-7.26585800	-0.97540300	1.83110600
H	-8.24165500	-1.09879800	2.27200700
C	-6.12589100	-1.63454000	2.20421900
H	-5.98032500	-2.37351300	2.97541200
N	-5.06347700	-1.22769800	1.42423400
H	-4.09779300	-1.54242100	1.46615900

(2S,3R,4S)-C2··7AI

ΔE_{ZPC} = 1.1 kcal/mol, population = 12.7 %

C	7.16519300	-1.14818400	0.56399900
C	6.75664900	-0.46736600	1.73134000
C	5.44161800	-0.10302300	1.90163800
C	4.47073600	-0.40296100	0.90457200
C	4.89994500	-1.09249400	-0.26898100
C	6.26277300	-1.45896900	-0.41871100
C	3.11104000	-0.03740400	1.04188500
C	2.18698800	-0.33427600	0.07092500
C	2.60701800	-1.04037600	-1.09142600
C	3.93109600	-1.39660700	-1.24566400
C	0.73527700	0.12759800	0.20544300
C	-0.16882900	-1.02236900	-0.37602700
C	0.13861900	-1.30093900	-1.86360000
C	1.63595100	-1.46728000	-2.13372600
O	2.00953400	-1.93875000	-3.19498300
C	-1.66500000	-0.67156600	-0.23270900
O	-2.28310700	-1.39605200	0.67268700
O	-2.19945200	0.17618900	-0.92843700
O	0.13934600	-2.17688000	0.41006900
H	8.20716400	-1.42399600	0.45643900
H	7.48482100	-0.23160000	2.50021000
H	6.57368600	-1.98341300	-1.31398600
H	2.79932600	0.49068500	1.93080200
H	4.22191400	-1.94123900	-2.13546600
H	-3.31411800	-1.18443000	0.76112900
H	-0.68940600	-2.50978100	0.78262400
O	0.37741000	0.30698900	1.57355500
H	0.44105600	-0.56204300	1.99556300
C	0.52986100	1.49492200	-0.48359500
H	0.76265700	1.38681100	-1.54402100
H	-0.52924200	1.74579200	-0.41826100
C	1.35162200	2.64506800	0.10976200
H	2.42035600	2.47411400	-0.05482800
H	1.20170000	2.66432000	1.19187400
C	0.98053300	4.02691900	-0.45914900
H	-0.09875100	4.16695600	-0.31628200
C	1.27728100	4.14970900	-1.95898300
H	2.34177300	3.98705200	-2.15757900
H	1.02044600	5.14711100	-2.32541400
H	0.71277300	3.42793000	-2.55292200
C	1.70128900	5.13223900	0.32172400
H	1.46423300	5.08511500	1.38777900
H	1.41767000	6.12316400	-0.04265000
H	2.78699400	5.03832900	0.21723600
O	4.99381100	0.55526200	3.01217200
H	5.72970800	0.71277500	3.61669400
C	-0.65312700	-2.48817600	-2.42615200
H	-0.42726100	-3.40263700	-1.87630500
H	-0.39367100	-2.64382900	-3.47144600
H	-1.72654300	-2.30115100	-2.36602600
H	-0.15692500	-0.40662400	-2.42140600
C	-5.52671300	-0.06846400	0.29465000
C	-6.91784400	0.19094200	0.43902500
C	-7.60646800	-0.56891800	1.38377700
C	-6.89066800	-1.51495400	2.10850500
C	-5.52264300	-1.68607500	1.88393100
N	-4.83149300	-0.97427800	0.98523900
H	-8.66791100	-0.43032800	1.55240800
H	-7.38074000	-2.12833200	2.85275100
H	-4.95828500	-2.42111900	2.44540900
C	-7.23031000	1.23164800	-0.49397400
H	-8.19160700	1.68783700	-0.66590700
C	-6.06404800	1.54641400	-1.13769400
H	-5.88017000	2.27740600	-1.90829800
N	-5.03196600	0.76318900	-0.66465100
H	-4.05480100	0.76832800	-0.94945100

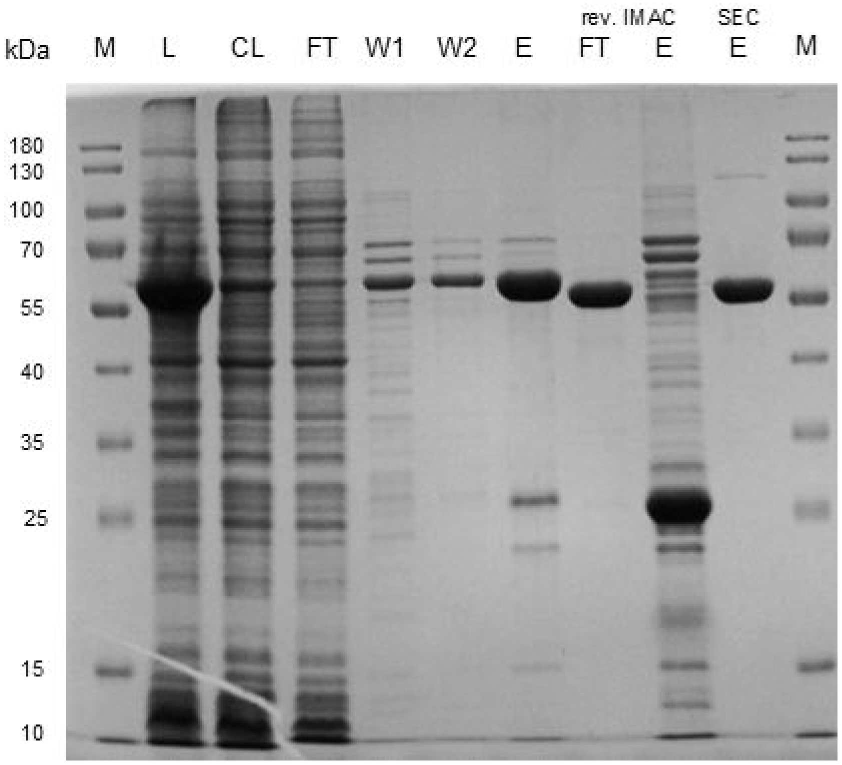

Figure S10: SDS PAGE of RslO9 purification. 12% SDS PAGE gel of the IMAC, TEV cleavage and SEC purification of RslO9PAP. The molecular mass of the marker (M) (Thermo Fisher Scientific PageRuler™ Prestained Protein Ladder #26616) is given in kDa on the left. The following fractions of the IMAC purification are lysate (L), cleared lysate (CL), flow through of the IMAC column (FT), wash fractions 1 and 2 (W1/W2) and IMAC elution (E). Purification after tag-cleavage by TEV protease gave flow through (FT) and elution (E) of the reverse IMAC procedure. The last lane before the marker shows the elution fraction of the size exclusion chromatography (SEC E).

>AHL46732.1 FAD dependent monooxygenase [*Streptomyces bottropensis*]

MPVLVAGAGPGGLSTAVFLGLHGVPALVVERHPGTSTAVKATGQYPHTMEALAIAG
AAGTVRERGRAYRSDFHMVVAKTLAGPVLRTLMSGDQLSMRHVSPEDWGTASQS
AVESVLADRAAELGSRLRFSTRLTSLTQDADGVTAVTQHTGTGERRVIRARYLVVA
DGWRSGIRQSLGIEMRGRGTVGKVLRVLFEADLSEPLSHTDGAADGRRFTALHVG
RAVLFNTEIPGLYGYFRNLTPELPDGWWTNEDTVAAQIRSDLGIPDIPLKIEEISETEI
SCGVAERFREGRALLVGDAAHVMPPTGGMGGNTAYLDGLYLGWKLAAVLRGTAG
EALLDSHDAERRPYAEELVEQQFANLVDRISPELADESLAVALPPPVVAFGYRFPKG
AVLLEPDDDGELFEDPSRPTGRPGSHAPYVPLTRRDGSATSTTALFGHAFVLLTGP
DGAAWAEGALASADALGVAVQVHRVGPGSELLDAEGAFSTAYGIGADGAALVRPD
RFVAWRCTGTHPDPAAEIERALRHVLHRPAH

> RslO9PAP with His-Tag from the pET28a plasmid

MGSSHHHHHHSSGLVPRGSHMPAPDETVPVLVAGAGPGGLSTAVFLGLHGVPALV
VERHPGTSTAVKATGQYPHTMEALAIAGAAGTVRERGRAYRSDFHMVVAKTLAGP
VLRTLMSGDQLSMRHVSPEDWGTASQSAVESVLADRAAELGSRLRFSTRLTSLTQ
DADGVTAVTQHTGTGERRVIRARYLVVADGWRSGIRQSLGIEMRGRGTVGKVLRV
LFEADLSEPLSHTDGAADGRRFTALHVGRAVLFNTEIPGLYGYFRNLTPELPDGWW
TNEDTVAAQIRSDLGIPDIPLKIEEISETEISCGVAERFREGRALLVGDAAHVMPPTG
GMGGNTAYLDGLYLGWKLAAVLRGTAGEALLDSHDAERRPYAEELVEQQFANLVD
RISPELADESLAVALPPPVVAFGYRFPKGAVLLEPDDDGELFEDPSRPTGRPGSHA
PYVPLTRRDGSATSTTALFGHAFVLLTGPDGAAWAEGALASADALGVAVQVHRVG
PGSELLDAEGAFSTAYGIGADGAALVRPDRFVAWRCTGTHPDPAAEIERALRHVLH
RPAH

>RslO9WT sequence of the alternative start codon

TQRARRPDPAAPPTPVPAPAPAPAPAPAPDETVPVLVAGAGPGGLSTAVFLGLHGV
PALVVERHPGTSTAVKATGQYPHTMEALAIAGAAGTVRERGRAYRSDFHMVVAKTL
AGPVLRTLMSGDQLSMRHVSPEDWGTASQSAVESVLADRAAELGSRLRFSTRLTS
LTQDADGVTAVTQHTGTGERRVIRARYLVVADGWRSGIRQSLGIEMRGRGTVGKV
LRVLFEADLSEPLSHTDGAADGRRFTALHVGRAVLFNTEIPGLYGYFRNLTPELPDG
WWTNEDTVAAQIRSDLGIPDIPLKIEEISETEISCGVAERFREGRALLVGDAAHVMPP
TGGMGGNTAYLDGLYLGWKLAAVLRGTAGEALLDSHDAERRPYAEELVEQQFANL
VDRISPELADESLAVALPPPVVAFGYRFPKGAVLLEPDDDGELFEDPSRPTGRPGS
HAPYVPLTRRDGSATSTTALFGHAFVLLTGPDGAAWAEGALASADALGVAVQVHR
VGPGSELLDAEGAFSTAYGIGADGAALVRPDRFVAWRCTGTHPDPAAEIERALRHV
LHRPAH

Figure S11: Amino acid sequences of RslO9 used in this study. Amino acid sequences of the RslO9 wild type and RslO9PAP construct used in this work compared to the reported sequence from NCBI. Sequences are presented in FASTA format and first 5 common amino acids of the sequences are highlighted in orange.

Table S3: Data collection and refinement statistics from the crystal structures of RslO9. Statistics for the highest-resolution shell are shown in brackets.

**Protein**	RslO9 PAP	RslO9 PAP soaked	RslO9 wt
**PDB ID**	9QM2	9QM3	9QM4
**Beam line**	SLS Beamline X06SA	DLS i03	DLS i03
**Wavelength (Å)**	0.99999	0.976246	0.97628
**Space group**	$C\ 2\ 2\ 2_1$	$C\ 2\ 2\ 2_1$	$C\ 2\ 2\ 2_1$
**Unit cell**			
**a, b, c (Å)**	111.67, 118.23, 104.46	112.15, 116.77, 104.68	112.578, 115.998, 104.8
**α, β, γ (°)**	90, 90, 90	90, 90, 90	90, 90, 90
**Resolution range (Å)**	49.24 - 2.4 (2.49 - 2.4)	49.43 - 3.1 (3.34 - 3.1)	49.59 - 2.7 (2.84 - 2.7)
**R_{merge}**	0.2353 (2.233)	0.3568 (1.685)	0.1952 (1.261)
**Mean I / σ(I)**	8.96 (1.49)	8.28 (1.82)	8.19 (1.80)
**$CC_{1/2}$**	0.996 (0.625)	0.991 (0.879)	0.997 (0.921)
**Completeness (%)**	99.80 (99.15)	99.50 (99.56)	99.66 (99.33)
**Multiplicity**	12.2 (8.7)	12.9 (14.0)	13.2 (13.4)
**R_{work}**	0.2110 (0.3281)	0.2223 (0.2634)	0.2209 (0.3426)
**R_{free}**	0.2587 (0.3797)	0.2715 (0.3074)	0.2596 (0.3804)
**Average B-factors**			
**Protein**	55.46	69.19	62.67
**Ligands**	47.52	68.53	59.21
**Solvent**	49.43	no solvent modelled	52.30
**RMSD**			
**Bonds (Å)**	0.002	0.002	0.002
**Angle (°)**	0.51	0.48	0.54
**Ramachandran plot**			
**In favored regions**	96.62	95.08	95.37
**In allowed regions**	3.38	4.92	4.63
**Outliers**	0.00	0.00	0.00

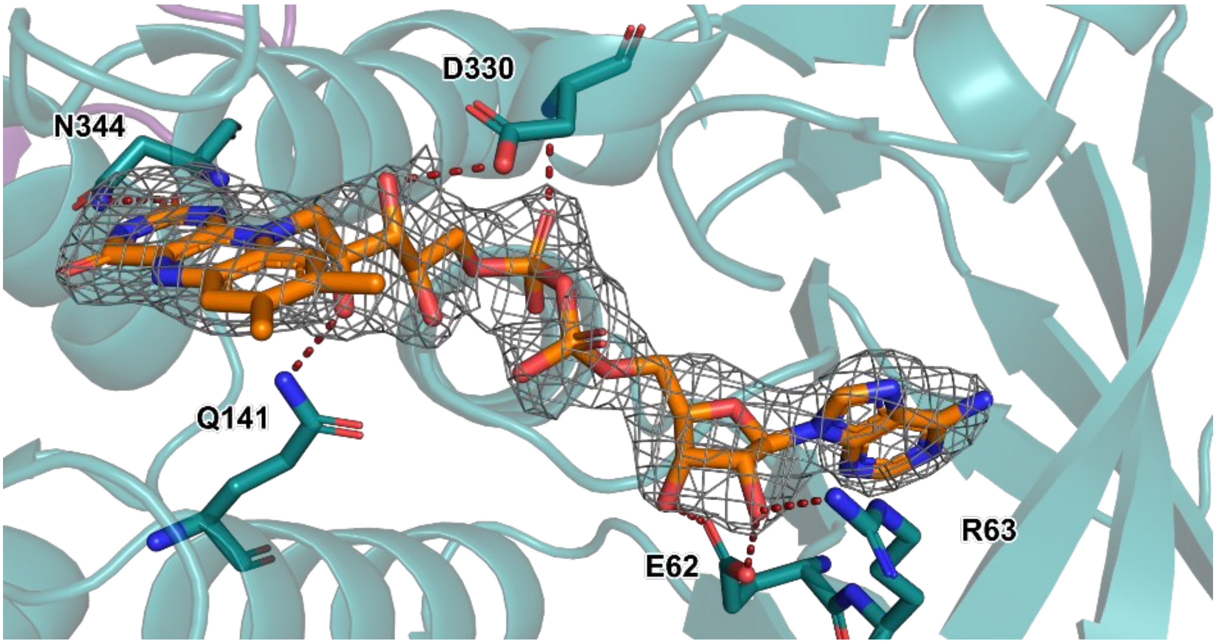

Figure S12: FAD cofactor density map in RslO9 crystal structure. Overview of the polar interactions of conserved amino acids of the FAD-binding domain (green cartoon) to the FAD cofactor in the RslO9 WT crystal structure. The σA weighted 2Fo–Fc electron density map (grey mesh) around the FAD (orange sticks) is contoured at 1.5 σ. The dashed firebrick lines show the hydrogen bond interactions of the FAD cofactor with selected amino acids. Amino acids coordinating the FAD cofactor are labeled with their single letter code.

Supplementary Note 2: Description of the FAD binding domain of RslO9

The FAD-binding domain of RslO9 adopts a typical beta-alpha-beta Rossmann fold with interactions of conserved amino acids and water molecules to the non-covalently bound FAD cofactor. Q141, D330 and N344 are involved in binding the isoalloxazine ring, the ribose moiety and the phosphate. In addition, amino acids E62 and R63 contribute to the binding as part of the dinucleotide binding motif opposed to the conserved GxGxxG Rossmann-fold sequence[2]. The middle-domain of RslO9 is comprised of a seven-stranded beta-sheet with an alpha-helix stacked on top. In conjunction with the FAD-binding domain, the middle domain forms the active site of the enzyme with the substrate binding pocket right next to the isoalloxazine ring of the FAD.

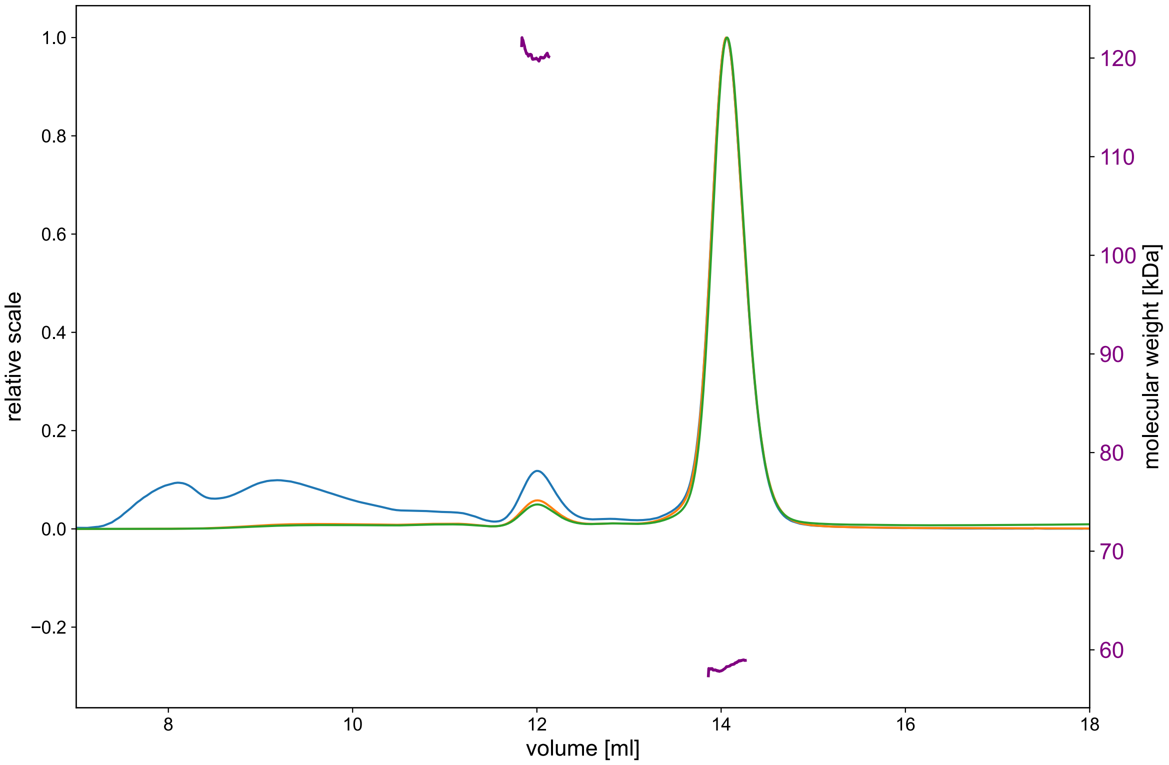

Figure S13: RslO9WT SEC MALS analysis. SEC MALS data of the RslO9WT construct with the elution volume shown on the x-axis in mL, the relative scale of the Rayleigh ratio (blue), dRI (orange) and UV absorption on the left y-axis and the molecular mass in kDa (purple line). The peak around 14 min shows a mass of 58.3 kDa with an uncertainty of 0.9% and around 95% mass fraction.

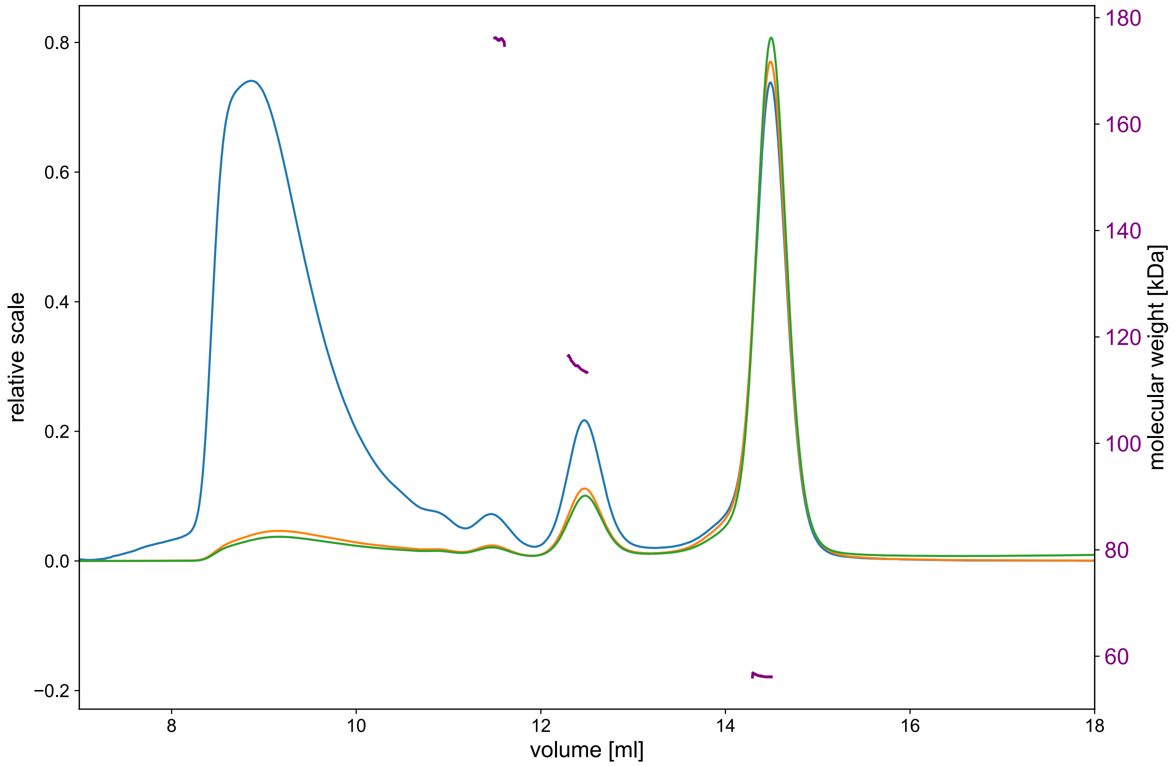

Figure S14: RslO9PAP SEC MALS analysis. SEC MALS data of the RslO9PAP construct with the elution volume shown on the x-axis in ml, the relative scale of the Rayleigh ratio (blue), dRI (orange) and UV absorption on the left y-axis and the molecular mass in kDa (purple line). The peak around 14.4 min shows a mass of 56.2 kDa with an uncertainty of 0.7% and around 90% mass fraction.

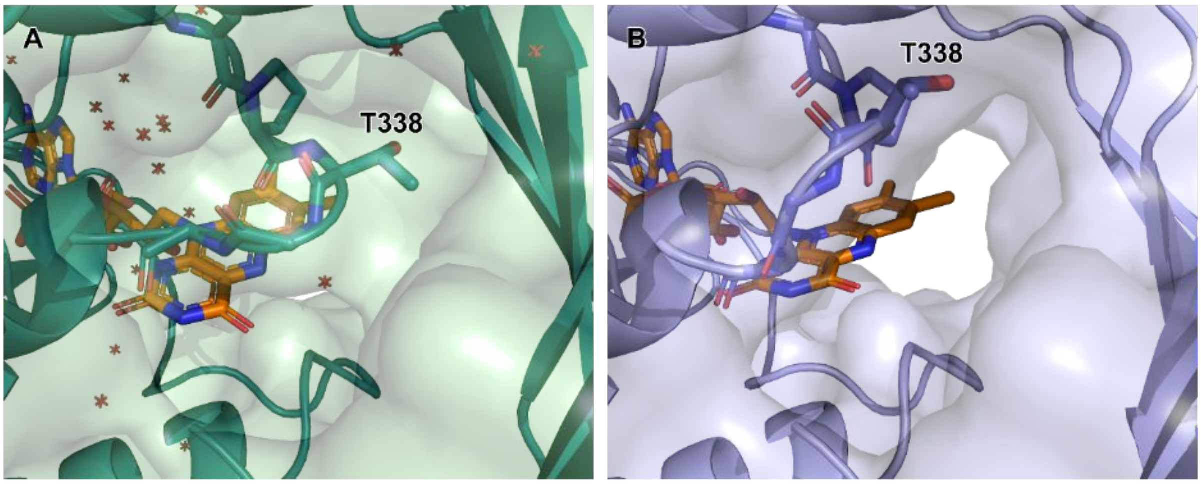

Figure S15: RslO9 active site crystal structure comparison. Active site architecture of RslO9 in the unsoaked crystal structure (**A**) highlighting the occlusion of the substrate binding site by the PPTGG-loop (threonine 338 labeled with its one letter code) and the structure when soaked with natural substrate **5** (**B**), in which the PPTGG-loop flipped upwards.

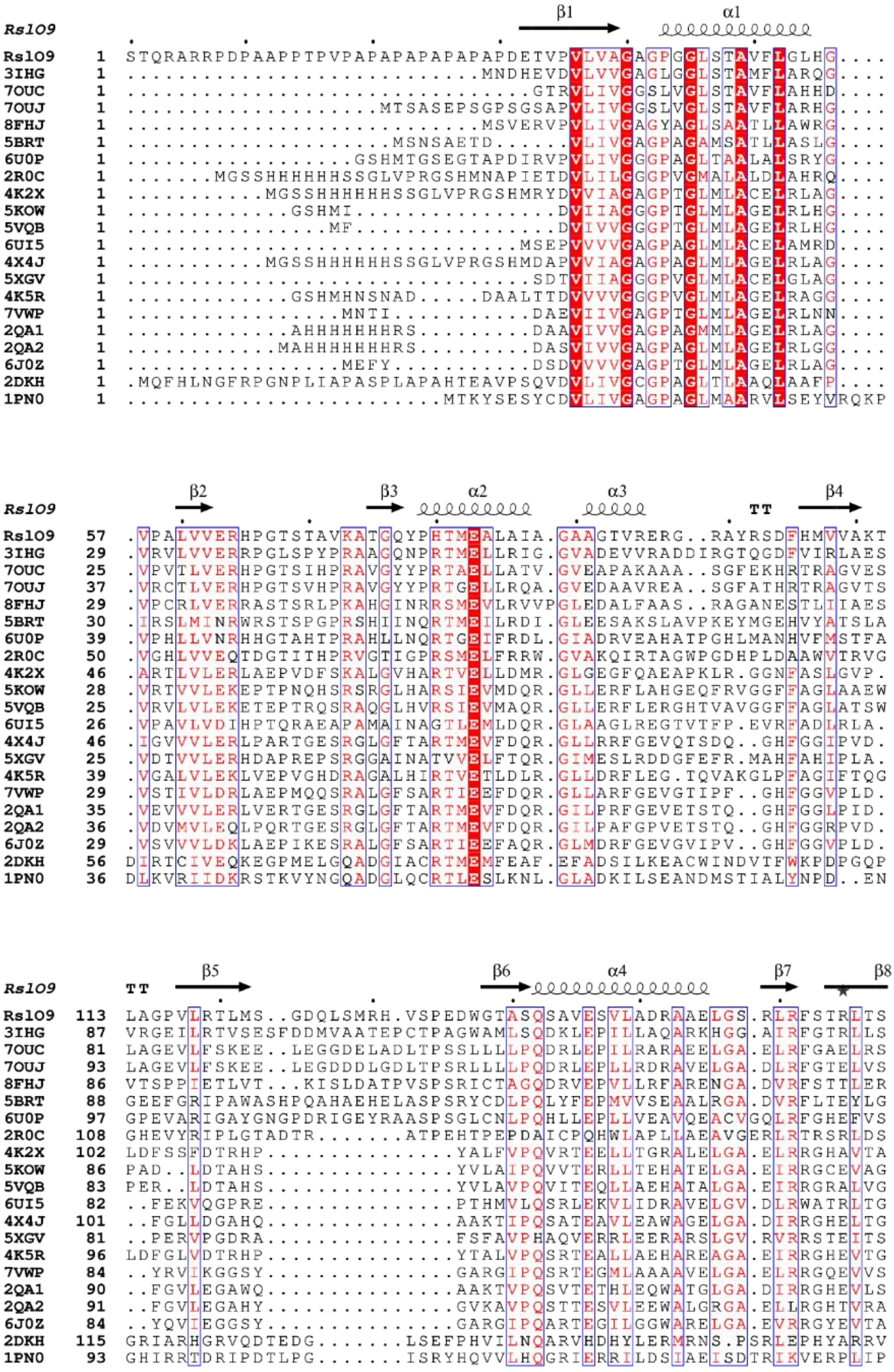
β1
α1
β2
β3
α2
α3
β4
TT
β5
β6
α4
β7
β8
Rs109
3IHG
7OUC
7OUJ
8FHJ
5BRT
6U0P
2R0C
4K2X
5KOW
5VQB
6UI5
4X4J
5XGV
4K5R
7VWP
2QA1
2QA2
6J0Z
2DKH
1PN0

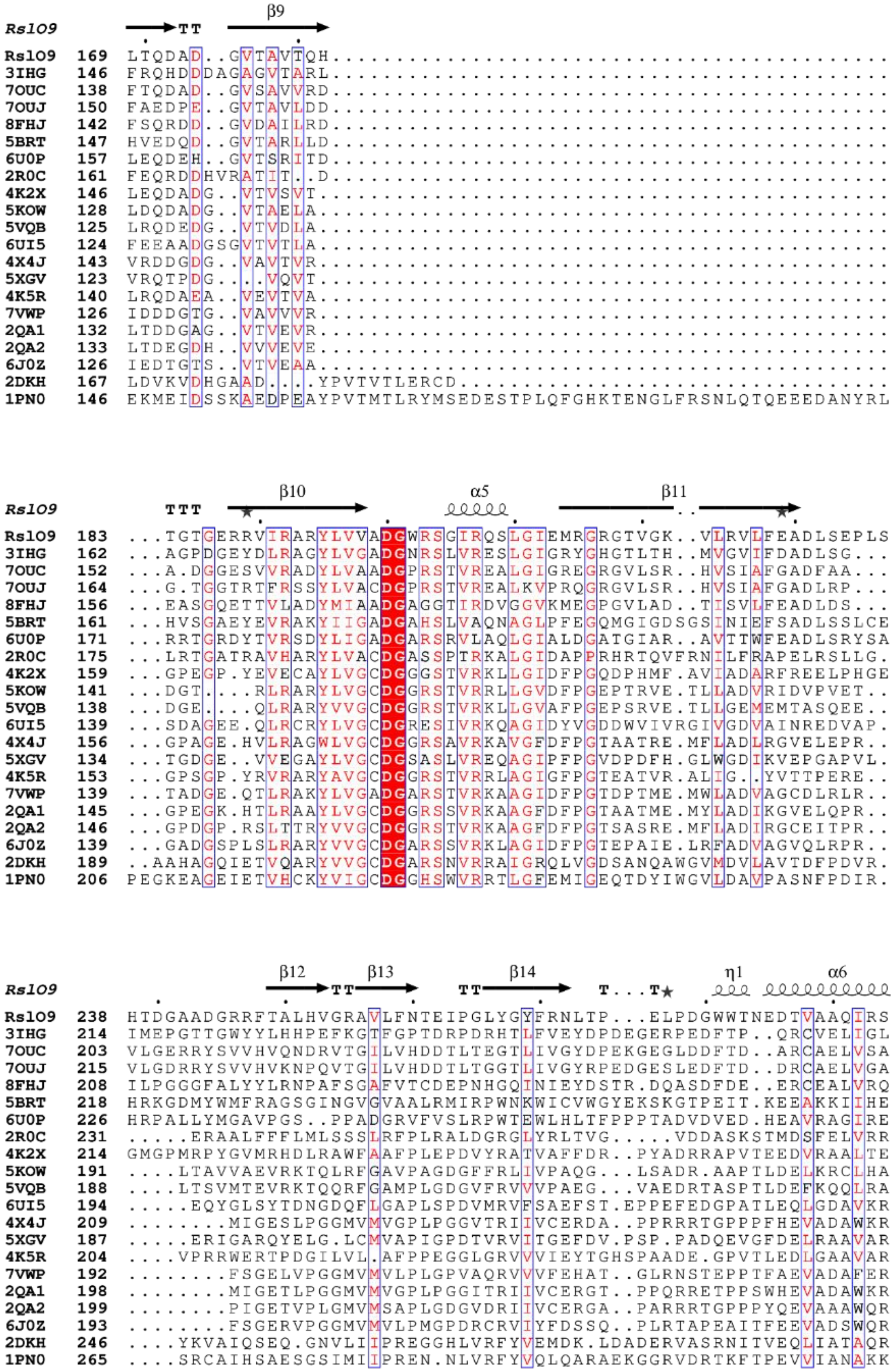
β9
Rs1O9 TT
Rs1O9 169 LTQDAD..GVTAVTQH..
3IHG 146 FRQHDDDAGAGVTARL..
7OUC 138 FTQDAD..GVSAVVRD..
7OUJ 150 FAEDPE..GVTAVLDD..
8FHJ 142 FSQRDD..GVDAILRD..
5BRT 147 HVEDQD..GVTARLLD..
6U0P 157 LEQDEH..GVTSRITD..
2R0C 161 FEQRDDHVRATIT..D..
4K2X 146 LEQDADG..VTVSVT...
5KOW 128 LDQDADG..VTAELA...
5VQB 125 LRQDEDG..VTVDLA...
6UI5 124 FEEAADGSGVTVTLA...
4X4J 143 VRDDGDG..VAVTVR...
5XGV 123 VRQTPDG....VQVT...
4K5R 140 LRQDAEA..VEVTVA...
7VWP 126 IDDDGTG..VAVVVR...
2QA1 132 LTDDGAG..VTVEVR...
2QA2 133 LTDEGDH..VVVEVE...
6J0Z 126 IEDTGTS..VTVEAA...
2DKH 167 LDVKVDHGAAD....YPVTVTLERCD..................................
1PN0 146 EKMEIDSSKAEDPEAYPVTMTLRYMSEDESTPLQFGHKTENGLFRSNLQTQEEEDANYRL
β10 α5 β11
Rs1O9 TTT
Rs1O9 183 ...TGTGERRVIRARYLVVADGWRSGIRQSLGIEMRGRGTVGK..VLRVLFEADLSEPLS
3IHG 162 ...AGPDGEYDLRAGYLVGADGNRSLVRESLGIGRYGHGTLTH..MVGVIFDADLSG...
7OUC 152 ...A.DGGESVVRADYLVAADGPRSTVREALGIGREGRGVLSR..HVSIAFGADFAA...
7OUJ 164 ...G.TGGTRTFRSSYLVACDGPRSTVREALKVPRQGRGVLSR..HVSIAFGADLRP...
8FHJ 156 ...EASGQETTVLADYMIAADGAGGTIRDVGGVKMEGPGVLAD..TISVLFEADLDS...
5BRT 161 ...HVSGAEYEVRAKYIIGADGAHSLVAQNAGLPFEGQMGIGDSGSINIEFSADLSSLCE
6U0P 171 ...RRTGRDYTVRSDYLIGADGARSRVLAQLGIALDGATGIAR..AVTTWFEADLSRYSA
2R0C 175 ...LRTGATRAVHARYLVACDGASSPTRKALGIDAPPRHRTQVFRNILFRAPELRSLLG.
4K2X 159 ...GPEGP.YEVECAYLVGCDGGGSTVRKLLGIDFPGQDPHMF.AVIADARFREELPHGE
5KOW 141 ...DGT....RLRARYLVGCDGGRSTVRRLLGVDFPGEPTRVE.TLLADVRIDVPVET..
5VQB 138 ...DGE....QLRARYVVGCDGGRSTVRKLLGVAFPGEPSRVE.TLLGEMEMTASQEE..
6UI5 139 ...SDAGEE.QLRCRYLVGCDGRESIVRKQAGIDYVGDDWVIVRGIVGDVAINREDVAP.
4X4J 156 ...GPAGE.HVLRAGWLVGCDGGRSAVRKAVGFDFPGTAATRE.MFLADLRGVELEPR..
5XGV 134 ...TGDGE..VVEGAYLVGCDGSASLVREQAGIPFPGVDPDFH.GLWGDIKVEPGAPVL.
4K5R 153 ...GPSGP.YRVRARYAVGCDGGRSTVRRLAGIGFPGTEATVR.ALIG..YVTTPERE..
7VWP 139 ...TADGE.QTLRAKYLVGADGARSTVRKAAGIDFPGTDPTME.MWLADVAGCDLRLR..
2QA1 145 ...GPEGK.HTLRAAYLVGCDGGRSSVRKAAGFDFPGTAATME.MYLADIKGVELQPR..
2QA2 146 ...GPDGP.RSLTTRYVVGCDGGRSTVRKAAGFDFPGTSASRE.MFLADIRGCEITPR..
6J0Z 139 ...GADGSPLSLRARYVVGCDGARSSVRKLAGIDFPGTEPAIE.LRFADVAGVQLRPR..
2DKH 189 ..AAHAGQIETVQARYVVGCDGARSNVRRAIGRQLVGDSANQAWGVMDVLAVTDFPDVR.
1PN0 206 PEGKEAGEIETVHCKYVIGCDGGHSWVRRTLGFEMIGEQTDYIWGVLDAVPASNFPDIR.
β12 β13 β14 η1 α6
Rs1O9 TT TT T...T
Rs1O9 238 HTDGAADGRRFTALHVGRAVLFNTEIPGLYGYFRNLTP...ELPDGWWTNEDTVAAQIRS
3IHG 214 IMEPGTTGWYYLHHPEFKGTFGPTDRPDRHTLFVEYDPDEGERPEDFTP..QRCVELIGL
7OUC 203 VLGERRYSVVHVQNDRVTGILVHDDTLTEGTLIVGYDPEKGEGLDDFTD..ARCAELVSA
7OUJ 215 VLGDRRYSVVHVKNPQVTGILVHDDTLTGGTLIVGYRPEDGESLEDFTD..DRCAELVGA
8FHJ 208 ILPGGGFALYYLRNPAFSGAFVTCDEPNHGQINIEYDSTR.DQASDFDE..ERCEALVRQ
5BRT 218 HRKGDMYWMFRAGSGINGVGVAALRMIRPWNKWICVWGYEKSKGTPEIT.KEEAKKIIHE
6U0P 226 HRPALLYMGAVPGS..PPADGRVFVSLRPWTEWLHLTFPPPTADVDVED.HEAVRAGIRE
2R0C 231 .....ERAALFFFLMLSSSLRFPLRALDGRGLYRLTVG.....VDDASKSTMDSFELVRR
4K2X 214 GMGPMRPYGVMRHDLRAWFAAFPLEPDVYRATVAFFDR..PYADRRAPVTEEDVRAALTE
5KOW 191 ....LTAVVAEVRKTQLRFGAVPAGDGFFRLIVPAQG...LSADR.AAPTLDELKRCLHA
5VQB 188 ....LTSVMTEVRKTQQRFGAMPLGDGVFRVVVPAEG...VAEDRTASPTLDEFKQQLRA
6UI5 194 .....EQYGLSYTDNGDQFLGAPLSPDVMRVFSAEFST.EPPEFEDGPATLEQLGDAVKR
4X4J 209 ........MIGESLPGGMVMVGPLPGGVTRIIVCERDA..PPRRRTGPPPFHEVADAWKR
5XGV 187 .....ERIGARQYELG.LCMVAPIGPDTVRVITGEFDV.PSP.PADQEVGFDELRAAVAR
4K5R 204 ....VPRRWERTPDGILVL.AFPPEGGLGRVVVIEYTGHSPAADE.GPVTLEDLGAAVAR
7VWP 192 ........FSGELVPGGMVMVLPLGPVAQRVVVFEHAT..GLRNSTEPPTFAEVADAFER
2QA1 198 ........MIGETLPGGMVMVGPLPGGITRIIVCERGT..PPQRRETPPSWHEVADAWKR
2QA2 199 ........PIGETVPLGMVMSAPLGDGVDRIIVCERGA..PARRRTGPPPYQEVAAAWQR
6J0Z 193 ........FSGERVPGGMVMVLPMGPDRCRVIYFDSSQ..PLRTAPEAITFEEVADSWQR
2DKH 246 ....YKVAIQSEQ.GNVLIIPREGGHLVRFYVEMDK.LDADERVASRNITVEQLIATAQR
1PN0 265 ....SRCAIHSAESGSIMIIPREN.NLVRFYVQLQARAEKGGRVDRTKFTPEVVIANAKK

```
                 β15           β16              β17  η2  β18   TT
Rs109
Rs109  295  DLGIPDIPLKIE.EISETEISCGVAERFRE............GRALLVGDAAHVMPPTGG
3IHG   272  ALDAPEVKPELV.DIQGWEMAARIAERWRE............GRVFLAGDAAKVTPPTGG
7OUC   261  AIGSDDVAVTIR.SRFPWDMAELVADAFVS............DRVLVAGDAAHQIPPTGG
7OUJ   273  AVGAPGVEVTIR.SRFPWDMAEQVAESFVH............GRVLLAGDAAHVVPPTGG
8FHJ   265  SLGVADLAVKIL.DIRPWQMAALLADRMSF............GRVFLAGDCAHITPPVGG
5BRT   277  IIGTDEIPVEVG.PISTWTINQQYAVRNTS............GRVFCMGDAVHRHTPMGG
6U0P   283  SIGDPTVDVTIK.NVSAWEVNSAVAPRYAS............GRVFCVGDAVHQNPPTNG
2R0C   281  AVAFDTEIEVLS..DSEWHLTHRVADSFSA............GRVFLTGDAAHTLSPSGG
4K2X   272  VAGSDFGMHDVR.WLSRLTDTSRQAERYRD............GRVLLAGDACHIHLPAGG
5KOW   243  TAGTDFGVHSPR.WLSRFGDATRLAERYRT............GRVLLAGDAAHIHPPTGG
5VQB   241  HAGTDFGVHSPR.WLSRFGDATRQAERYRV............DRVFLAGDAAHIHPPTGG
6UI5   248  LTGKELKATEAH.WLQHYSIVTRNAEQYRK............GRVFIAGDAAHVHYPYNG
4X4J   259  ITGIDISAAEPV.WLSAFGDATRQVTEYRR............GRVLLAGDAAHVHLPAGG
5XGV   239  IAGVELDGVPG..WLSRWTATSRQAERYRE............GRILLAGDAAHTLFPLGG
4K5R   258  VRGTPLTLTEPVSWLSRFGDASRQAKRYRS............GRVLLAGDAAHVHFPIGG
7VWP   242  LTGEDIRGGKPL.WVSWFTDSSRQAAEYRR............GRILLAGDAAHIHMPIGG
2QA1   248  LTGDDIAHAEPV.WVSAFGNATRQVTEYRR............GRVILAGDSAHIHLPAGG
2QA2   249  LTGQDISHGEPV.WVSAFGDPARQVSAYRR............GRVLLAGDSAHVHLPAGG
6J0Z   243  LTGEDISGATPL.WVSSATDVSRQAAQYRK............GRVFLAGDAAHIHLPIGA
2DKH   300  VLHPYKLEVKNVPWWSVYEIGQRICAKYDDVVDAVATPDSPLPRVFIAGDACHTHSPKAG
1PN0   320  IFHPYTFDVQQLDWFTAYHIGQRVTEKFSKDE..........RVFIAGDACHTHSPKAG

                       α7                  η3         α8
Rs109
Rs109  342  MGGNTAYLDGLYLGWKLAAVLRGTAGEALLDSHDAERRPYAEELVEQQFANLVDRIS...
3IHG   319  MSGNAAVADGFDLAWKLAAVLQGQAGAGLLDTYEDERKVAAELVVAEALAIYAQRMA...
7OUC   308  YGANTGIADAFNLSWKLAHVLAGTAGRALLDTYDEERRPVGLYTARQGSLQLAVRSR...
7OUJ   320  YGANTGIADAHNLAWKLALVAAGVAGPGLVETYDAERRPVAVYTAEQGSLQLALRSG...
8FHJ   312  LGGQTAIQDAADLAWKLALVVKGQAAPTLLDSYEIERRPVARIAIARSIANYVERLL...
5BRT   324  LGLNTSVQDAYNLAWKLALVLKGTAAPTLLDSYDAERSPVAKQIVERAFKSLSTFPPVFE
6U0P   330  LGLNSAVADSFNLCWKLKLALEGLAGPGLLDTYHDERQPVGRQIVDRAFRSMVDLIGIPQ
2R0C   327  FGMNTGIGSAADLGWKLAATLRGWAGPGLLATYEEERRPVAITSLEEANVNLRRTM..DR
4K2X   319  QGLNLGFQDAVNLGWKLGATIAGTAPPELLDTYEAERRPIAAGVLRNTRAQAVLIDP...
5KOW   290  QGLNLGIQDAFNLGWKLAAAIGGWAPPDLLDSYHDERHPVAAEVLDNTRAQMTLLSL...
5VQB   288  QGLNLGIQDAFNLGWKLAAEVDGWAPEGLLDTYHAERHPVATEVLDNTRAQIQLMST...
6UI5   295  QGLGTAIGDAVNLGWKIAAEVHGWAPADLLDSYHVERHLAGRLACMNIQAQLALLYP...
4X4J   306  QGMNAGIQDAVNLGWKLAAVVRGTARADLLDTYHGERHPVGVRLLMNTRAQGLLFLN...
5XGV   285  QALGTGIEDAVNLGWKLAATVQGWAPPSLLDSYHEERHAAGARACASTRAQTTIMRS...
4K5R   306  QGLNTGLQDAVNLGWKLAARVRGWGSEELLDTYHDERHPVAERVLLNTRAQLALMRP...
7VWP   289  QGMSAGIQDAVNLGWKLAAEIHGHAPEGLLDTYHTERHPVDGRVVMNTLAQRWLYLG...
2QA1   295  QGMNTSIQDAVNLGWKLGAVVNGTATEELLDSYHSERHAVGKRLLMNTQAQGLLFLS...
2QA2   296  QGMNVSVQDSVNLGWKLAAVVSGRAPAGLLDTYHEERHPVGRRLLMNTQAQGMLFLS...
6J0Z   290  QGMSAGVQDAVNLGWKLALDISGRAPQGLLDTYHSERHPVGQRILTNTLAQRILYLG...
2DKH   360  QGMNFSMQDSFNLGWKLAAVLRKQCAPELLHTYSSERQVVAQQLIDFDREWAKMFS....
1PN0   369  QGMNTSMMDTYNLGWKLGLVLTGRAKRDILKTYEEERQPFAQALIDFDHQFSRLFSG...

                                                                α9
Rs109
Rs109  399  ................................P.ELADESLAVALPPPVVAFG
3IHG   376  ................................P.HMA.EVWDKSVGYPETLLG
7OUC   365  ................................T..ATEEQREAAHDAMRVTMG
7OUJ   377  ................................T..ATPEQQAAVADAVTVTSG
8FHJ   369  ................................PDRQDIRIREDEYGLLETAMG
5BRT   384  ALSLPPAPTESEMAEALVRLKDASEEGAKRRAAL.....RKAMDATIIGLGGGHGVELNQ
6U0P   390  ALGFTEGQSPEEQWRLLDTLHEDTEEARQRRAALAAAT..AAIHGQANAHGVELGYRYRT
2R0C   385  ELPPGLHDDGPRGERIRAAVAEKLERSGARR.................EFDAPGIHFG
4K2X   376  ................................D.PRYEGLRELMIELLHVPET
5KOW   347  ................................D.PGPRAVRRLMAELVE.FPD
5VQB   345  ................................E.PGPQAVRRLMAELVE.FEN
6UI5   352  ................................R.PLARYMREMMGEFLKFDEV
4X4J   363  ................................G.AEMQPLRDVLAELTGYPDV
5XGV   342  ................................L.ARVGELRALLTELAG.LEE
4K5R   363  ................................DEQHTTPLRGFVEELLGTDEV
7VWP   346  ................................G.EAMQPLRELLGELVRYPDV
2QA1   352  ................................G.PEVQPLRDVLTELIQ.YGE
2QA2   353  ................................G.DEMQPLRDVLSELIR.YDE
6J0Z   347  ................................G.DEITPMREVLAELMGSHVS
2DKH   416  .............................DPAKEGGQGGVDPKEFQKYFEQHG
1PN0   426  ............................RPAKDVADEMGVSMDVFKEAFVKGN
```

```
                    β19        β20  η4
Rs109                   TT                  TT       TT
Rs109  419  YRFPKGAVLLEPDDDGELFE...DPSRPTGRPGSHAPYVPLTRR...DGSATSTTALFGH
3IHG   395  FRYRSSAVLAT.DDDPARVE...NPLTPSGRPGFRGPHVLVSR....HGERLSTVDLFGD
7OUC   384  QAYPSGAFVADAGADPLPLTS..DPRTLRGEPGTRAPYVVLER....DGAPLSTLDLFGD
7OUJ   396  QAYRSTAVVGEPDGADLPVAS..DPRELRGAPGTRAPYVELLR....GGETVSTLDLFGR
8FHJ   390  YRYRSDAIIADEFDDGACVE...DPLRPSGAPGTRLAHVWLRR....GEETISSHDLIGR
5BRT   439  RYVSRAVFPDGTPDPGFVRDQE.FFYQASTRPGAHLPHVWLTE...NQRRISTLDLCGKG
6U0P   448  GALVPDGTPEPADERDP.....ELYYRATTWPGARLPHAWLEN...GRHRCSTLDVTGRG
2R0C   426  HTYRSSIVCGEPETEVATGG.....WRPSARPGARAPHAWLTP......TTSTLDLFGRG
4K2X   396  NRYLAGLISALDVRYP........MAGEHPLLGRRVPDLPLV...TEDG.TRQLSTYFHA
5KOW   366  VNRHLIEKITAIAVRYD.......LGDGHDLVGRRLRDIPLT....EG....RL.YERMR
5VQB   364  VNRYLIEKITAISVRYD.......VGEGHELLGRRMRDLALK....HG....RL.YERMH
6UI5   372  NVFLAEIVTNLGPAVPIAYEGVPEPVEGDRLLGRRLPKVQIK....TADGDMGVAETLQS
4X4J   383  ARHLAAMVSGLEIAYDVG.......GGSHPWLGRRLPRLELD....RGGRPSSTAELLRP
5XGV   361  VNAYLVRMVGGID...................GSRLPDVPLV....TAEGETSVYRLLEA
4K5R   384  NRYFTGMITGTDVRYATFAPAA..PARPHPWAGRFAGGLVLS...GPSGEPVPVAELLRS
7VWP   366  QEHLVGMVTGLDIRYDVG.......AGEHPLLGRRIPNQELVG.EFDGSGKSTTFEQLHR
2QA1   371  VARHLAGMVSGLEITYDVG......TGSHPLLGKRMPALELT.....TATRETSSTELLH
2QA2   372  VSRHLAGMVSGLDIRYEVD......GGDHPLLGMRMPHQELV.....RAHGKTSTTELLH
6J0Z   367  VQRHLAGMVTGLDIRHDVG......EGDHPLLGRRLPDRELV....VDGEKIPFYSLLRP
2DKH   440  RFTAGVGTHYAPSLLTG.QA.KHQALASGFTVGMRFHSAPVVRVCDAKPVQLGHCGKADG
1PN0   451  EFASGTAINYDENLVTDKKS.SKQELAKNCVVGTRFKSQPVVRHSEGLWMHFGDRLVTDG
```

```
               β21       α10            β22           β23   α11
Rs109                                         TT
Rs109  473  A.FVLLTGPDGAAWAEGALASADALGVAVQVHRVGPG...........SELLDAEGAFST
3IHG   447  G.WTLLAGELGADWVAAAEAVSAELGVPVRAYRVGAG............LTDPESAVSE
7OUC   438  G.FVLLVGADGGSWAEAAGEAAAGLGVGIAFHRVAP..DAGE.....G.RPVDVHGRWAE
7OUJ   450  D.FVLLTGEHGREWISAAVSASAGLGLKITARRVVPGTDAGA.....G.TLADPDGDWSE
8FHJ   443  D.FMLFTGPDGGDWIEAARRIALRSKAPLGVCRLGFD.............VDDPEGLFLP
5BRT   495  RFTLLTGLSGAAWKHEAEQVSQSLGIELKVCVIGPG............QEFVDTYGEYAK
6U0P   500  RFTLLTGPGGEPWRDAARDAALDTGVEVAVLPIGAG...........GGPRDPYGTWAEL
2R0C   475  .FVLLSFGTTDGVEAVTRAFADRHVPLETVTCHAPEIHA.....................
4K2X   444  ARGVLLTLGCDQPLADEAAAWKDRVDLVAAEGVADPGSA.....................
5KOW   410  GGRG.LLDRTG....RLSVSGWSDRVDHLA.DPGAALD......................
5VQB   408  EGRG.LLDQTG....RLSVAGWEDRVDHVV.EVSEELD......................
6UI5   428  GRGVLLDLSGDAS.AQEESGWADRVDVVRAQPVPDLPG......................
4X4J   432  ARGLLLDFAGNAALRDRAAPWAGRIDVVTARPAAGRVPG.....................
5XGV   398  GRG.VLLDLGAGLP.AVRHP....QVTYVRAEPTNRLDAT....................
4K5R   439  ARPLLLDLAGRADLREATRPWSDRVSVVAGEATVEPP.......................
7VWP   418  GRGVLFAFGDDTAGPQAATGWTDRVDVVRATPHTADPDDP....................
2QA1   420  TARGVLLDLADNPRLRARAAAWSDRVDIVTAVPGEVSATSG...................
2QA2   421  PARGVLLDIADDAEVREAATGWSDRVDIVTASLHDAPPQGP...................
6J0Z   417  GRAVLL.ELGGDRGLRTAAAGWADRVDLVAAEFDGCEAP.....................
2DKH   498  RWRLYAFAAQNDLAQPESGLLALCRFLEGDAASPLRRFTPAGQDIDSIFDLRAVFPQAYT
1PN0   510  RFRIIVFAGKATDATQMSRIKKFAAYLD.SENSVISRYTPKGADRNSRIDVITIHSCHRD
```

```
                    β24     β25        α12
Rs109                  TT
Rs109  521  AYG.IGADGAALVRPDRFVAWRCTGTHP..DPAAEIERALRHVLHRPAH...........
3IHG   493  RYG.IGKAGASLVRPDGIVAWRTDEAAA..DAAQTLEGVLRRVLDR..............
7OUC   489  AYG.VGAAGAVLVRPDGIVAWRSRDGMPGGAGGRALTAALRTVLAR..............
7OUJ   503  RYGGLRPEGAVLVRPDGVVAWRSPGADPGGEESAVLAAVLRSVLARESRTGGK.......
8FHJ   489  RLR.ISPEGALLVRPDGYIAWRSRGRSP..DPFATLEASFARVRGFDTGQSSGSHAAAFA
5BRT   543  ISE.IGESGALLVRPDMFIAFRAKDASRE.........GLEQLNVAVKSILGRA......
6U0P   549  RE..VEESGAVLVRPDGHVAWRARDHGHAKELPEVMARVLHQPDPAARRTGGPGA.....
2R0C   513  ....LYERAHVLVRPDGHVAWRGD......HLPAELGGLVDKVRGAA.............
4K2X   483  ....VDGLTALLVRPDGYICWTAAPETGTDGLTDALRTWFGPPAM...............
5KOW   442  .......VPAALLRPDGHVAWVGE...DQDDLLAHLPRWFGAAT................
5VQB   440  .......VPAVLLRPDGHVVWAGE...DQQELLTRMPAWFGAATAG..............
6UI5   465  .........TLLLRPDGCVAWHDGGGWGQDELRTALRTWFGAPTG...............
4X4J   471  ......ATTAVLVRPDGHVAWAAPGTH..ADLPMALERWFGPAPR...............
5XGV   432  .........AVLLRPDGVVAWRAP....QDGLEAALETWFGPAA................
4K5R   476  .......AQALLVRPDGYVAWAGSPAATADELRASLARWFGPPANREPVGHQERAGRRGR
7VWP   458  ....FHGLDAVLVRPDGYVAWVAPAGAGAAGLDEALSRWFGPSR................
2QA1   461  ....LRDTTAVLIRPDGHVAWAAPGSH..HDLPMALERWFGAPLTG..............
2QA2   462  ....LSDARAVLVRPDGYVAWISPGSR..AGLTEALDRWFGPAR................
6J0Z   455  .......VDGILVRPDGYVAWVAALGAGADGLTTALDRWFGPTA................
2DKH   558  EVALETLPALLLPPKGQLGMIDYEKVFSPDLKNAGQDIFELRGIDRQQGALVVVRPDQYV
1PN0   569  DIEMHDFPAPALHPKWQY...DFIYADCDSWHHPHPKSYQAWGVDETKGAVVVVRPDGYT
```

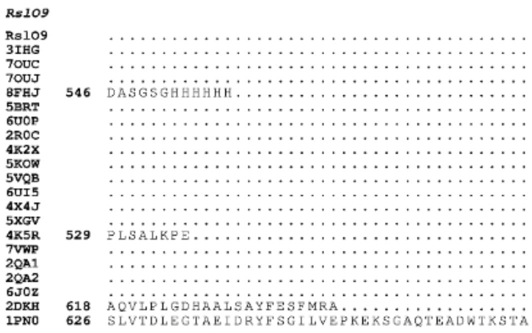

Figure S16: Multiple Sequence Alignment of RslO9 and characterized homologs. Multiple sequence alignment (MSA) of RslO9 and characterized homologs (abbreviated with their PDB ID) generated with MEGA X and manual adjustment based on structural alignment of the crystal structures of the enzymes, secondary structure elements of RslO9 are shown on top of the alignment, conserved amino acids are highlighted in red. Representation of the secondary structure above the alignment was generated via the ESPript 3.0 online server[3].

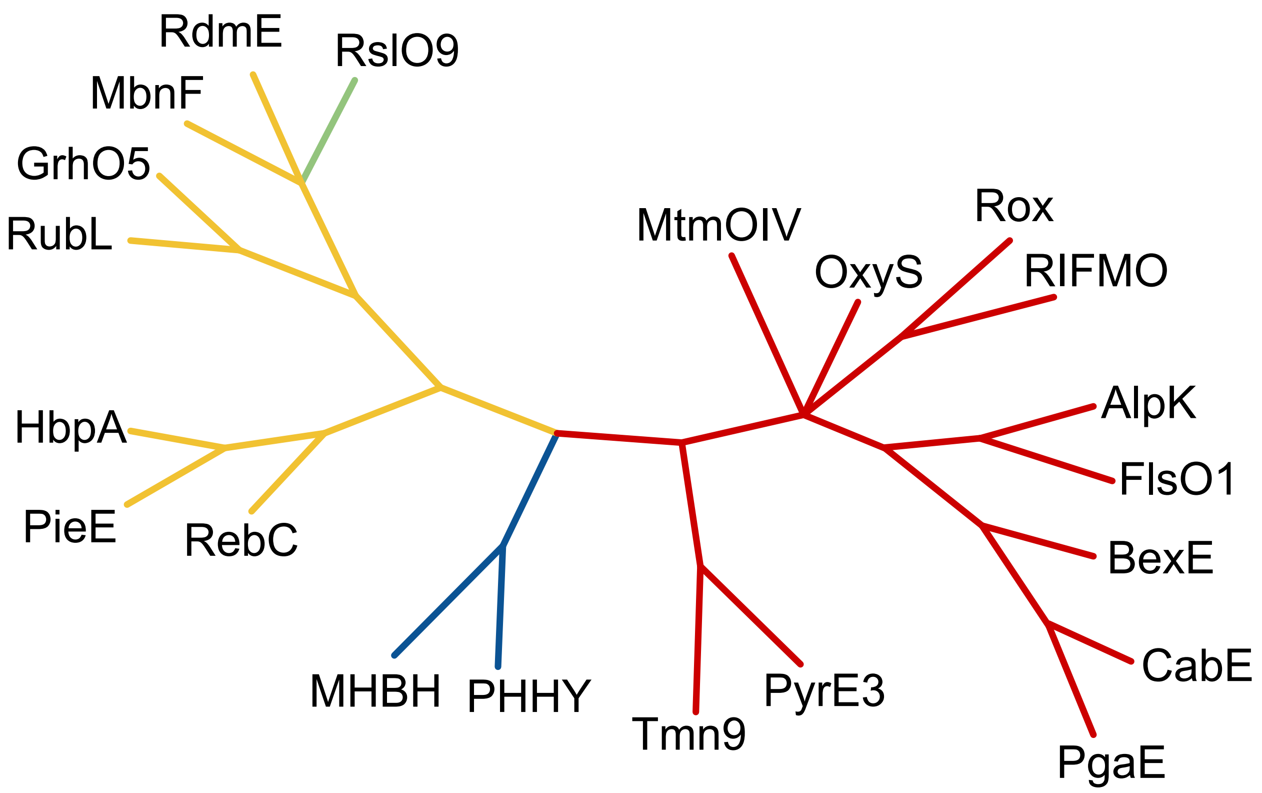

Figure S17: Phylogenetic tree of RslO9 and characterized homologs. Phylogenetic tree of RslO9 and the closest related and investigated FPMOs. GrhO5-like group I is shown in yellow, MtmOIV-like group II is shown in red and PHHY-like group is shown in blue, RslO9 is highlighted in green.

Supplementary Note 3: Phylogenetic relation of RslO9 to other investigated FPMOs

Previously, we tentatively categorized group A FPMOs into 3 different subtypes namely GrhO5-like type I, MtmOIV-like type II or the distantly related PHHY-like group (adjusted tree representation in Figure S17)[4]. In the GrhO5-type group I, the OUT-conformation of the FAD cofactor is found in the resting state and stabilized by π-stacking interactions of aromatic Trp side chains with the isoalloxazine moiety, whereas MtmOIV-like enzymes stabilize the IN conformation. RslO9 is closely related to members from the GrhO5-type group I but does not contain the characteristic Trp residue, possibly explaining the missing stabilization of the OUT conformation and the lack of a charge-transfer (CT) interaction; accordingly, unlike GrhO5, the UV-Vis spectrum of RslO9 did not exhibit a long-wavelength absorption resulting from a CT complex (>500 nm)[4]. Interestingly, a W222 side-chain was positioned in a loop at the surface of RslO9 next to the FAD-binding site, which is not found in the other enzymes. Interactions of the FAD in the "out" conformation could be possible, which would have to be further investigated.

When comparing the immediate surrounding of the isoalloxazine ring of the FAD in RslO9 with its closest homolog RdmE, most of the residues appear similar. The PPTGG-loop`s are identical and positioned in the same orientation. Below the FAD, the R45 of RdmE is exchanged to L72 in RslO9. Continuing this loop in RdmE a sequence of "A**A**GQ" follows while RslO9 contains "A**T**GQ". The whole loop and especially T74 reaches further into the cleft between FAD-binding domain and middle-domain of RslO9 than observed for RdmE. Additionally, a loop containing I72 of RdmE (not present in RslO9) occupies part of this space adding to the hydrophobic environment compared to RslO9. This is also evident from the lack of water molecules which are present in the RslO9 crystal structure. Although the structural similarity of GrhO5 and RslO9 is lower, the same trend can be seen here. The sequence of R45 is followed by "A**V**GY" in GrhO5 and with F73 and L112 also reaching into the same site, this region is hydrophobic compared to RslO9.

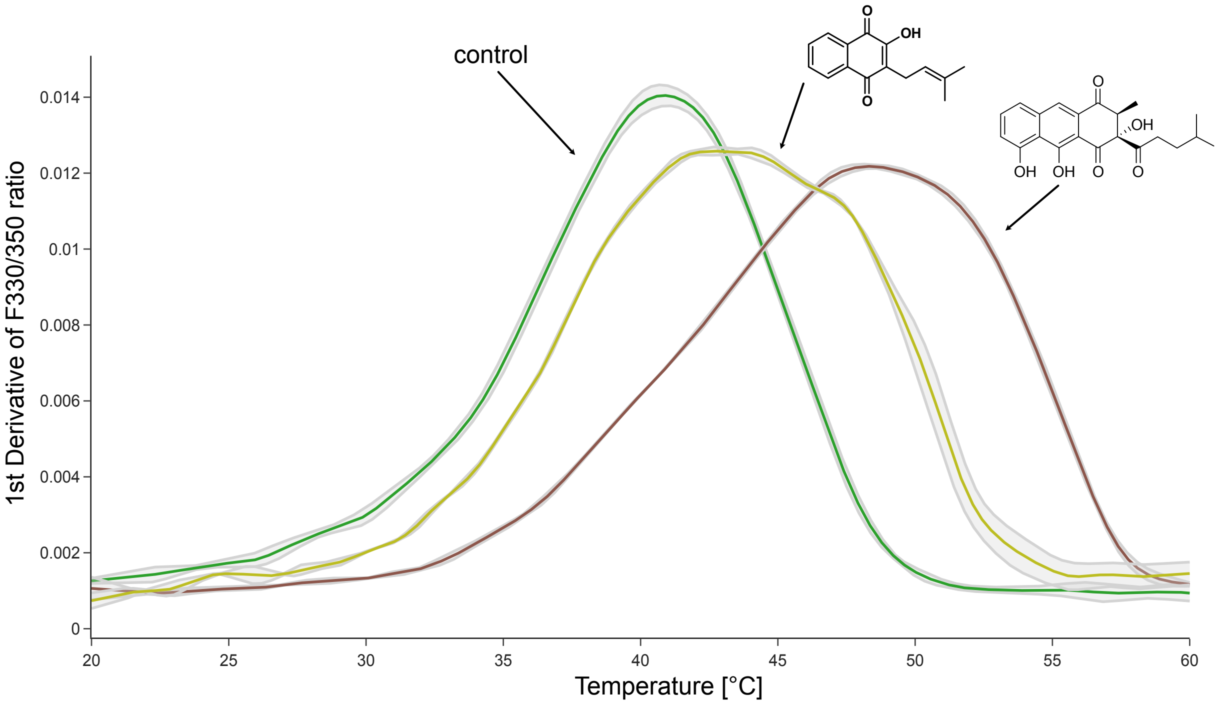

Figure S18: representative melting curves of RslO9. Representative melting curve of the 1st derivative of the ratio between 330/350 nm during thermal denaturation of RslO9 with natural substrate RSH-O9 (**5**) shown in brown, lapachol (**8**) in yellow and the solvent control in green. The error of the triplicate measurement is shown as a grey ribbon around the mean of the respective color.

Table S1: Comound overview. Overview of structures of all compounds tested in thermal shit assays of RslO9 with their compound ID used in this study.

Compound ID	Name	Structure
**5**	RSHO9	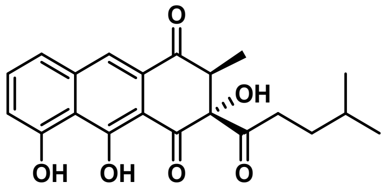
**7**	shikonin	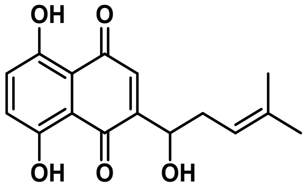
**8**	lapachol	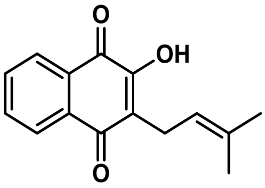
**9**	plumbagin	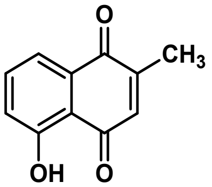
**10**	2-methyl-1,4-naphthoquinone	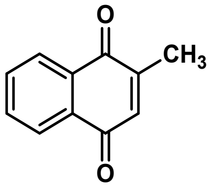
**11**	acequinocyl	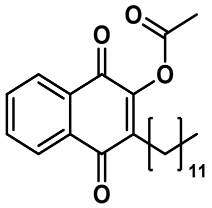
**12**	2-hydroxy-1,4-naphthoquinone	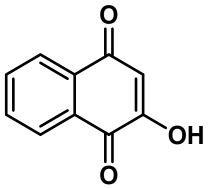
**13**	emodin	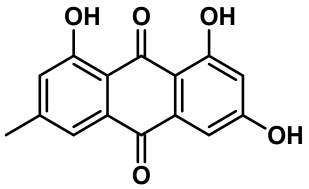

**14**	1,8-diyhdroxy-3-methylanthraquione	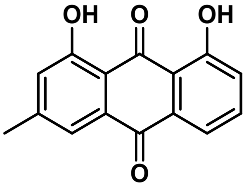
**15**	quinizarin	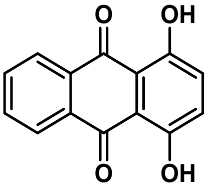
**21**	osthole	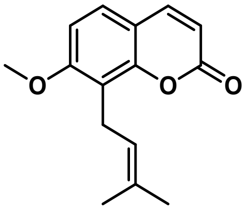
**22**	leucoquinizarin	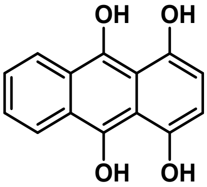

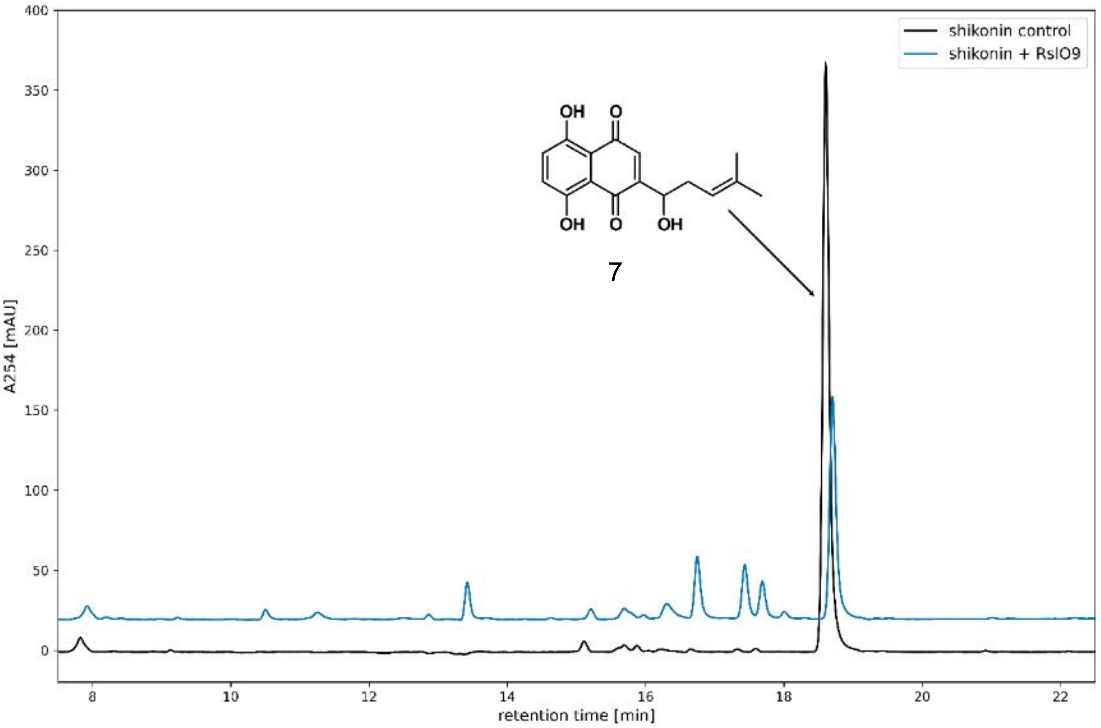

Figure S19: UV trace of RsIO9 reaction with 7. 254 nm UV traces of the LC/MS measurements from shikonin (**7**) control reaction and reaction with addition of RsIO9. The absorption at 254 nm on the y-axis is plotted against the retention time on the x-axis. UV-traces of control and enzyme condition are shifted to improve visibility.

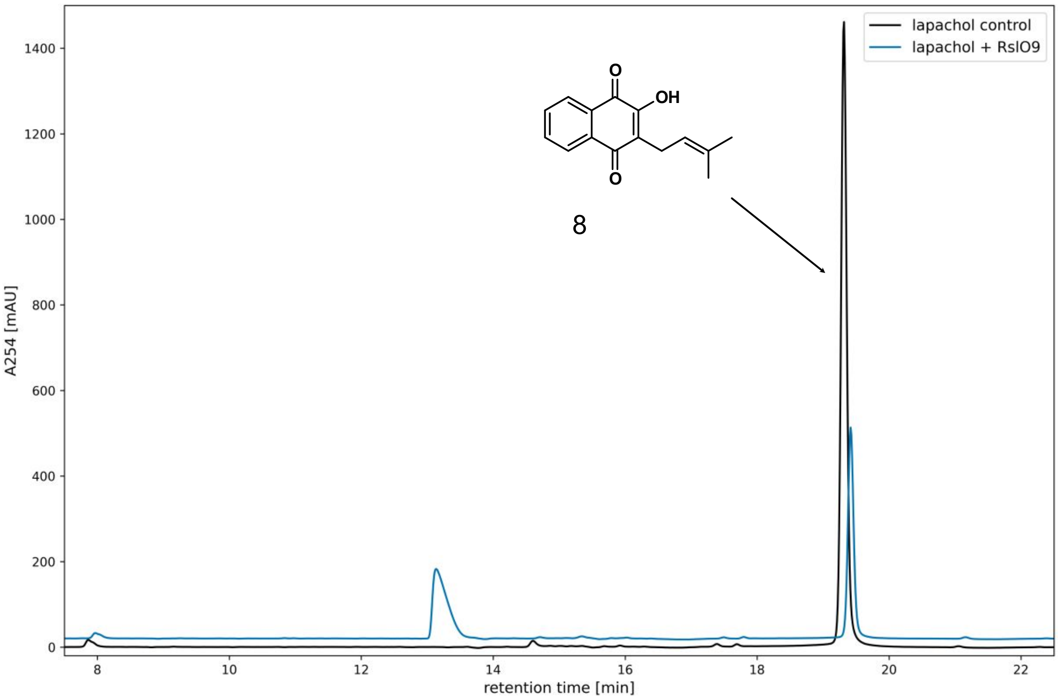

Figure S20: UV trace of RsIO9 reaction with 8. 254 nm UV traces of the LC/MS measurements from lapachol (**8**) control reaction and reaction with addition of RsIO9. The absorption at 254 nm on the y-axis is plotted against the retention time on the x-axis. UV-traces of control and enzyme condition are shifted to improve visibility.

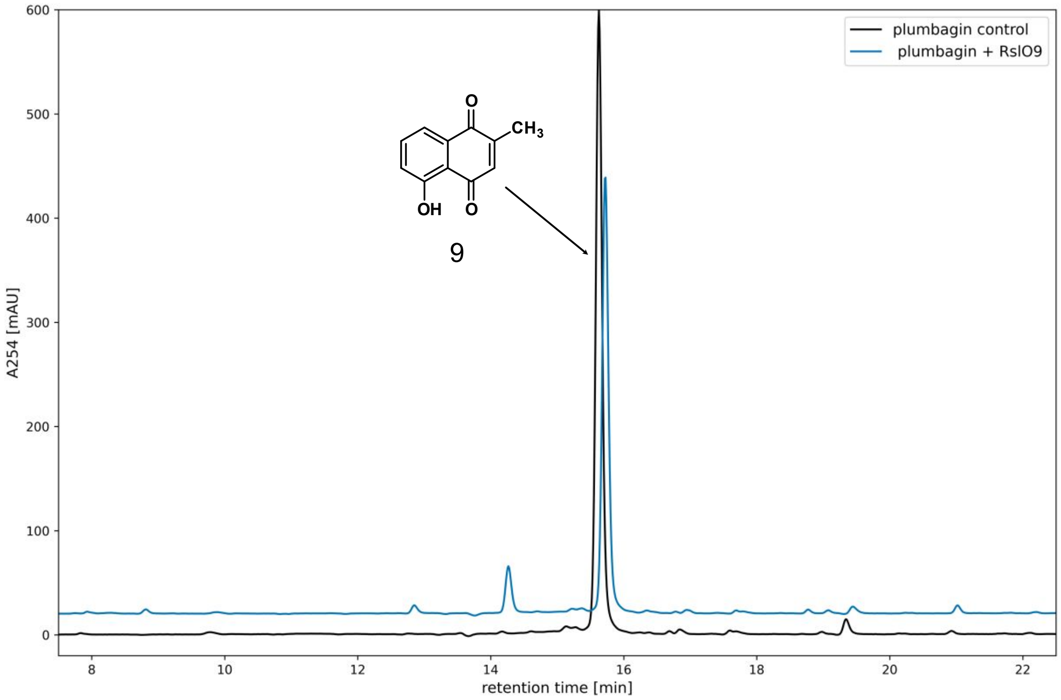

Figure S21: UV trace of RslO9 reaction with 9. 254 nm UV traces of the LC/MS measurements from plumbagin (**9**) control reaction and reaction with addition of RslO9. The absorption at 254 nm on the y-axis is plotted against the retention time on the x-axis. UV-traces of control and enzyme condition are shifted to improve visibility.

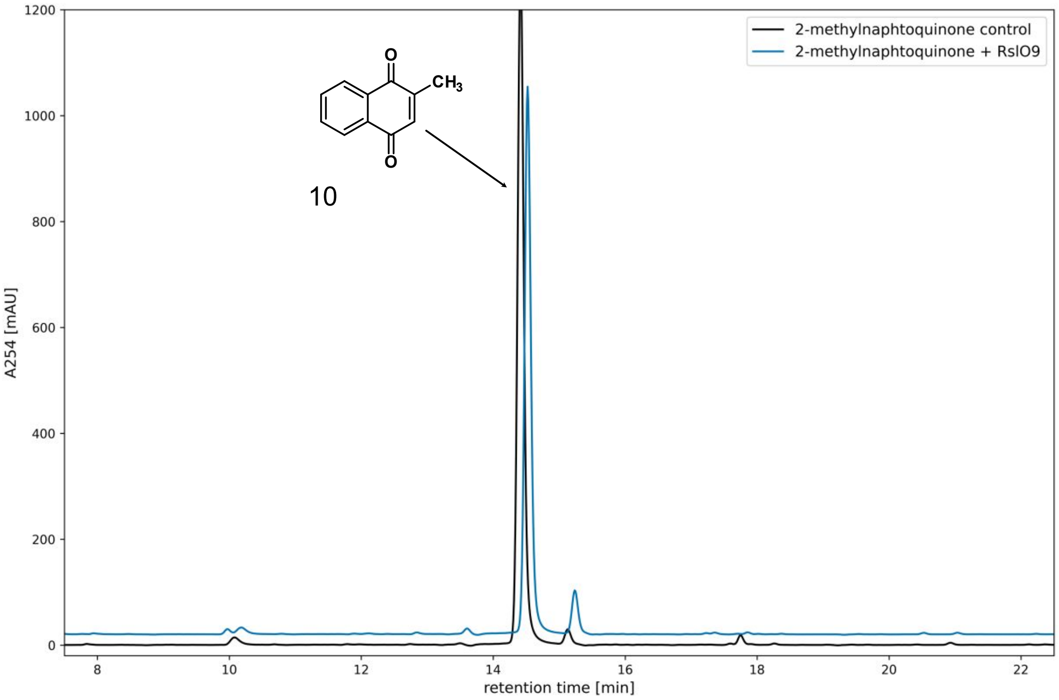

Figure S22: UV trace of RslO9 reaction with 10. 254 nm UV traces of the LC/MS measurements from 2-methyl naphthoquinone (10) control reaction and reaction with addition of RslO9. The absorption at 254 nm on the y-axis is plotted against the retention time on the x-axis. UV-traces of control and enzyme condition are shifted to improve visibility.

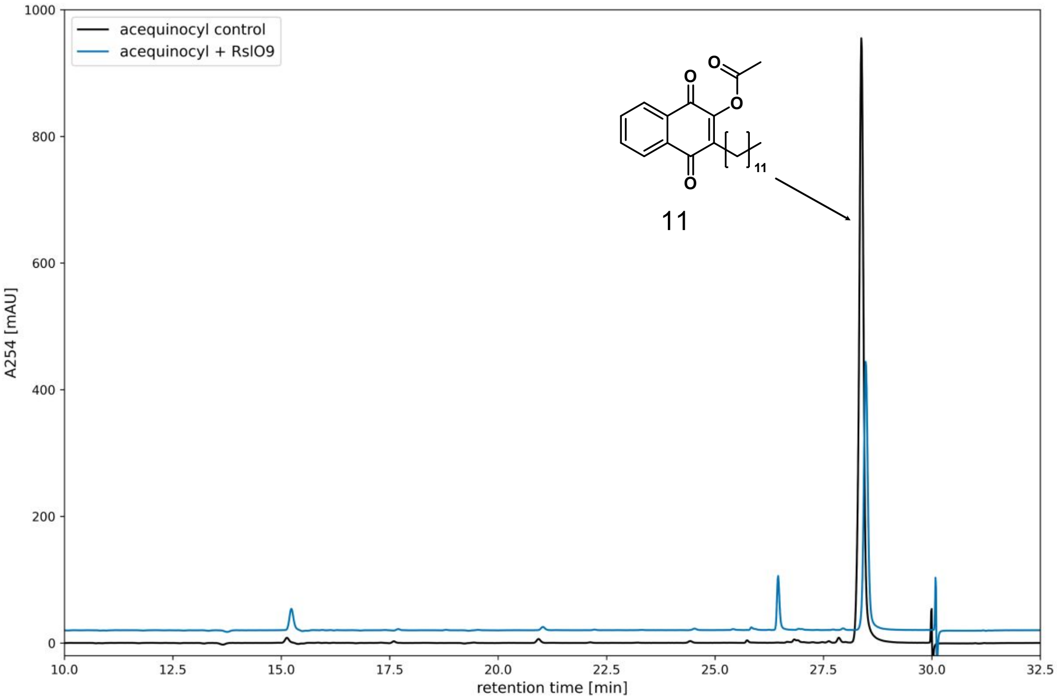

Figure S23: UV trace of RslO9 reaction with 11. 254 nm UV traces of the LC/MS measurements from acequinocyl (**11**) control reaction and reaction with addition of RslO9. The absorption at 254 nm on the y-axis is plotted against the retention time on the x-axis. UV-traces of control and enzyme condition are shifted to improve visibility.

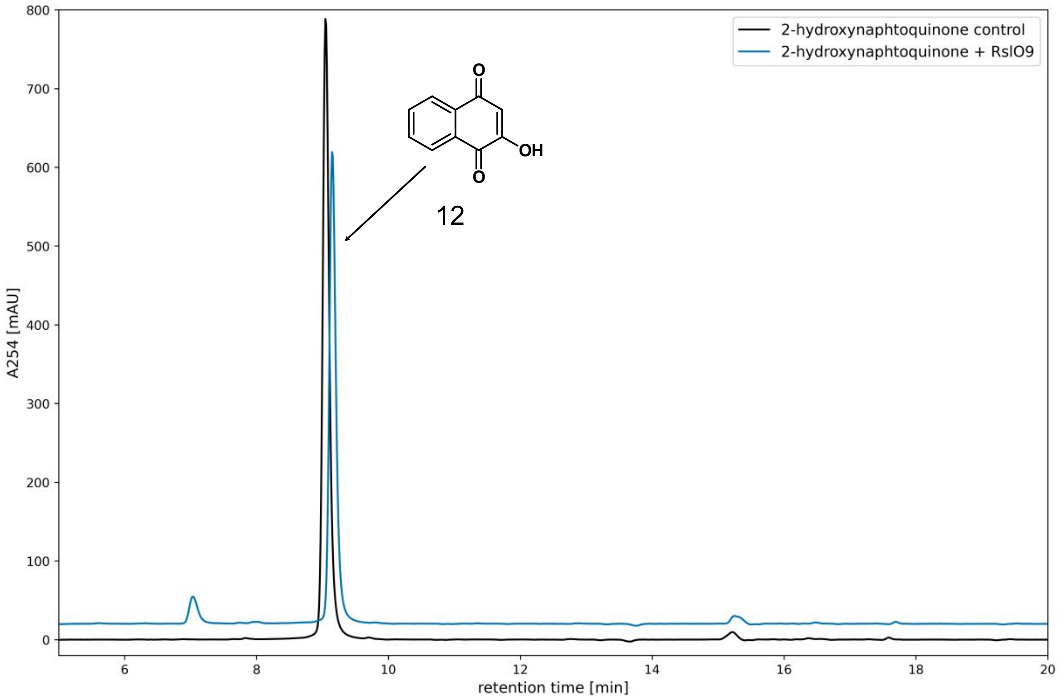

Figure S24: UV trace of RslO9 reaction with 12. 254 nm UV traces of the LC/MS measurements from 2-hydroxy napthoquinone (**12**) control reaction and reaction with addition of RslO9. The absorption at 254 nm on the y-axis is plotted against the retention time on the x-axis. UV-traces of control and enzyme condition are shifted to improve visibility.

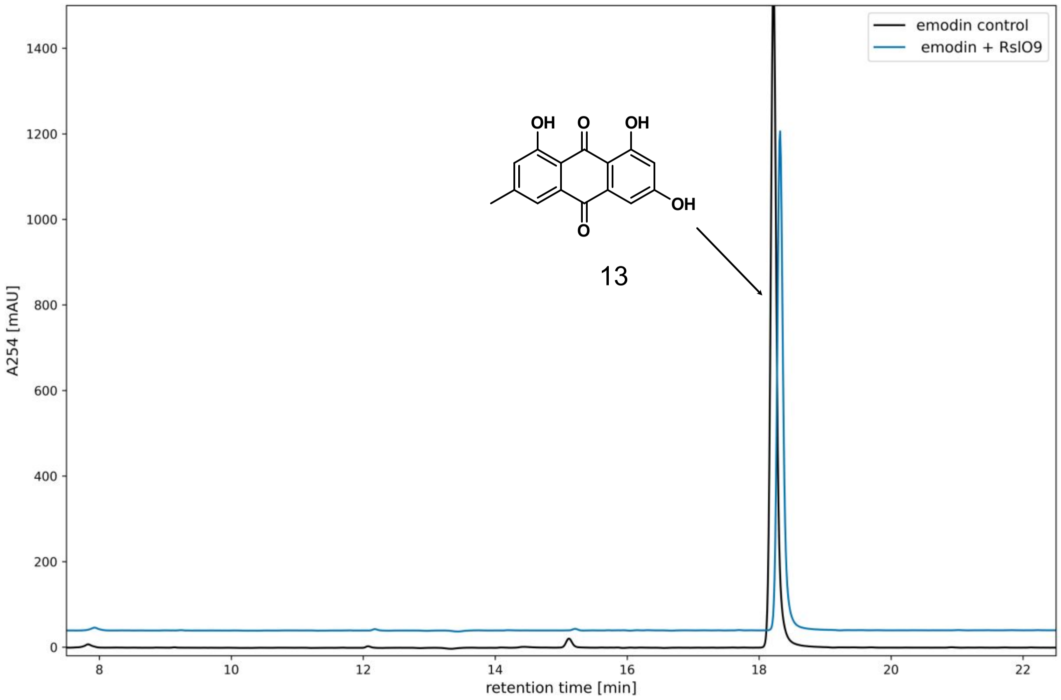

Figure S25: UV trace of RslO9 reaction with 13. 254 nm UV traces of the LC/MS measurements from emodin (**13**) control reaction and reaction with addition of RslO9. The absorption at 254 nm on the y-axis is plotted against the retention time on the x-axis. UV-traces of control and enzyme condition are shifted to improve visibility.

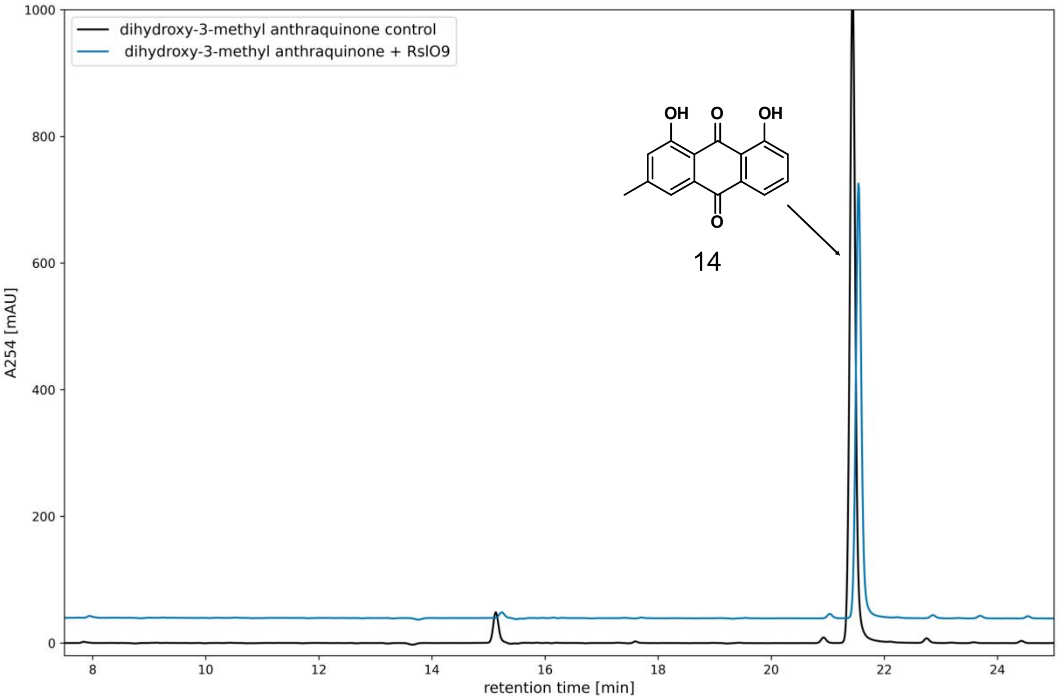

Figure S26: UV trace of RslO9 reaction with 14. 254 nm UV traces of the LC/MS measurements from 1,8-dihydroxy-3-methyl anthraquinone (**14**) control reaction and reaction with addition of RslO9. The absorption at 254 nm on the y-axis is plotted against the retention time on the x-axis. UV-traces of control and enzyme condition are shifted to improve visibility.

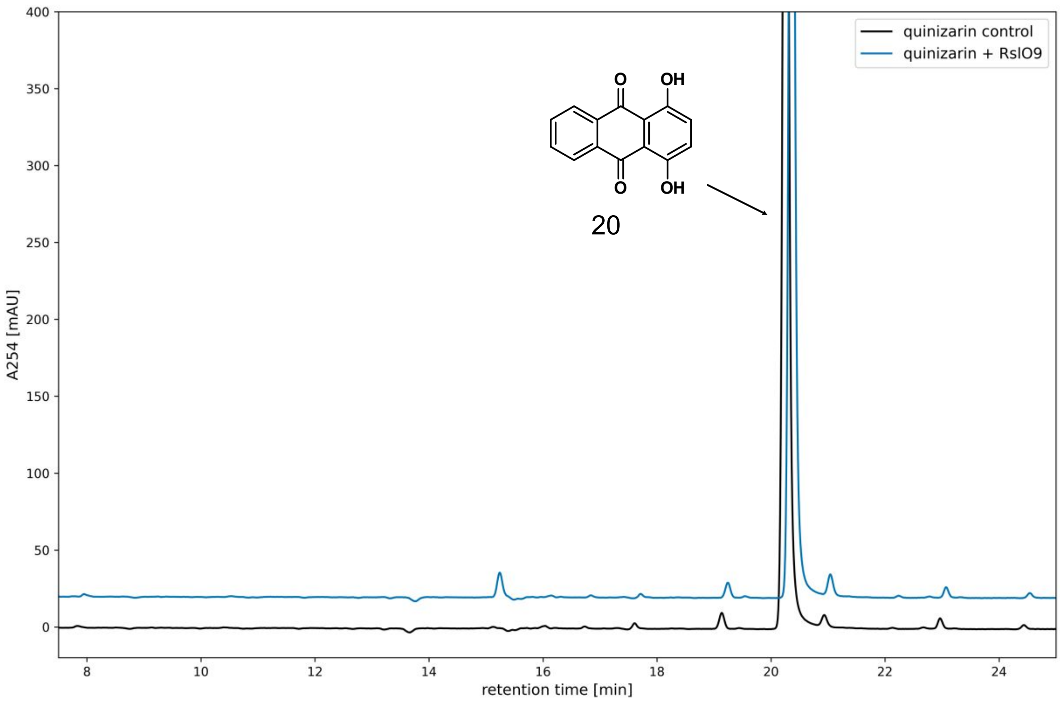

Figure S27: UV trace of RslO9 reaction with 20. 254 nm UV traces of the LC/MS measurements from quinizarin (**20**) control reaction and reaction with addition of RslO9. The absorption at 254 nm on the y-axis is plotted against the retention time on the x-axis. UV-traces of control and enzyme condition are shifted to improve visibility.

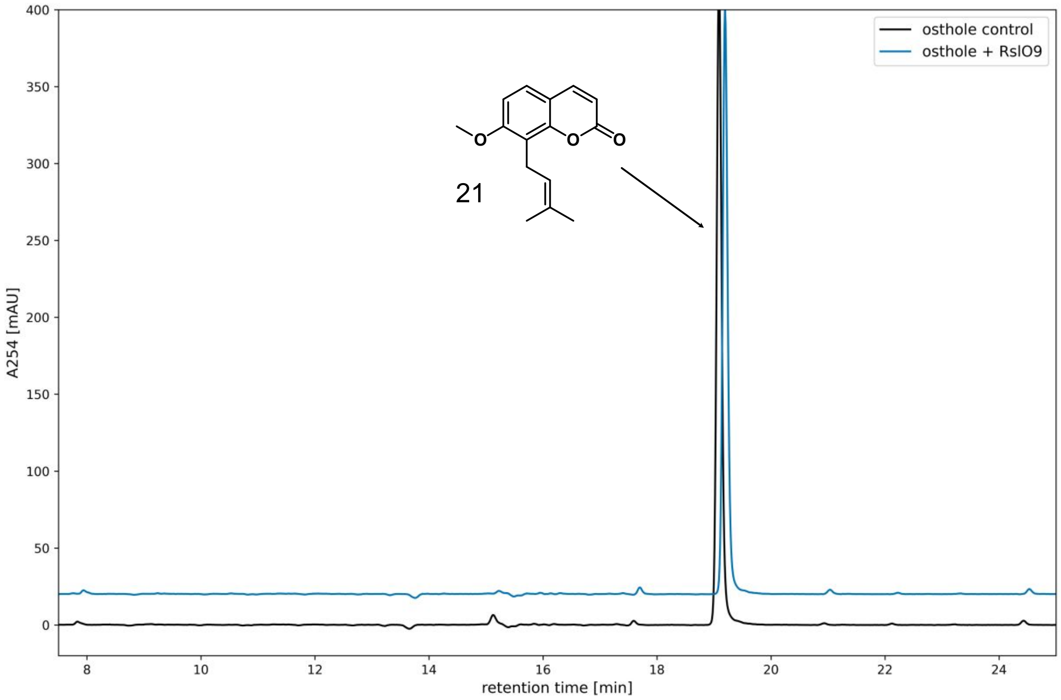

Figure S28: UV trace of RslO9 reaction with 21. 254 nm UV traces of the LC/MS measurements from osthole (**21**) control reaction and reaction with addition of RslO9. The absorption at 254 nm on the y-axis is plotted against the retention time on the x-axis. UV-traces of control and enzyme condition are shifted to improve visibility.

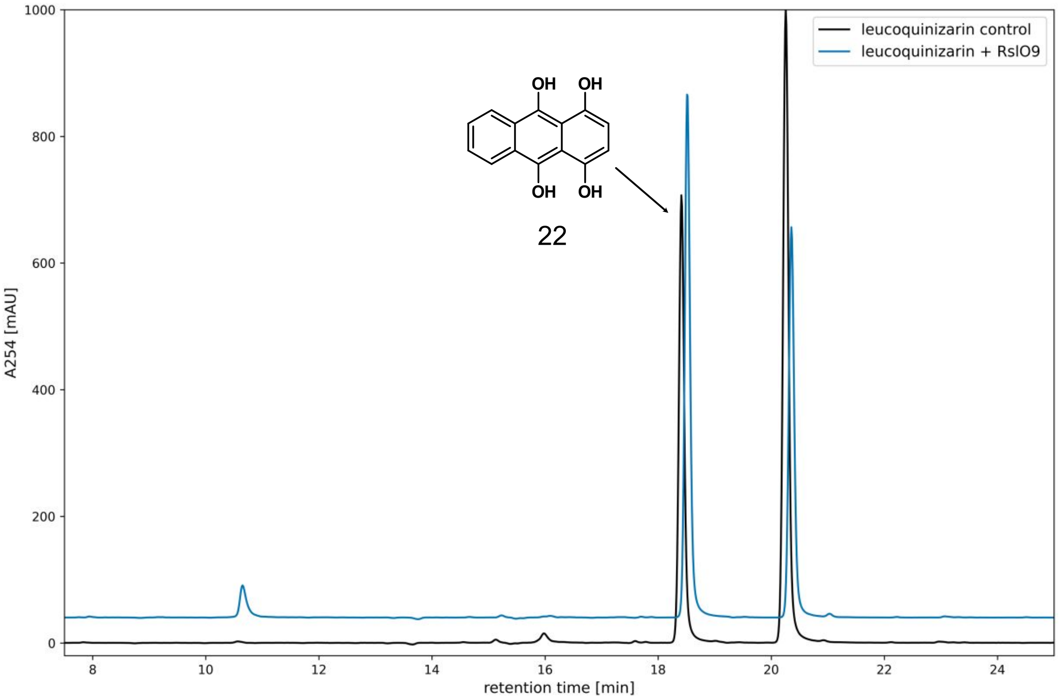

Figure S29: UV trace of RslO9 reaction with 22. 254 nm UV traces of the LC/MS measurements from leucoquinizarin (**22**) control reaction and reaction with addition of RslO9. The absorption at 254 nm on the y-axis is plotted against the retention time on the x-axis. UV-traces of control and enzyme condition are shifted to improve visibility (significant autooxidation to quinizarin was observed in the assay mixture (second major peak around 20 min).

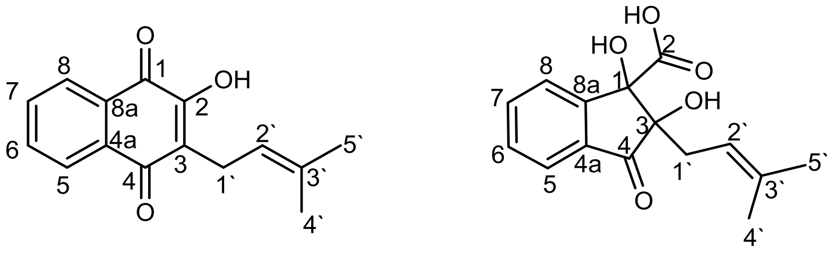

Figure S30: Numbering of 8 and 16. Representation of lapachol (**8**) and the Hooker intermediate (**16**) with respective atom numbering.

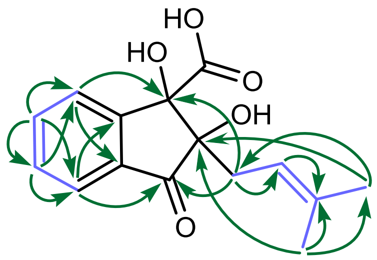

Figure S31: COSY and HMBC representation of 16. Representation of the HMBC (green arrows) and COSY (blue bonds) correlations of the Hooker intermediate (**16**) from the RslO9 reaction.

Table S5: NMR data for compounds 8 and 16 measured in DMSO-d_6

	lapachol (**8**)			Hooker intermediate (**16**)		
position	δ_C[(a)]	δ_H[(b)], mult.	HMBC[(a, b)]	δ_C	δ_H, mult.	HMBC
1	181.58		8	83.25		1, 1`
2	155.55		1`	172.80		
3	123.39		1`, 4`, 5`	87.53		1`, 4`, 5`
4	184.70		5, 1`	202.13		5, 6, 1`
4a	130.41		5, 6, 7	135.21		6, 7, 8
5	126.17	7.96, s	4, 4a, 6, 7	122.21	7.57, m	4, 7, 8, 8a
6	133.63	7.77, m	4a, 5, 8, 8a	129.14	7.45, m	4, 4a, 7, 8, 8a
7	134.98	7.82, m	4a, 5, 8, 8a	134.75	7.66, m	4a, 5, 6, 8, 8a
8	126.11	7.98, m	6, 7, 8a, 1	123.72	7.57, m	1, 4a, 5, 6, 7
8a	132.36		6, 7, 8	150.50		5, 6, 7
2-OH		11.02, br s				
1` (")	22.50	3.15, br d	2, 3, 4, 2`, 3`, 5`	34.46	1.98, dd	1, 3, 4, 2`, 3`, 5`
1`(')				34.46	2.45, m	1, 3, 4, 2`, 3`, 5`
2`	121.13	5.11, m	1`, 3`, 4`, 5`	118.96	4.92, s	1`, 3`, 4`, 5`
3`	132.46		1`, 4`, 5`	132.54		1`, 4`, 5`
4`	25.92	1.62, s	3, 2`, 3`, 5`	25.64	1.41, s	3, 2`, 3`, 5`
4a	130.41		5, 6, 7	135.21		6, 7, 8
5`	18.23	1.71, s	3, 1`, 2`, 3`, 4`	17.46	1.26, s	3, 1`, 2`, 3`, 4`

(a) Recorded at 125 MHz, (b) Recorded at 500 MHz, HMBC correlations are presented from proton(s) to the indicated carbon, The respective signal of DMSO-d_6 was used to reference the NMR spectra.

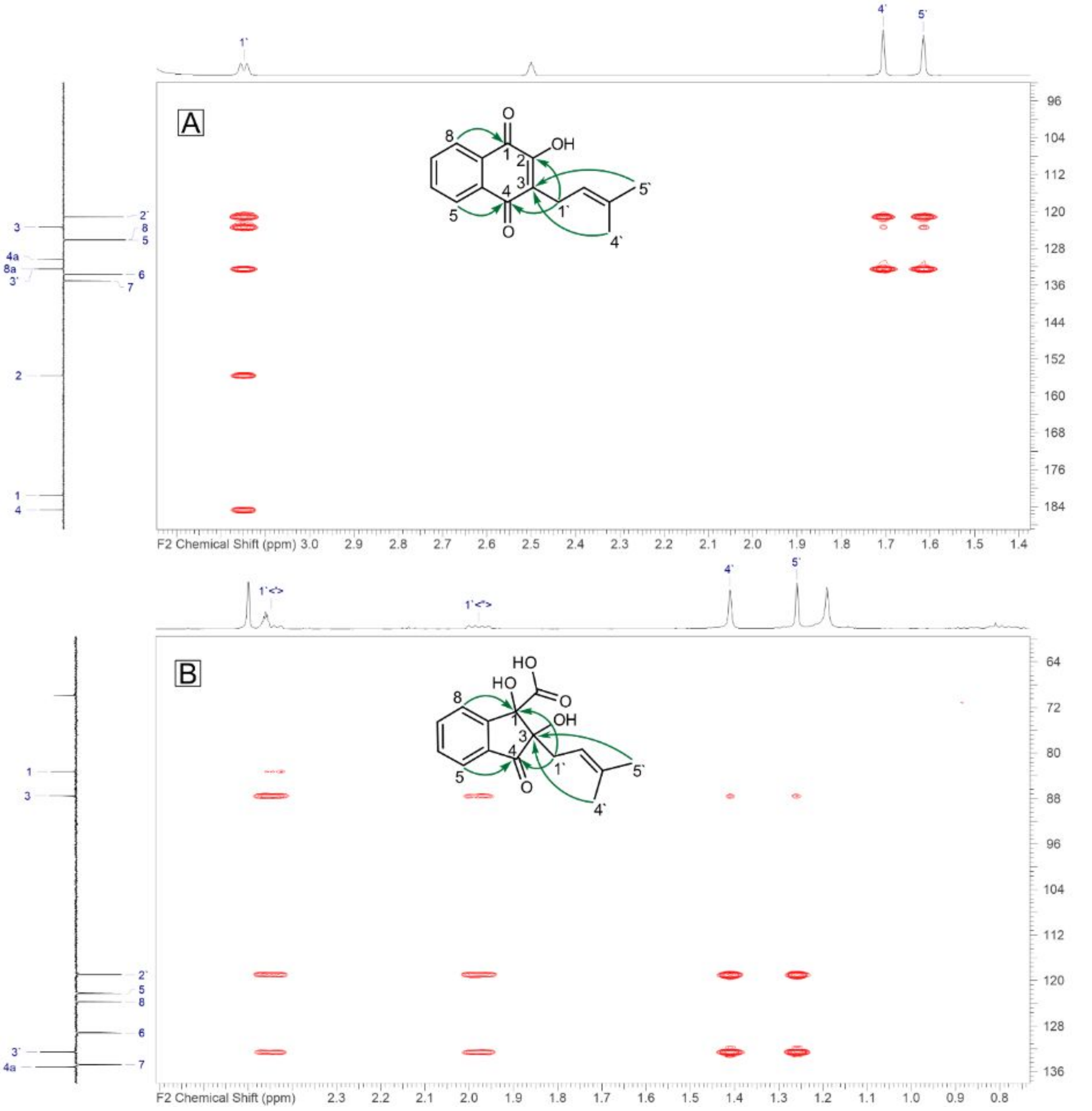

Figure S32: Representation of long range HMBC correlations of 8 and 16. Comparison of selected section of the HMBC spectrum (measured in DMSO-d6, on the same device) showing long range correlations of **8** (A) and the **16** (B) between atoms 3, 4` and 5`. Highlighted structure representation of the HMBC correlations (green arrows) for the substituent and the connectivity to the ring system.

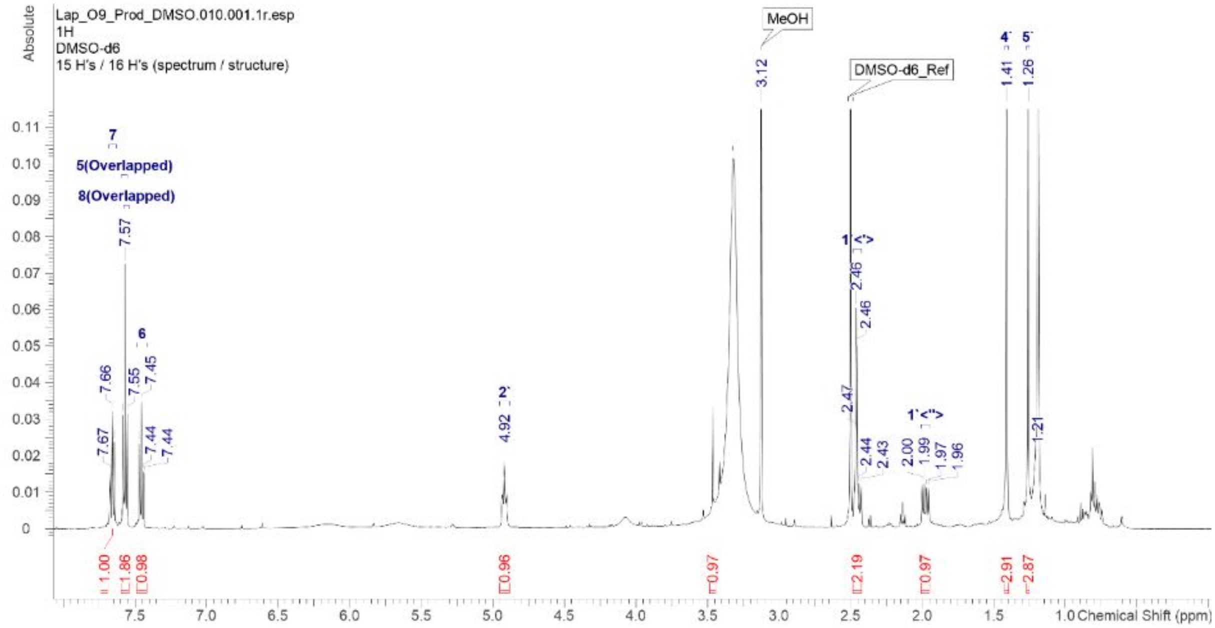

Figure S33: ^{1}H NMR spectrum of Hooker intermediate (16) (DMSO-d$_6$, 500 MHz).

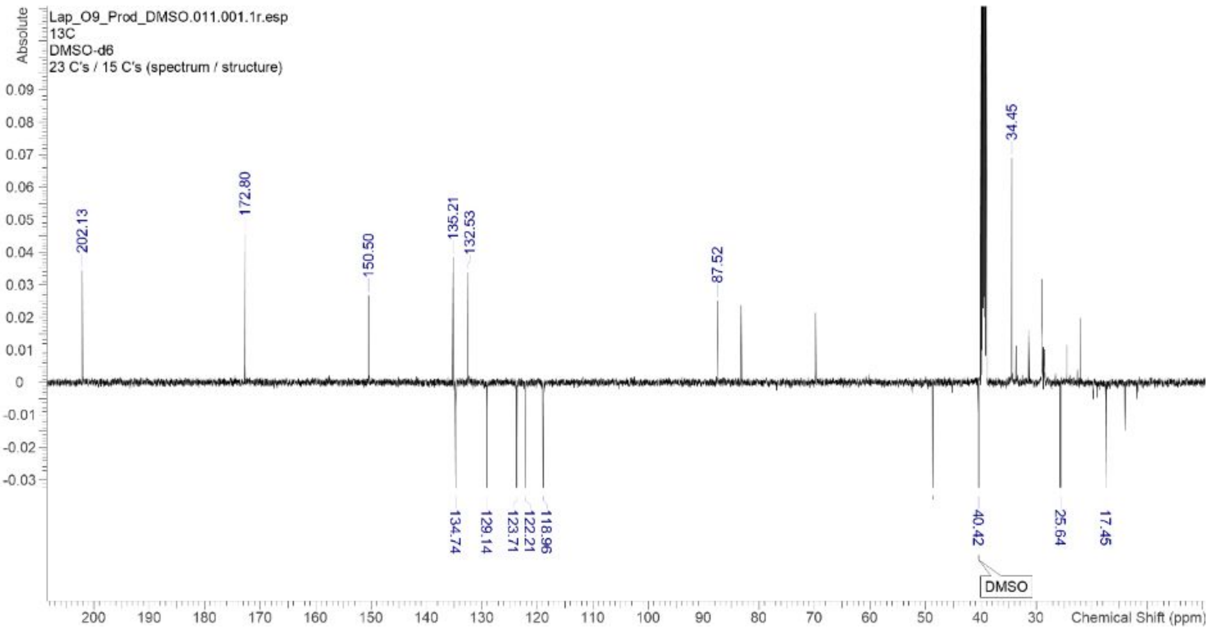

Figure S34: ^{13}C NMR spectrum of Hooker intermediate (16) (DMSO-d_6, 125 MHz).

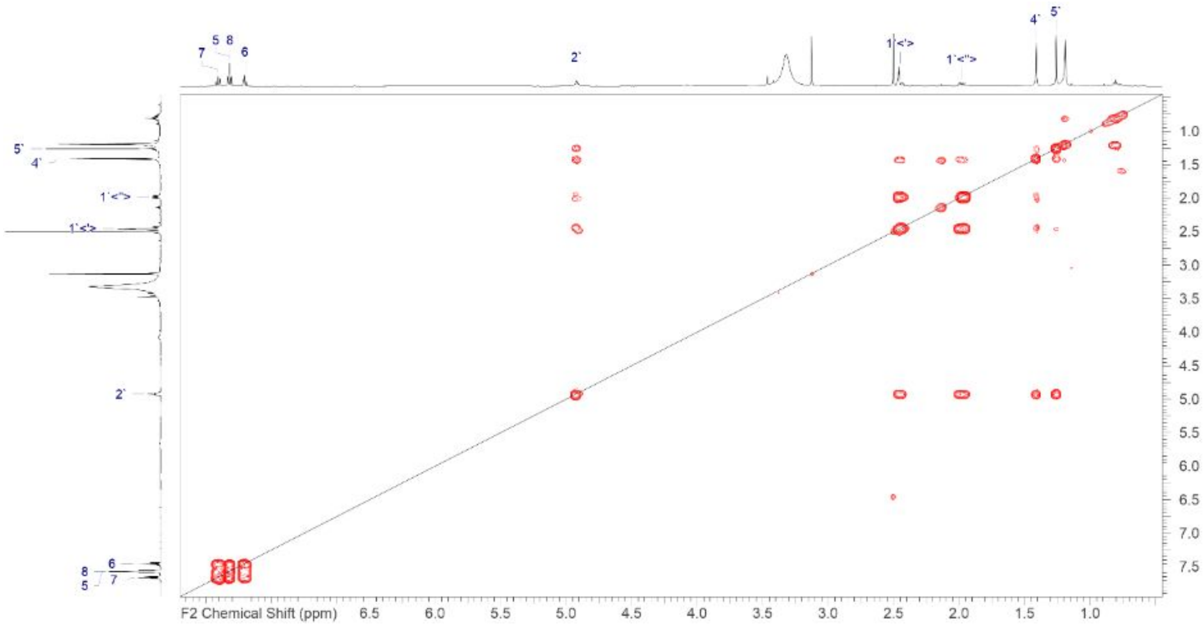

Figure S35: COSY NMR spectrum of Hooker intermediate (16) (DMSO-d$_6$, 500 MHz).

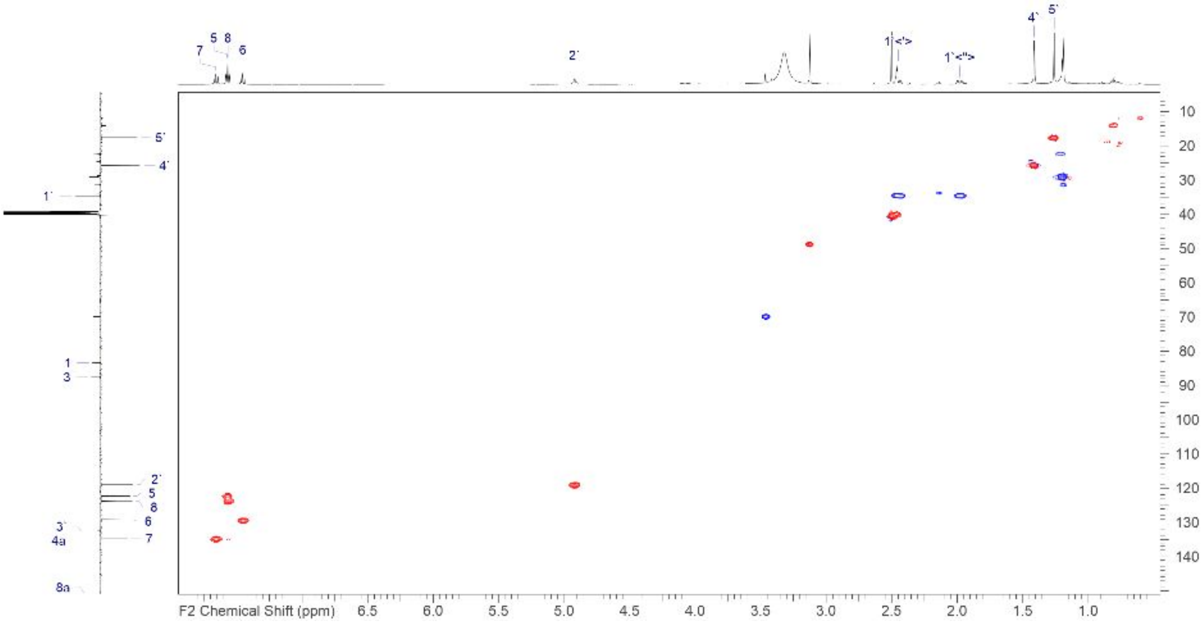

Figure S36: HSQC NMR spectrum of Hooker intermediate (16) (DMSO-d_6, 500 MHz).

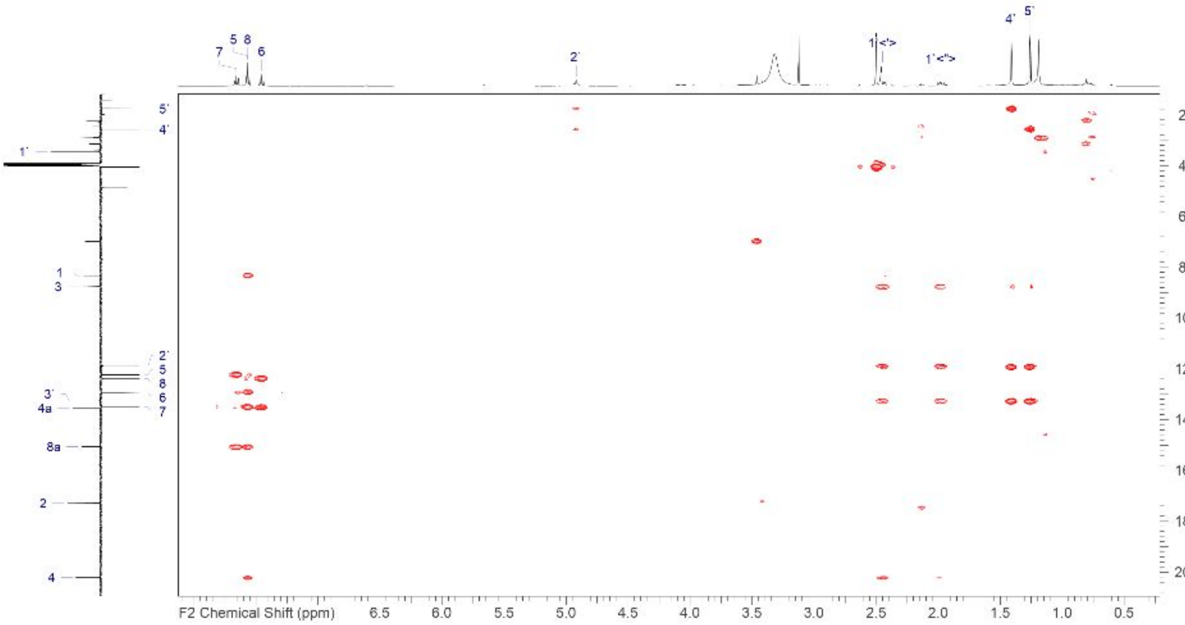

Figure S37: HMBC NMR spectrum of Hooker intermediate (16) (DMSO-d$_6$, 500 MHz).

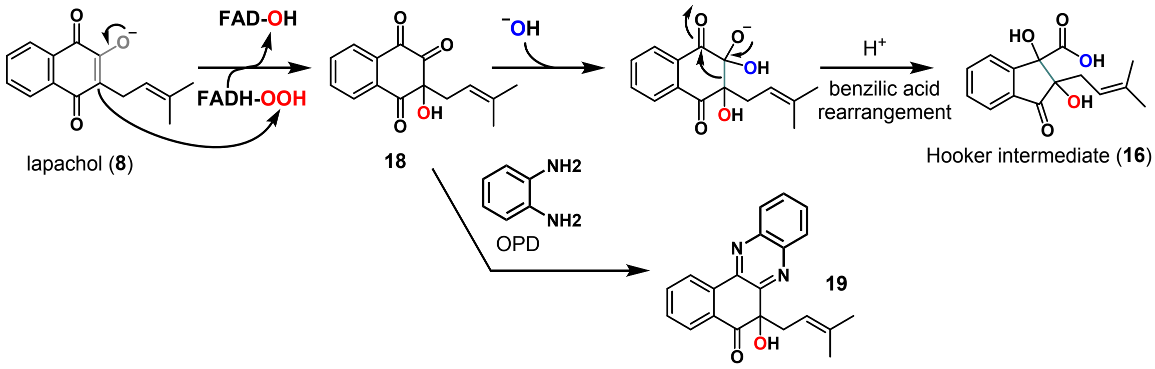

Figure S38: Schematic overview of RslO9 reaction with 8. Overview of the RslO9 reaction with lapachol (**8**) towards the Hooker intermediate (**16**). The diketo intermediate **18** can be captured by *ortho*-phenylene diamine (OPD) to yield **19**.

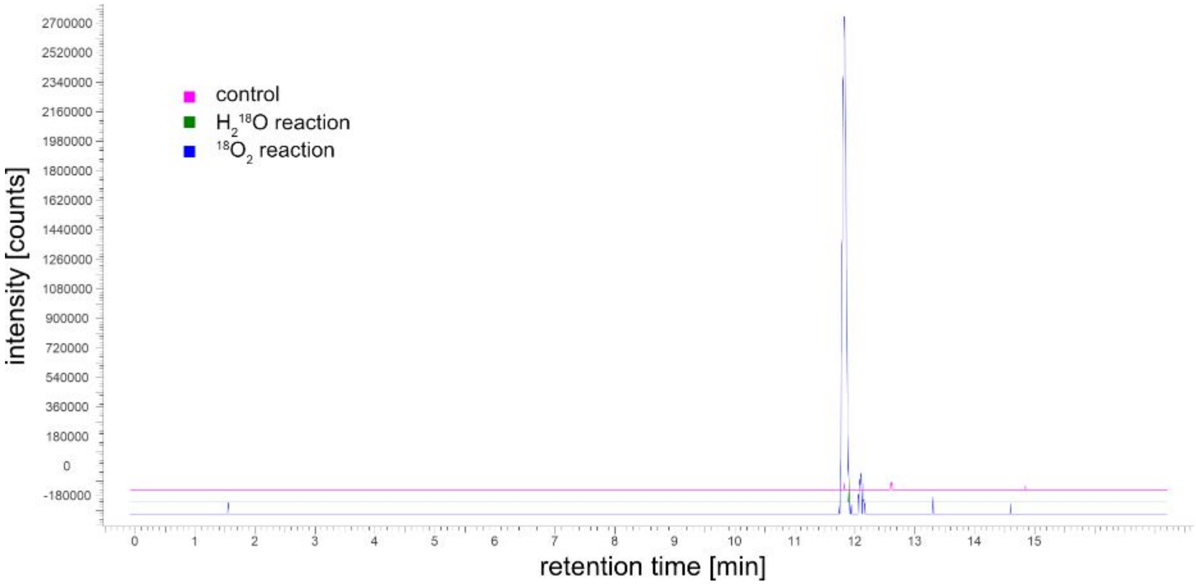

Figure S39: EIC spectra for RslO9 reactions for the formation of ^{18}O-labeled 19. Representation of the UHPLC-HRMS measurements of RslO9 reactions of lapachol **8** to the Hooker intermediate **16** with the addition of OPD to capture the diketo-intermediate **18** and form the phenazine derivative **19**. The extracted ion chromatograms of control (pink), $H_2{}^{18}O$ (green) and $^{18}O_2$ (blue) are shown for ^{18}O-labeled **19** with a calculated m/z of 333.1483 with a mass tolerance of 0.001 Da.

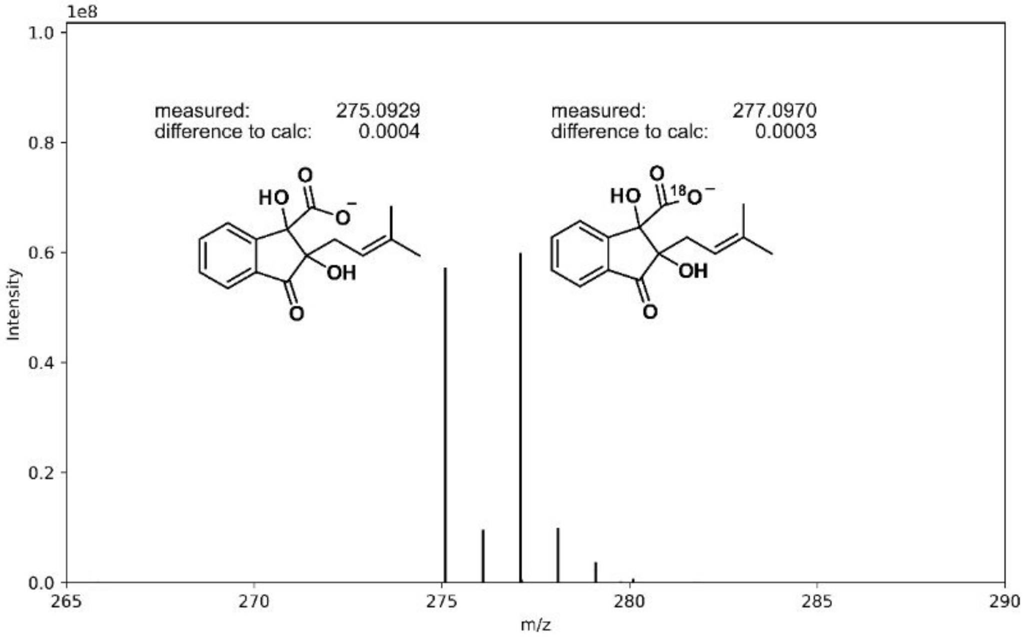

Figure S40: HRMS MS1 of 16 from $H_2{}^{18}O$ reaction. Mass spectrum of Hooker intermediate **16** from the conversion of **8** by RslO9 with 50% $H_2{}^{18}O$ in the assay mixture.

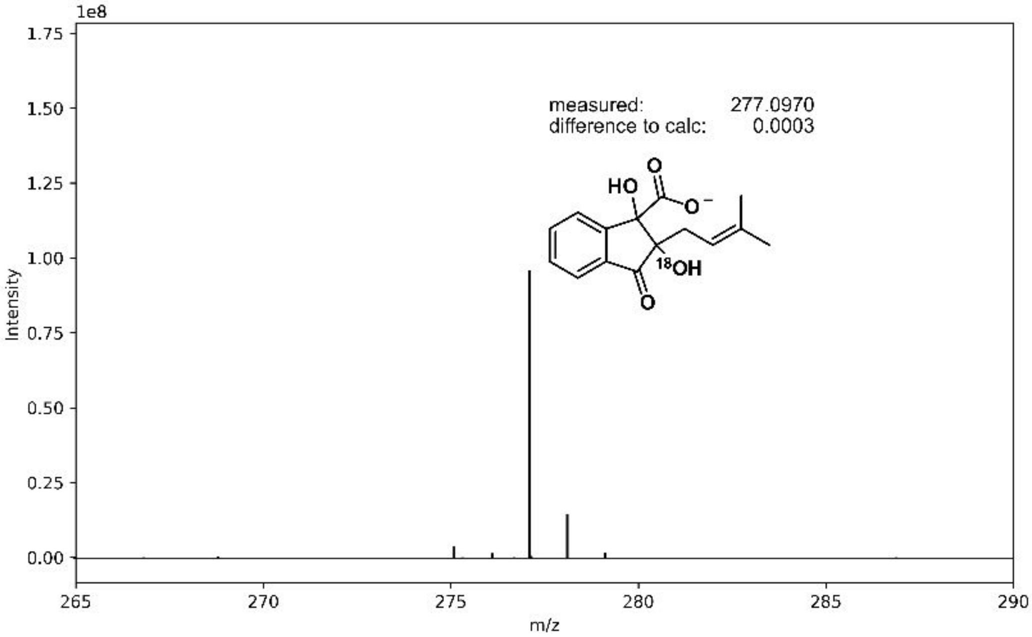

Figure S41: HRMS MS1 of 16 from $^{18}O_2$ reaction. Mass spectrum of Hooker intermediate **16** from the conversion of **8** by RslO9 with in $^{18}O_2$ atmosphere.

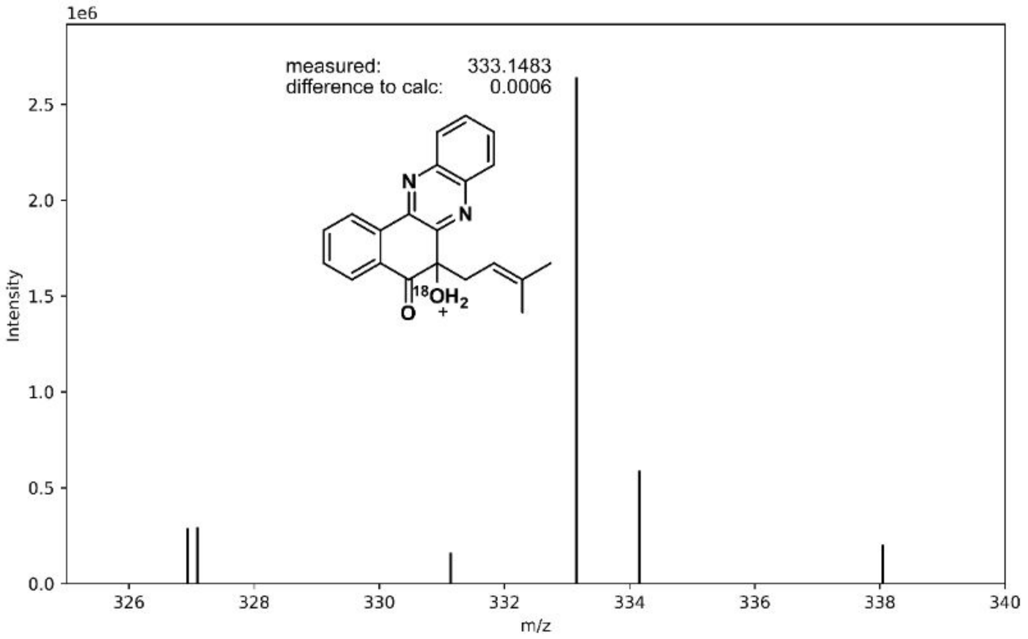

Figure S42: HRMS MS1 of 19 from $^{18}O_2$ reaction. Mass spectrum of OPD adduct **19** from the conversion of **8** by RslO9 with in $^{18}O_2$ atmosphere.

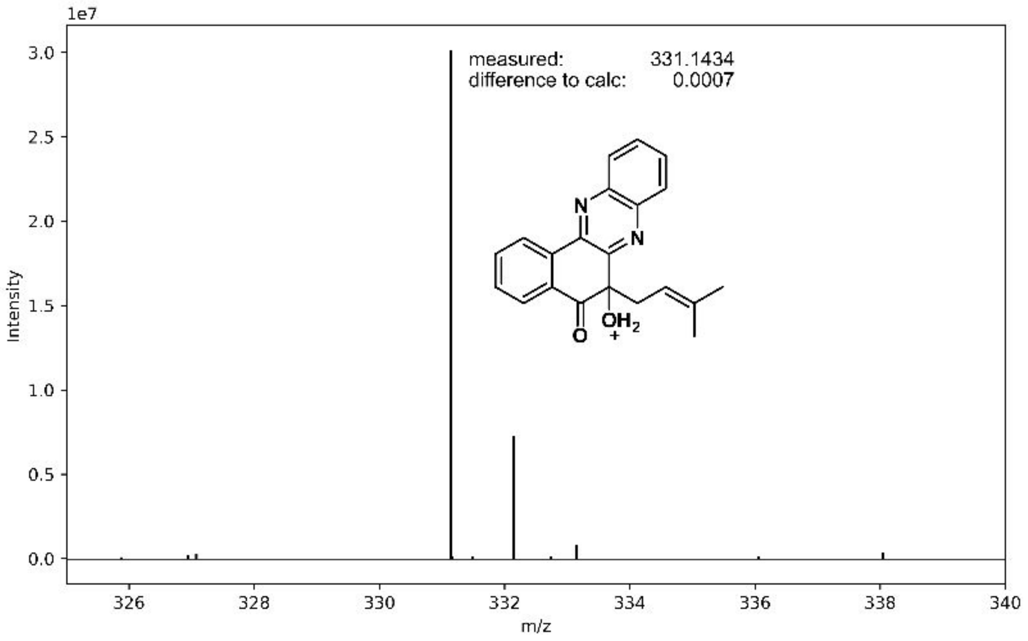

Figure S43: HMRS MS1 of 19 from $H_2{}^{18}O$ reaction. Mass spectrum of OPD adduct **19** from the conversion of **8** by RslO9 with 50% $H_2{}^{18}O$ in the assay mixture.

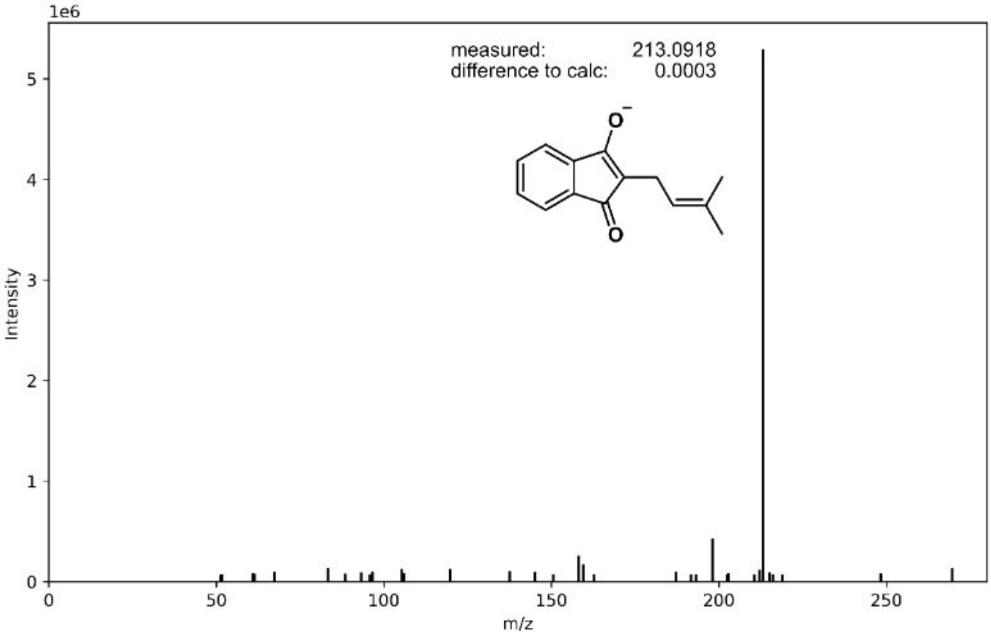

Figure S44: HRMS MS2 of 16. MS^2 mass spectrum of Hooker intermediate **16** from the conversion of **8** by RslO9 showing the major fragment.

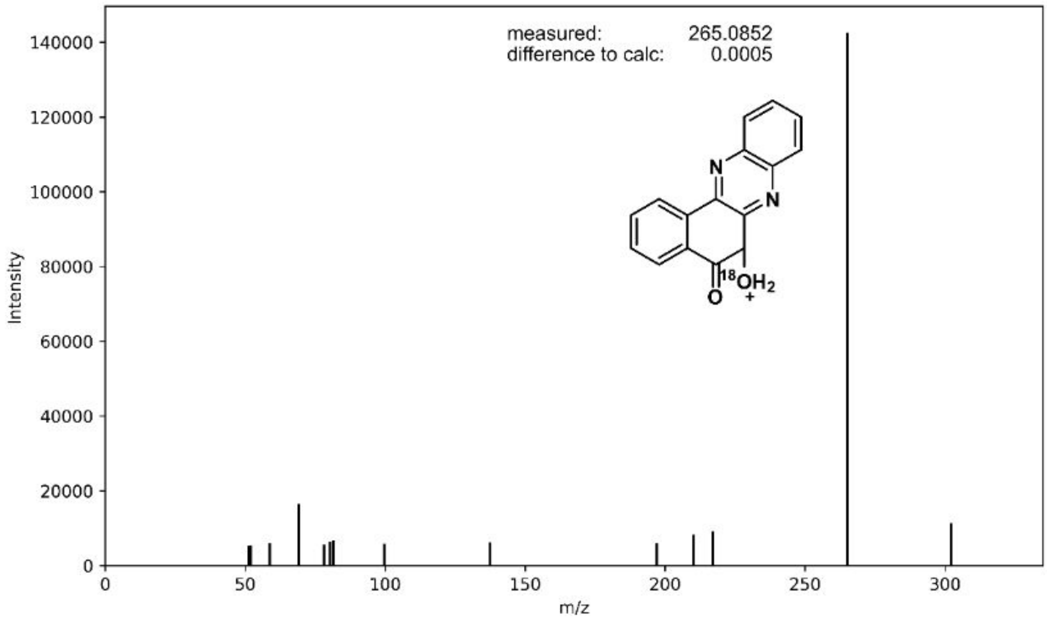

Figure S45: HRMS MS2 of 19 from the $^{18}O_2$ reaction. MS^2 mass spectrum of OPD adduct peak **19** from the conversion of **8** by RslO9 showing the major fragment performed in $^{18}O_2$ atmosphere.

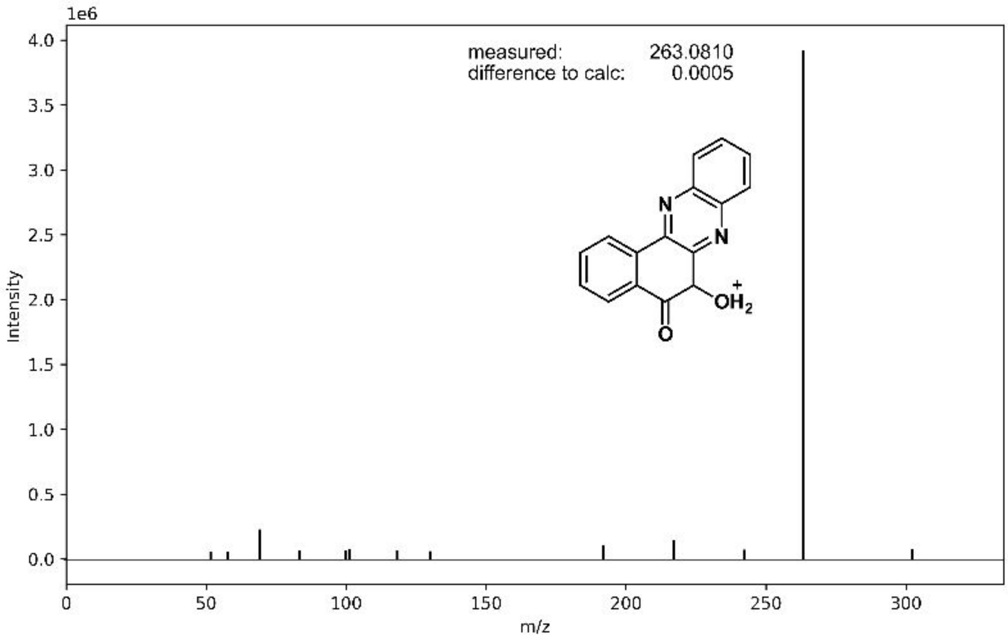

Figure S 46: HRMS MS2 of 19 from the $H_2{}^{18}O$ reaction. MS^2 mass spectrum of OPD adduct peak **19** from the conversion of **8** by RslO9 showing the major fragment performed with 50% $H_2{}^{18}O$ in the assay mixture.

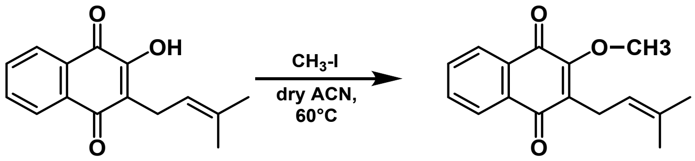

lapachol (**8**) methyl-lapachol (**20**)

Figure S47: Reaction overview of 8 methylation. Schematic representation of O-methylation of **8** with iodomethane to obtain methyl-lapachol (**20**).

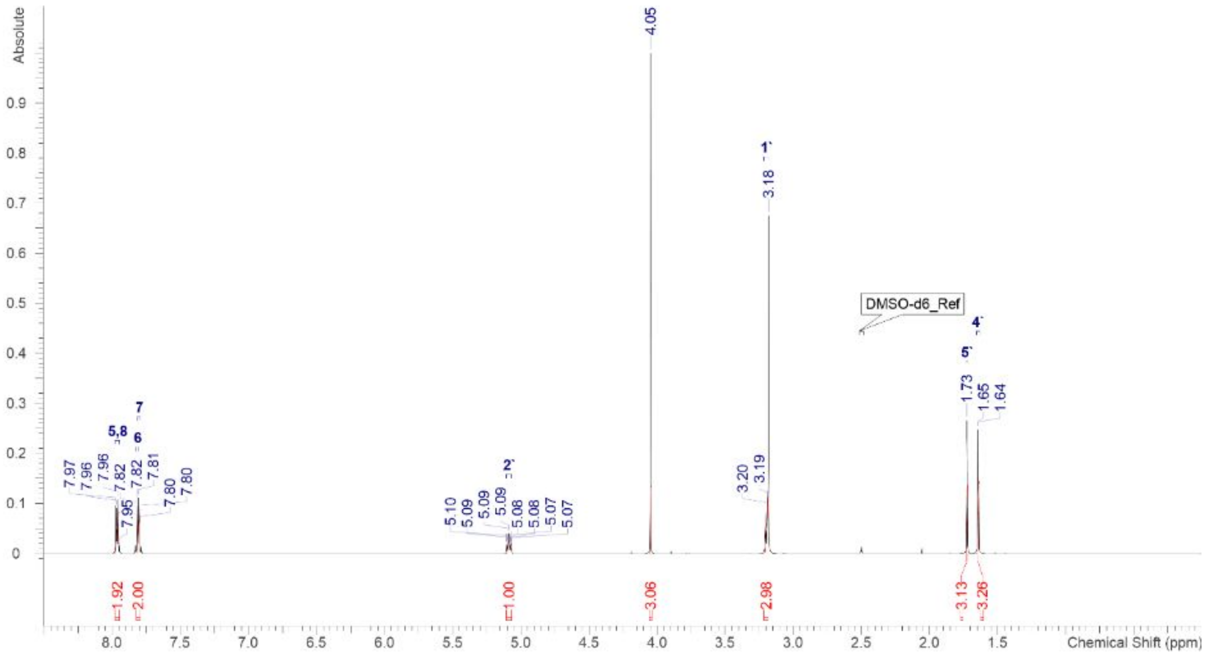

Figure S48: ^{1}H NMR spectrum of methyl-lapachol (20) (DMSO-d_6, 500 MHz).

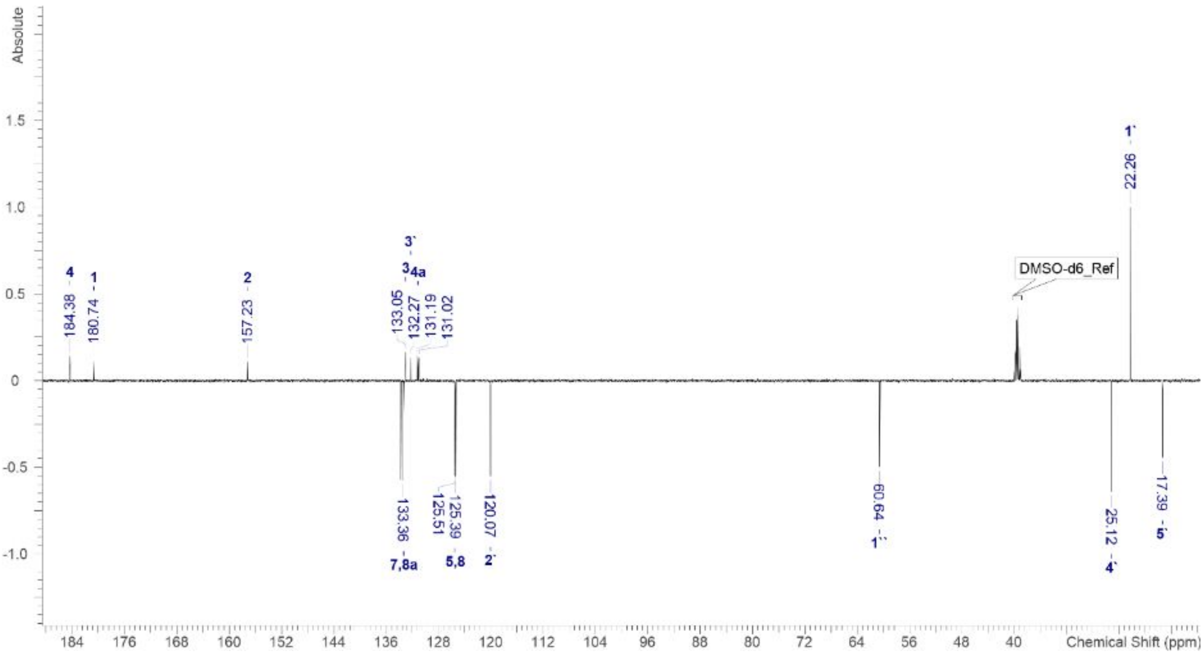

Figure S49: ^{13}C NMR spectrum of methyl-lapachol (20) (DMSO-d_6, 125 MHz).

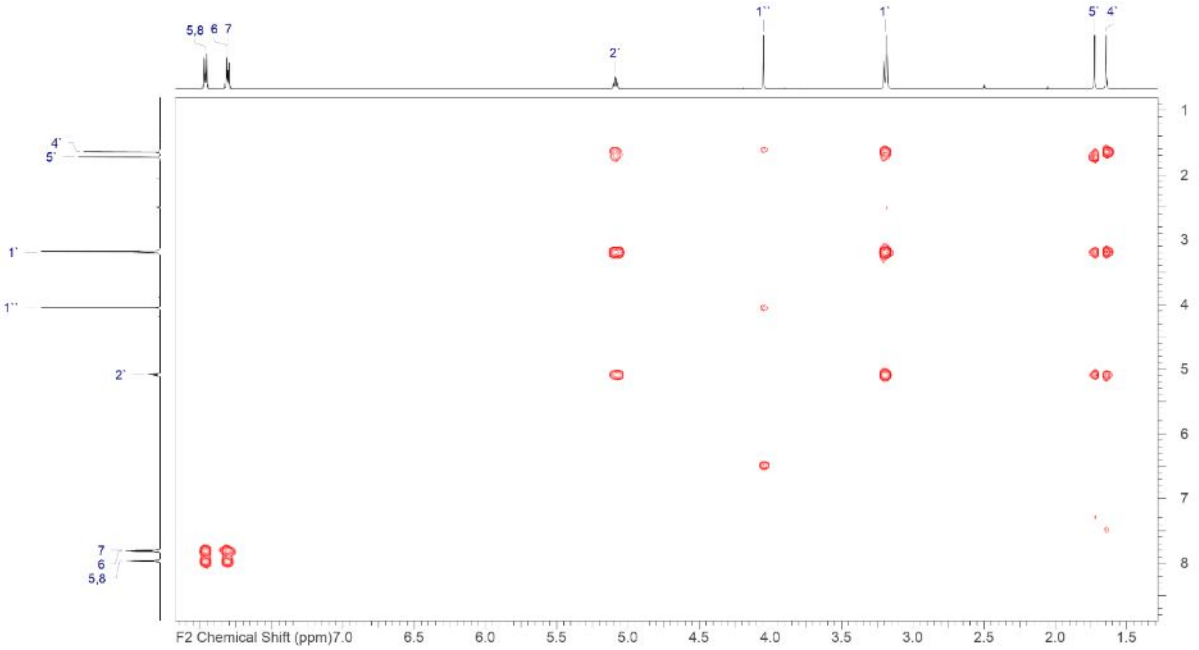

Figure S50: COSY NMR spectrum of methyl-lapachol (20) (DMSO-d_6, 500 MHz).

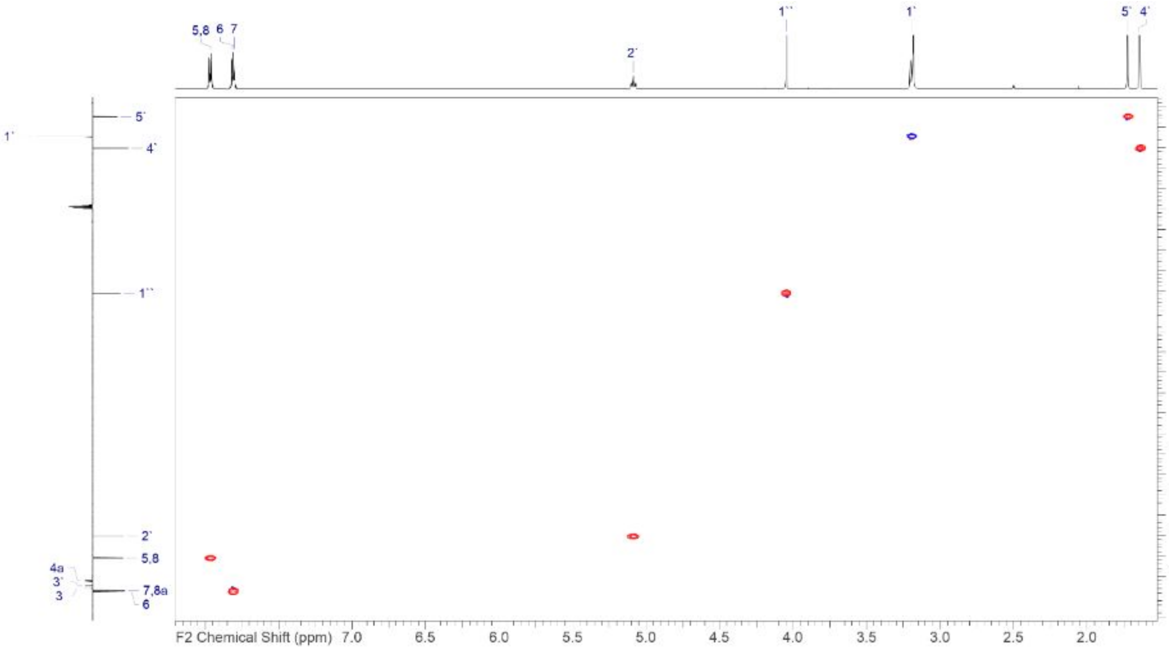

Figure S51: HSQC NMR spectrum of methyl-lapachol (20) (DMSO-d$_6$, 500 MHz).

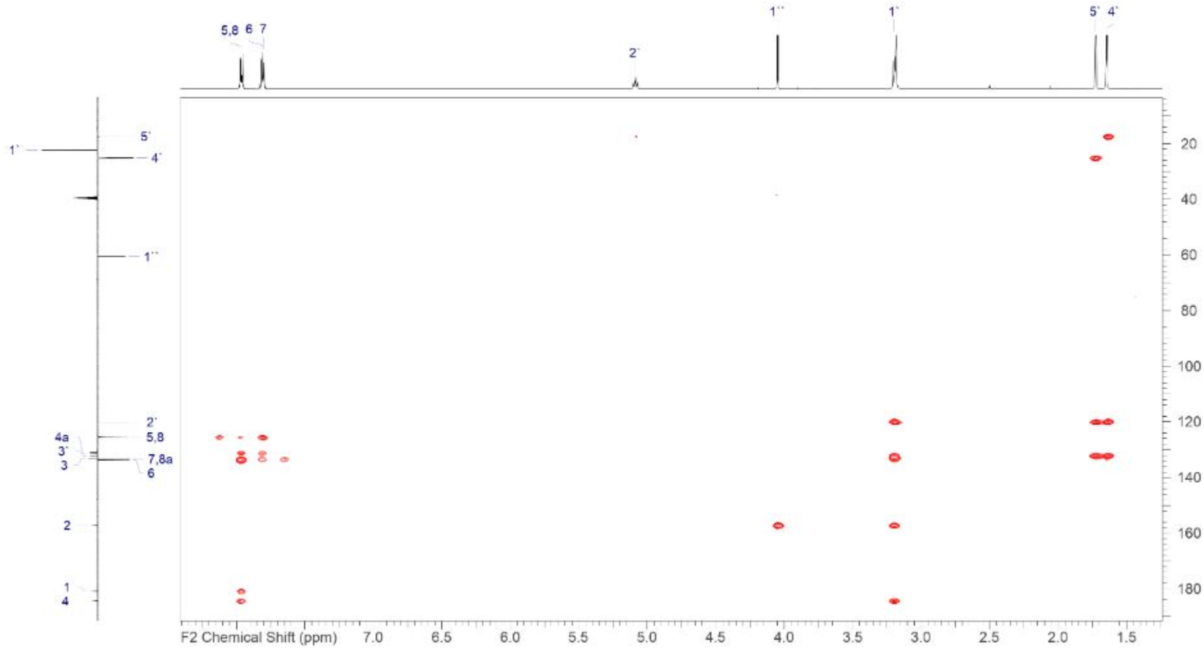

Figure S52: HMBC NMR spectrum of methyl-lapachol (20) (DMSO-d$_6$, 500 MHz).

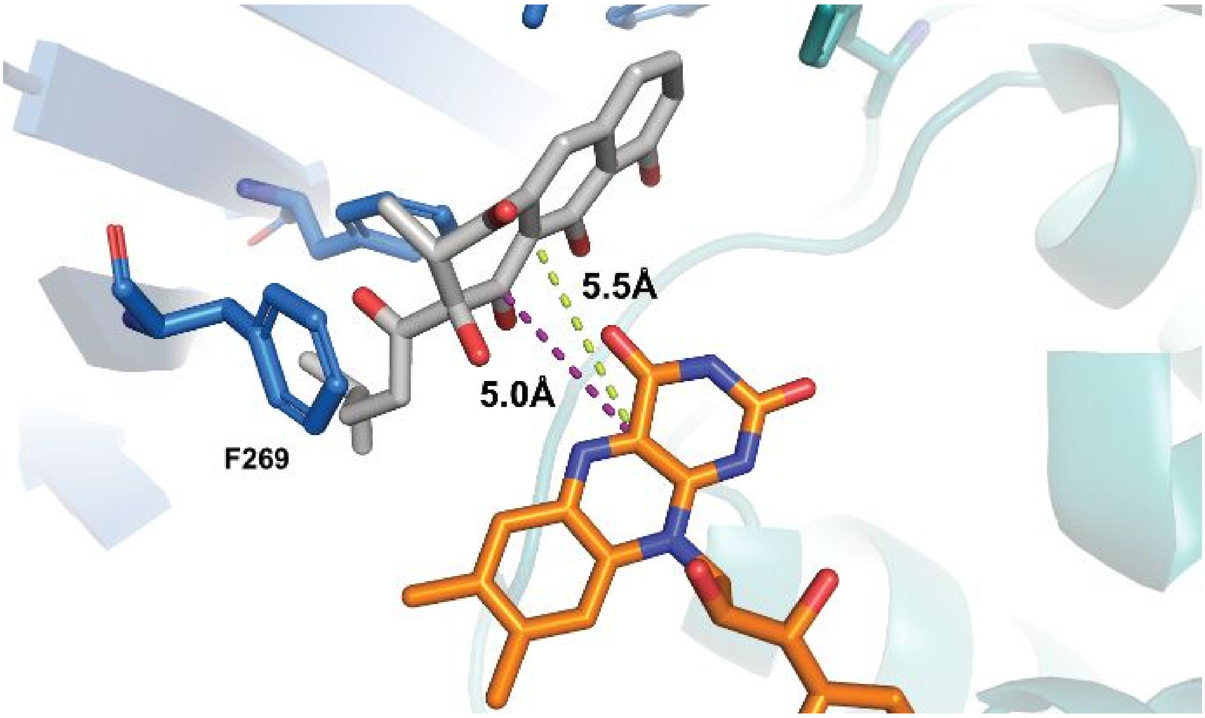

Figure S 53:Distance and angle representation of docked 5 relative to the FAD cofactor of RslO9. Presentation of the cartoon structure of RslO9 with the FAD-binding domain (green), the middle domain (blue) and the FAD cofactor shown in orange sticks. The distance between the Flavine-$C4_a$ (the position of the $C4_a$-OOH formation) and the substrates C4 and $C4_a$ are shown in purple and lime dashed lines respectively. Distances of measurements in Ångström are shown next to the dashed lines. The distance of 5.0 Å and angle almost perpendicular from the flavine-$C4_a$ to the C4 atom of **5** seems more favorable for an attack than the longer distance and distorted angle to the $C4_a$ of **5**.

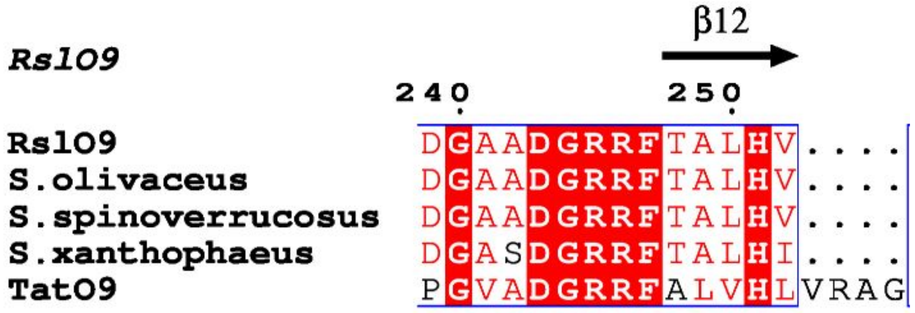

Figure S54: representation of active site MSA residues of RslO9 and homologs. Representation of active site region of the MSA of RslO9 and homologs of rishirilide producers or TatO9 from tatiomycin biosynthesis highlighting the conserved H251. MSA was generated using MEGA X and secondary structure elements of RslO9 are shown on top of the alignment, conserved amino acids are highlighted in red. Representation of the secondary structure above the alignment was generated via the ESPript 3.0 online server[3].

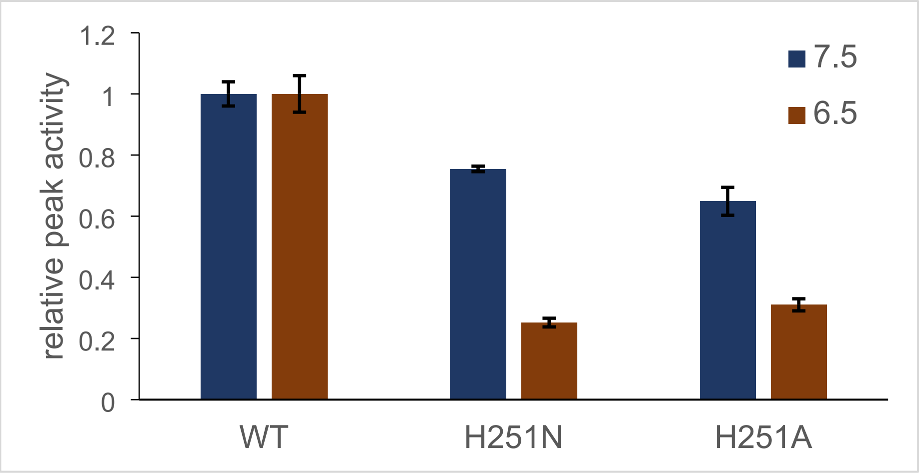

Figure S55: RslO9WT vs H251N and H251A activity comparison. *In vitro* activity of RslO9 wild type in comparison to the single amino acid substitution variants H251N and H251A. The graph shows the relative activity of the variants in comparison to the wild type based on the observed product peak from triplicate measurements. Variants are compared and normalized separately to wild type at pH 7.5 (blue bars) and pH 6.5 (brown bars).

Table S6: RslO9 QuickChange primer. Representation of QuickChange primer for site-directed mutagenesis of RslO9.

primer name	sequence
RslO9_H251N_fw	5´- CCGCGCTGAACGTGGGCCGCGCGG -3´
RslO9_H251N_rev	5´- CGCGGCCCACGTTCAGCGCGGTGAACCG-3´
RslO9_H251A_fw	5´- CCGCGCTGGCAGTGGGCCGCGCGG-3`
RslO9_H251A_rev	5´- CGCGGCCCACTGCCAGCGCGGTGAACCG-3´

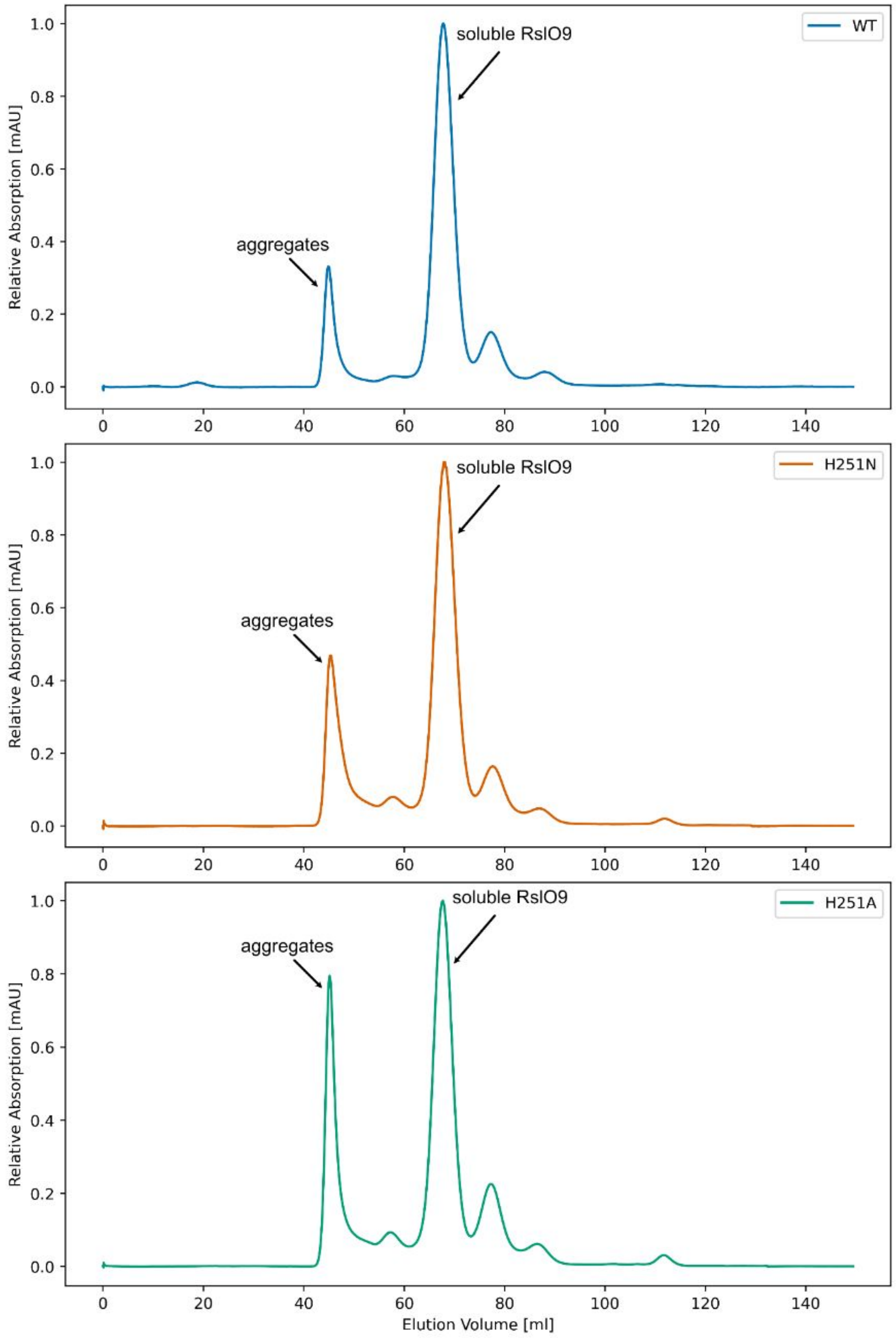

Figure S56: Size exclusion chromatograms of RslO9 H251 variatns vs RslO9 WT. Normalized size exclusion chromatography chromatograms for RslO9WT and variants H272N and H272A. The x-axis shows the elution volume in mL while the y-axis shows

the absorption at 280 nm normalized to the highest absorption. The folded protein fraction is represented by the peak with an elution volume of around 70 ml.

Supplementary references

(1) Tsypik, O.; Makitrynskyy, R.; Frensch, B.; Zechel, D. L.; Paululat, T.; Teufel, R.; Bechthold, A. Oxidative Carbon Backbone Rearrangement in Rishirilide Biosynthesis. *J. Am. Chem. Soc.* **2020**, *142* (13), 5913–5917.
(2) Dym, O.; Eisenberg, D. Sequence-structure analysis of FAD-containing proteins. *Protein Sci.* **2001**, *10* (9), 1712–1728.
(3) Robert, X.; Gouet, P. Deciphering key features in protein structures with the new ENDscript server. *Nucleic Acids Res.* **2014**, *42* (Web Server issue), W320–324.
(4) Toplak, M.; Saleem-Batcha, R.; Piel, J.; Teufel, R. Catalytic Control of Spiroketal Formation in Rubromycin Polyketide Biosynthesis. *Angew. Chem. Int. Ed. Engl.* **2021**, *60* (52), 26960–26970.
